# Supplementary material for: Special Issue: Climate Change and Health in Vietnam
Source: Glob Health Action. 2014 Dec 8;7:10.3402/gha.v7.26572. doi: 10.3402/gha.v7.26572 (PMC4265652; doi:10.3402/gha.v7.26572)
Supplement: Related texts

# Sub article: Primary healthcare system capacities for responding to storm and flood-related health problems: a case study from a rural district in central Vietnam

**DOI:** 10.3402/gha.v7.23007

**Published:** 2014-12-08

**Authors:** Hoang Van Minh, Tran Tuan Anh, Joacim Rocklöv, Kim Bao Giang, Le Quynh Trang, Klas-Göran Sahlen, Maria Nilsson, Lars Weinehall

**Affiliations:** 1 Institute for Preventive Medicine and Public Health, Hanoi Medical University, Hanoi, Vietnam; 2 Center for Health System Research, Hanoi Medical University, Hanoi, Vietnam; 3 Epidemiology and Global Health, Department of Public Health and Clinical Medicine, Umeå University, Umeå, Sweden

**Keywords:** climate change, storm, flood, health problems, health system, Vietnam, disasters, disease outbreaks, emergency medical services/utilization, public health

## Abstract

**Background:**

As a tropical depression in the East Sea, Vietnam is greatly affected by climate change and natural disasters. Knowledge of the current capacity of the primary healthcare system in Vietnam to respond to health issues associated with storms and floods is very important for policy making in the country. However, there has been little scientific research in this area.

**Objective:**

This research was to assess primary healthcare system capacities in a rural district in central Vietnam to respond to such health issues.

**Design:**

This was a cross-sectional descriptive study using quantitative and qualitative approaches. Quantitative methods used self-administered questionnaires. Qualitative methods (in-depth interviews and focus groups discussions) were used to broaden understanding of the quantitative material and to get additional information on actions taken.

**Results:**

1) Service delivery: Medical emergency services, especially surgical operations and referral systems, were not always available during the storm and flood seasons. 2) Governance: District emergency plans focus largely on disaster response rather than prevention. The plans did not clearly define the role of primary healthcare and had no clear information on the coordination mechanism among different sectors and organizations. 3) Financing: The budget for prevention and control of flood and storm activities was limited and had no specific items for healthcare activities. Only a little additional funding was available, but the procedures to get this funding were usually time-consuming. 4) Human resources: Medical rescue teams were established, but there were no epidemiologists or environmental health specialists to take care of epidemiological issues. Training on prevention and control of climate change and disaster-related health issues did not meet actual needs. 5) Information and research: Data that can be used for planning and management (including population and epidemiological data) were largely lacking. The district lacked a disease early-warning system. 6) Medical products and technology: Emergency treatment protocols were not available in every studied health facility.

**Conclusions:**

The primary care system capacity in rural Vietnam is inadequate for responding to storm and flood-related health problems in terms of preventive and treatment healthcare. Developing clear facility preparedness plans, which detail standard operating procedures during floods and identify specific job descriptions, would strengthen responses to future floods. Health facilities should have contingency funds available for emergency response in the event of storms and floods. Health facilities should ensure that standard protocols exist in order to improve responses in the event of floods. Introduction of a computerized health information system would accelerate information and data processing. National and local policies need to be strengthened and developed in a way that transfers into action in local rural communities.

Climate change contributes to a rise in the frequency and severity of natural disasters, especially storms and floods that can lead to a number of societal risks and health consequences (1–3). Health effects of climate extremes can be direct, such as drowning and injuries, or indirect and delayed, such as waterborne infections, acute or chronic effects of exposure to chemical pollutants released into flood waters, vector-borne diseases, mental health consequences, and food shortages (4–9). Storms and floods can also disrupt the capacity of healthcare systems to respond to health crises, and affect the overall quality of healthcare (10).

Vietnam is one of the most disaster-prone countries in the world. As a tropical depression in the East Sea, Vietnam is significantly affected by climate change and natural disasters. Over the past 20 years, natural disasters resulted in the loss of over 13,000 lives, and annual damage equivalent to an average 1% of the gross domestic product (GDP) (11). The most damaging and frequent disasters affecting Vietnam are tropical storms and floods. In 2007, an estimated 400 people died as the direct result of storms and floods. The economic loss to society was estimated around VND 11.5 billion (approximately USD 650 million) (12). The impact of climate change and associated events in Vietnam was projected to be serious and an imminent threat to poverty reduction, as well as the achievements of the Millennium Development Goals, which include health goals (13). Among other actions formulated to deal with problems associated with climate change and disasters, the Vietnamese government approved policies such as the 2007 National Strategy for Natural Disaster Prevention, Response and Mitigation to 2020, and the 2008 National Target Program in Response to Climate Change and is developing Law on Climate Change and Law on Disaster Management policies. The key objective of these polices is to establish a feasible action plan to deal effectively with climate change and disaster problems, including storm and flood-related issues (13).

The Vietnamese health system organizational structure consists of four levels that parallel the state administration system—central, provincial, district, and commune. At the central level, the Ministry of Health is the government agency that carries out the state management of healthcare protection and promotion, including preventive medicine, curative care, rehabilitation, traditional medicine, prophylactic and treatment drugs, cosmetics, food safety and hygiene, oversight of medical equipment, and management of public services under ministry control. At the provincial level, the provincial health department is a professional agency managed by the Provincial People's Committee, and works to advise the Provincial People's Committee on state management of local healthcare protection and promotion. The provincial health department performs tasks and duties as authorized by the Provincial People's Committee and legal regulations. The Provincial People's Committee controls the direction, organizational management, payroll, and operations of the provincial health department. The provincial health department is also under Ministry of Health control of technical directions, guidance, monitoring, and inspections. At the district level, the district health bureau is a professional agency under management of the District People's Committee that works to advise the District People's Committee on state management of local healthcare protection and promotion, and performs designated tasks and obligations as authorized by the District People's Committee and provincial health department. The District People's Committee controls the district health bureau in terms of direction, organizational management, payroll, and operations. District health bureau is also under provincial health department control of technical directions, guidance, monitoring, and inspections. The district level also has district hospitals (including polyclinics) and district centers for preventive medicine. The district centers for preventive medicine recently split from district health centers and are under provincial health department stewardship and management. At the commune level, the commune health center is the first formal point of healthcare contact with the government healthcare system. The commune health center provides primary healthcare services, conducts early detection of epidemics, provides care for common diseases and deliveries, mobilizes people to use birth control, teaches preventive hygiene practices, and manages health promotion. The commune health center is responsible to the district health bureau and the Commune People's Committee for local healthcare protection and promotion, and receives technical guidance from the district hospitals. The commune health center also supervises village health workers who are active close to homes and worksites. Every village has a village health worker with 3–9 months of training (14).

The Vietnamese healthcare system is assigned primary responsibility for prevention and response to climate change and disaster-related health issues. The primary healthcare system (including district and commune levels) is the first site of contact between individuals, the family, and community with the national health system. Primary healthcare brings health care as close to where people live and work as possible, and constitutes the first element of a continuing healthcare process. The primary healthcare system is expected to be the frontline for dealing with climate change and natural disaster-related health issues, particularly in rural settings (15).

Extreme weather and climate events interact with exposed and vulnerable human and natural systems and can lead to disasters (3). The concept of adaptive capacity has existed for decades (16–18). However, the most recent definition adopted by the Intergovernmental Panel on Climate Change is ‘the ability of a system to adjust to climate change (including climate variability and extremes) to moderate potential damages, to take advantage of opportunities, or to cope with the consequences’ (19, 20). Current conceptual underpinnings of adaptive capacity are most closely associated with the Intergovernmental Panel on Climate Change characterization of adaptation as an ‘adjustment in natural or human systems in response to actual or expected climatic stimuli or their effects’. Successful adaptation should result in an equal or improved situation when compared with the initial condition, while less successful responses (such as coping) will allow for short-term recovery but continued vulnerability. System coping capacity, or capacity of response is also called adaptive capacity (21, 22). The Intergovernmental Panel on Climate Change distinguishes coping capacity or response from adaptive capacity, and considers both as components of system resilience. Adaptations are referred to as system restructuring after response (21). Some authors apply ‘‘coping ability’’ to short-term capacity or the ability to just survive, and employ ‘‘adaptive capacity’’ for long-term or more sustainable adjustments (23). In general, response capacity is the system's ability to adjust to a disturbance, moderates potential damage, takes advantage of opportunities, and copes with the consequences of the occurring transformation. Capacity of response is a system attribute that existed prior to the perturbation. Broadly speaking, adaptive capacity denotes the ability of a system to adjust, modify, or change its characteristics or actions to moderate potential damage, take advantage of opportunities, or cope with the consequences of shock or stress (24).

Knowledge of the current capacity of the primary healthcare system in Vietnam to respond to health issues associated with storms and floods is important for national policy making. However, there has been little scientific research in this area. The objective of this research is to assess capacity of the primary healthcare system in a rural district in central Vietnam to respond to health issues associated with storms and floods. The key research question was ‘How capable is the primary healthcare system in rural Vietnam to respond to health issues associated with storms and floods, in terms of six system building blocks?’. These included: 1) service delivery, 2) policy/governance, 3) healthcare financing, 4) human resources, 5) information and research, and 6) medical products and technology. Research results are expected to be used by relevant stakeholders and policymakers in Vietnam to bridge national policies with local context and capacities during planning, management, and decision-making.

## Methods

### Study design

This was a cross-sectional, descriptive research study that used quantitative and qualitative approaches. Quantitative methods used self-administered questionnaires. The questionnaires allowed respondents to answer *very good, good, fair, bad, or very bad*. Qualitative methods (in-depth interviews and focus groups discussions) were used to broaden understanding of the quantitative material and to acquire additional information on actions taken. In-depth interviews collected data from healthcare staff at health facilities on different levels. We sought information from the perspective of the health service providers. Focus group discussions were used to expand information from a broader group of informants. Focus group discussions were organized for people representing different parts of civil society, such as the Women's Association, Veterans Association, Farmers Association, Youth Union, and Police. At the commune level, these individuals were local representatives identified through the Commune People's Committee.

### Study scope

We assessed capacity of the study area primary care system (district and commune health organizations) to respond to storm and flood-related health consequences, based on the World Health Organization (WHO) model of six primary care system building blocks: 1) service delivery, 2) policy/governance, 3) healthcare financing, 4) human resources, 5) information and research, and 6) medical products and technology (25).

### Study area

PhuVang district in Thua Thien-Hue province, located in the North Central Coast region of Vietnam, was selected for this study. PhuVang is a rural district that covers an area of 280 km^2^. As of 2010, the total PhuVang population was 171,363. The district was selected because it is a location where storms and floods frequently occur. In 2012, the PhuVang district healthcare organization included PhuVang district hospital with more than 80 beds, PhuVang district health center, PhuVang District Health Bureau, two inter-communal polyclinics, 20 commune health centers, and a network of village health workers.

### Study sample

Health facilities and staff at the district and commune levels from the PhuVang district primary healthcare system were studied. As the provincial health system was responsible for managing and supervising primary healthcare facility activities, interviews were also done with representatives from the Provincial Department of Health and centers for preventive medicine. Focus group discussions were done with local representatives from other sectors and community organizations. The study sample is presented in Table 1.

**Table 1:** The study sample

	Province		District			Commune			
	
	Provincial department of health	Center for preventive medicine	Center for preventive medicine	Hospital	Health Bureau	Health center^a^	Health staff	Non-health sector^b^	Total
Self-administered questionnaires	–	–	1	1	–	20	–	–	22
In-depth interviews	1	1	1	1	1	5	–	–	10
Focus group discussions	–	–	–	–	–	–	5	5	10

a PhuVang District has 20 health centers.

b Representatives from the Committee for Flood and Storm Control, Agriculture and Rural Development, Hydrometeorology Unit, TV/radio station, community organizations (including the Women's Association, Veterans Association, Farmers Association, Youth Union), and local people.

### Research tools

Research tools were a self-administered questionnaire, guidelines for in-depth interviews, and guidelines for focus group discussions. They were developed by a team of experienced researchers from the fields of medicine, epidemiology, and social medicine. The tools were pilot-tested and calibrated before official use.

### Data collection

The study was conducted between January and April 2013. Data collection was conducted by the research team, and consisted of professionals with experience in public health and health systems. The research team visited selected health facilities in the study area to collect the necessary data.

### Data management and analyses

EpiData 3.1 and Stata10 were used to enter and analyze quantitative data. Analysis of the qualitative data was inspired by descriptive content analysis techniques, which focus on the manifest content (i.e. look at the most obvious and straightforward meanings of a text) (26). Data were organized into six themes, corresponding to the six WHO building blocks listed above (25).

### Ethical considerations

Permission to conduct this study was approved by Hanoi Medical University and Thua Thien-Hue provincial health authorities. Informed consent was obtained from each informant.

## Results

### Service delivery

#### Prevention activities

For the prevention activities, respondents were asked to list prevention activities implemented for storm and flood-related health problems at the local health facilities and how the health facilities respond to storm and flood-related health problems. The quantitative survey showed that different population-based health promotion activities intended to improve local knowledge about storm and flood-related health problems was regularly implemented before, during, and after the storm and flood seasons. The main health promotion activity topics were about health risks associated with storms and floods, prevention and first-aid solutions, water, sanitation, nutrition issues, and disinfectant techniques after hazardous events. These prevention activities were implemented by the district health center, the commune health center, and the village health worker network through community meetings and public loudspeaker announcements. Respondents from 18 of 20 of the studied facilities (90%) rated their prevention activities as fair or good. Only 2 of 20 (10%) reported that they performed their prevention tasks poorly. The main difficulties encountered while implementing prevention activities were a lack of staff and funding. Laboratory equipment for disease surveillance and outbreak confirmation was insufficient. In addition, water and sanitation systems were poor, which made the prevention work more difficult. Findings from focus group discussion also showed that the preventive medicine team efforts were appreciated by the community. Focus group discussions showed that preventive medicine services (counseling on prevention and first-aid for storm and flood-related health problems, vaccinations against six preventable diseases, including diphtheria, tetanus, pertussis, poliomyelitis, measles and tuberculosis) were accessible to the local population and brought health benefits to the community.

#### Treatment activities

For treatment activities, respondents were asked to list treatment activities used for storm and flood-related health problems at the health facilities, rate the treatment activities, the number of patients the health facility treated during the past 5 years, and advantages and disadvantages of treatments for storm and flood-related health problems at the health facility. Quantitative and qualitative data revealed that emergency plans were established before storm and flood seasons at district and commune health facilities. At district hospitals, ambulance and emergency services were prepared. At the commune health station, first-aid services were readily available. Village health worker networks were provided with some medicines, supplies, and basic medical equipment needed for first-aid. All the interviewees reported that they were confident and happy with the quality of the first-aid services because standardized first-aid kits and protocols were available. All of the health staff had a chance to be involved in disaster simulation exercises to test response mechanisms. Most (79.9%) considered the treatments for common illnesses (e.g. dermatitis, skin infections, fungal infections, conjunctivitis, digestive problems, diarrhea) as fair or good. However, most (72.9%) raised concerns about the availability and quality of surgical services provided at the district hospital (by regulation, surgical services were not provided at commune health centers). Thirty-six percent of study respondents reported that treatment services (especially surgical operations) were sometimes not available during storm and flood seasons. Sixty-three percent rated the emergency referral system as poor. The same opinions on availability and quality of common services, especially of surgical operation services and emergency referral systems, were also identified in the focus group discussions with representatives from other sectors, community organizations, and local people. Building damages, electrical outages, and inadequacy of professional staff were reported as the main causes for primary healthcare system dysfunction during storm and flood seasons. Table 2 presents quantitative survey results of selected diseases or illnesses reported by the commune health centers before, during, and after the 2012 storm and flood season.

**Table 2:** Selected disease and illness cases reported by the CHC before, during, and after the 2012 storm and flood season, presented as average number of cases per month per center

Selected disease	Before (July–August 2012)	During (September–October 2012)	After (November–December 2012)
Injury	1.3	3.8	2.1
Conjunctivitis	1.6	1.4	3.4
Skin diseases (pruritus)	2.1	2.2	7.3
Gastrointestinal diseases (diarrhea, cholera, dysentery)	2.3	3.5	7.8

### Policy and governance

Respondents were asked about the availability of instructive documents that requested participation in prevention activities for storm and flood-related health problems, the frequency and timeliness in which the health facilities received those documents, and advantages and disadvantages to implementation of the instruction and guidance documents for prevention activities. Similar questions were asked about treatment activities.

While the Law on Climate Change and the Law on Disaster Management Policies were being developed, the key documents underpinning disaster risk reduction policies and strategies were the Ordinance on Prevention and Control of Floods and Storms (adopted by the Standing Committee of the 9th National Assembly of the Socialist Republic of Vietnam in 1993), the 2007 National Strategy for Natural Disaster Prevention, Response and Mitigation to 2020, and the 2008 National Target Program in response to Climate Change. According to these policies, the Ministry of Health is a member of the Central Steering Committee for Flood and Storm Control (chaired by the Ministry of Agriculture and Rural Development) and has the main responsibility in prevention and dealing with climate change-related health issues.

At the provincial, district, and commune levels, committees for Prevention and Control of Floods and Storms (with the health sector as a member) are directed by the People's Committee of the same level. Each year, each involved sector (including the health sector) develops a plan for prevention, control, and response to consequences of floods and storms.

At the local level, district emergency plans for storms and floods (responsibility of the district Agriculture and Rural Development Bureau) were reviewed and updated annually. However, the plans focused largely on disaster response rather than prevention. The plans lacked clear information on the coordination mechanism among different participating sectors and organizations. Plans did not clearly define the role of primary healthcare in implementation of the health emergency plan, and did not generally address the needs of vulnerable groups or gender considerations. Budgets for health emergency plans were also missing. Every facility reported that they lacked an emergency plan and that they were passive in responding to disaster problems. Health facilities did not have specific job descriptions for handling storm and flood-related health problems within each organization.

Most respondents (84.3%) reported that they knew of some general policy documents on prevention, control, and response to floods and storms. However, they did not know of any policy pertaining to roles and specific tasks of the primary healthcare system to respond to climate change and disaster-related health issues. Sixty-eight percent stated that legal and policy framework to support primary healthcare system response to storm and flood-related health problems have been inadequate.

Social mobilizations for responding to storm and flood-related health problems were good. Apart from health sector efforts, a number of climate change and disaster management projects funded by international donors or non-governmental organizations (e.g. The Red Cross, CARE, and ADB) had been implemented. The projects made substantial contributions to improving public awareness on climate change, disasters, and associated issues including health problems. Other sectors such as agriculture and rural development, the hydrometeorology unit, the radio station, and community organizations were also active in health promotion events and disseminating health promotion messages to local communities. All of the respondents said that to be more effective, better intersectoral coordination was needed.

### Healthcare finance

Healthcare finance findings were extracted from questionnaires and in-depth interviews. Questions were asked about availability of separate funding sources for storm and flood-related health problem prevention and treatment activities. These included questions on how to allocate funds for these activities, special mechanisms for patients derived from the health sector, sufficiency of funding for annual operations, availability of financial support from localities, how this financial support affected local residents and health facilities, and advantages and disadvantages of the financial policies at the health facility as related to implementation of prevention and treatment activities relating to storms and floods.

At the local level, activities of the Committees for Prevention and Control of Floods and Storms were financed from the National Reserve budget, state contingency budget, and local reserve funds for prevention and control of storms and floods. Local citizens aged 18–60 years and companies or agencies located in the community are mandated to make financial contributions to the local reserve fund. However, the budget amounts for prevention and control of storm and flood activities were limited and had no specific items for healthcare activities.

Except for the PhuVang District Health Bureau, the agency belonged to and was funded by the District People's Committee. PhuVang district and the commune health organizations received funding from the Thua Thien-Hue Provincial Health Bureau (a government budget) to pay staff salaries and other recurrent expenditures (i.e. electricity, water, meetings, travel). Despite playing important roles in health promotion activities and emergency care during storm and flood seasons, village health workers received little remuneration from the district health center (USD10–20 per person per month).

The health facilities reported that they received no additional budget from the health system for prevention and treatment of climate change and disaster-related health issues. In the event of a natural disaster, the PhuVang district health center had to seek financial support from the District People's Committee and/or the Thua Thien-Hue Provincial Health Bureau. Commune health center could also ask for support from the Commune People's Committee. In-depth interviews with the representatives from health facilities revealed that these extra amounts of money were normally small and used to cover small health staff allowances incurred while providing emergency services (e.g. instant food for patients, disinfection chemicals). Procedures to acquire this financial support were typically time-consuming.

The PhuVang district received some in-kind support from Thua Thien-Hue Pharmaceutical Company (i.e. medicine, medical equipment). The district also had projects on climate change and disaster management that were funded by international donors. Such funding was used to deploy specific activities such as capacity building, development of early-warning systems, or purchasing equipment. Again, the international funding allocated for implementing primary healthcare services was very limited.

In PhuVang district, 2011 health insurance coverage was nearly 70%. Funding from the health insurance fund was used to finance almost all health services for storm and flood-related health problems.

### Human resources

Human resources results were drawn from questionnaires and in-depth interviews. Demographic information on health staff was collected. The primary questions were about the number staff and the frequency of participation in training courses on prevention and treatment activities on storm and flood-related health problems during the past 5 years, the skills and knowledge needed to implement those activities effectively, assessment of the quantity and quality of the health workforce at their health facility, and opinions on the advantages and disadvantages in implementation of these prevention and treatment activities.

As of 2012, the PhuVang healthcare system had 258 employees: 147 worked at the district health level (102 in the district hospital, 41 in the district health center, four in the district health bureau), and 111 worked in the 20 commune health centers. The district hospital had two doctors with specialization level 1 on emergency care, and four doctors with specialization level 1 on surgical operations (with one of the four specialized in injury and trauma). The district health center had one master of public health and seven doctors of specialization level 1 in preventive medicine and public health. Each of the 20 commune health centers had at least one medical doctor. The number of commune health center staff was 3.4 per 1,000 populations. In addition, there were 369 village health workers supporting commune health center's health activities. The PhuVang healthcare system did not have any environmental health specialists.

During the last 3 years, all PhuVang professional healthcare staff received at least one training session on a topic relevant to prevention and control of climate change and disaster-related health issues. The training sessions included topics such as underwater rescue, first-aid, and transporting victims. Preventive medicine staff took part in the training on raising public awareness of the hazardous impacts of storms and floods, and communicable disease surveillance and control. Clinical staff attended training on emergency care, diagnosis, management, and treatment of injuries of injury, drowning, and snake bites. However, the quantity and topics covered by the training sessions were inadequate. Eighty-six percent of respondents reported that the number of training sessions did not meet actual needs. Seventy-nine percent stated that they needed training on specialized skills such as disaster planning, development and management of emergency plans, and effective referral of patients during storm and flood seasons.

### Medical products and technology

Medical product and technology was assessed with questions that asked for a list of a basic unit of medicine, medical devices, medical equipment for mobile emergency teams, availability of related medicines, availability of medical equipment, availability of treatment protocols, and advantages and disadvantages of these medicines and medical equipment for storm and flood-related health problems.

Lists of essential medicines and medical equipment were well described in the 2012 Thua Thien-Hue Provincial Health Bureau plan for storm and flood prevention and control. These essential medicines and medical equipment for emergencies were available in all of the facilities. Seventy-eight percent of respondents reported that their health facilities always had sufficient essential medicines for treatment of storm and flood-related health issues. Twenty-two percent reported that their facilities experienced temporary shortages of some medicines and supplies during some storm and flood days. First-aid kits were available at each health facility. Ninety-three percent of participants thought they had enough medical equipment for first-aid services during storms and floods. Only 7% thought that they needed more kits to distribute to each of the district village health workers.

The health facilities reported that they had protocols for drowning rescue, electrical shock, and first-aid for injuries, but our field visits revealed that only half (three out of six) had such protocols. None of the six facilities had a specific emergency plan.

### Health information system

The results for health information systems were extracted from the questionnaires. Health staff were asked how they stored information and reports on prevention and treatment activities, how they informed local inhabitants, whether there was a separate report on prevention and treatment activities for storm and flood-related health problems, how they cooperated with the meteorological agency, and advantages and disadvantages of data management and reporting.

Consistent with the health sector reporting system of Vietnam, the PhuVang commune health centers sent annual reports to the district health center. The district health center and district hospital submitted annual reports to the Thua Thien-Hue Provincial Health Bureau. The Thua Thien-Hue Provincial Health Bureau then sent provincial annual reports to the Ministry of Health.

During storm and flood seasons, the commune health center submitted daily health reports to the district health center and Commune People's Committee. The district health center subsequently made an overall district report for submission to the provincial health department and District People's Committee. These reports were in paper formats and not stored well. Other vertical programs also required the commune health centers and district health center to complete several reports and forms. At the district hospital, patient records were not organized in a way that facilitated patient management. System information on patient referrals and back-referrals were usually missing. The qualitative study found that data that could be used for planning and management (including population and epidemiological data) were largely lacking.

A vulnerability assessment, a method to identify hazards and determine their possible effects on a community, activity or organization, was not done in the PhuVang district. An early-warning system was not officially developed, and local residents were dependent on disaster announcements through mass media and a community loud speaker system.

## Discussion

This section discusses six building blocks of primary health system in PhuVang district, Thua Thien-Hue province, in terms of responding to storm and flood-related health problems. Functional characteristics of a primary health system, including availability, affordability, accessibility, and quality according to WHO recommendation are addressed.

### Service delivery

Availability of health services is an important aspect of a well-functioning primary healthcare system (27, 28). Disease surveillance and outbreak confirmation activities that support preventive medicine services were not well-implemented in the PhuVang district. Population-based health promotion campaigns about storms and flood-related issues had been regularly implemented. As recommended by the WHO, close disease surveillance systems should be in place and functional. Surveillance acts as an early-warning system for infectious disease outbreaks, provides information for identifying known and previously unknown non-infectious health hazards, and ensures that health services address the needs of the population and vulnerable groups (29).

In line with previous studies (1, 2, 4–10), our study showed that there was a relationship between storms and floods and the number of disease or illness cases (including injuries) in the study area. During storm and flood events, accidents or emergencies were the most common reasons for people to seek health care. However, medical emergency services, especially surgical operations and referral systems, were not always available during storm and flood seasons due to building damage, electricity outages, or inadequate professional staff. Similar situations were found in rural Ethiopia in 2007 (30) and India in 2008 (31). According to the WHO, timely and effective emergency services could save many lives during disaster events, and health facilities should prepare for changes in the populations who attend outpatient clinics because floods might cause population displacement (32). Therefore, dysfunction of emergency services in the primary health system should be actively prevented. Existing guidelines for dealing with disaster-related health problems such as the WHO emergency response framework (32) and the SPHERE project standards of care in mass casualty events (33) should be referenced. Alternate sources for electricity, emergency transportation, and additional health professional availability are necessary during storm and flood seasons (28, 34).

### Policy and governance

National policies for responding to storm and flood-related health problems were available but needed to be strengthened and developed in order to transfer into action in local rural communities. Our study showed that district emergency plans were available. However, these plans focused largely on disaster response rather than prevention. The plans did not generally address the needs of vulnerable groups or have gender considerations. The plans did not clearly define the role of primary healthcare and had no clear information on coordination among different sectors and organizations. As a result, they did not have a clear working plan to prepare for or prevent climate hazard consequences. They were passive in responding to disaster problems. For example, to implement one prevention activity such as chemical spraying, health staff had to approach households and public areas with other stakeholders. However, those representatives lacked enthusiasm, which sometimes resulted in delayed or ineffective chemical spraying. In other words, there needs to be a working mechanism among participating stakeholders to effectively implement treatment and prevention activities. Because there was no clear cooperation with stakeholders, and passivity, the health staff did not have specific job descriptions within their organizations for handling storm and flood-related health problems.

Similar situations were reported in Mozambique, where emergency response plans for storm and flood-related health problems were not clearly described (35). A study from rural India reported a lack of clear coordination between the state and district levels, and between district and block and health center levels (31). Successful emergency plans depend on the collaboration of multiple agencies. Emergency plans for health care should have a clear role in different sectors, as well as multisectoral coordination (36–42). Ideally, emergency response should be led and coordinated by a body at the central and local levels and include all relevant health sector disciplines to address all potential health risks. More effort is needed for specific consideration of gender and vulnerable groups such as children, women, and the elderly (43).

### Healthcare financing

An important aspect of a good financial mechanism for addressing storm and flood problems is to prepare for surge capacity and stockpile resources (34). To avoid storm and flood-related health problems, a good health financing system is also needed to ensure better access to services needed by patients, and the safety of health facilities and equipment (44). The PhuVang district budget for prevention and control of storm and flood-related activities was limited and lacked specific items for healthcare activities. Little additional funding was available, and the procedures to procure the funding were time-consuming. A study from Mozambique found that the governmental budget for emergency response was critical, but procedures to access funds needed simplification to improve financial response (45). Another study from India also reported that existing resources were inadequate during flood events (31). Funding for flood response should be calculated on past experiences, and each facility should be granted emergency contingency funds in their annual budget (31, 46).

### Human resources

Human resources are a key challenge during disasters, and the problem is more pronounced in rural settings (47). Medical rescue teams were established in PhuVang district, but there were no epidemiologists or environmental health specialists to address epidemiological issues. For continuity of essential services and to minimize risks of potential communicable disease outbreaks through prevention, additional health staff with appropriate knowledge, competencies, and skills are important to provide support to ‘fatigued’ health officers. In 2012, the PhuVang district population in Thua Thien-Hue province was 178,103 persons (48). If health staff totaled 258 persons, the ratio of health staff to population would be 14/10,000. This ratio is significantly lower than the 23/10,000 persons suggested by WHO as necessary to reach related millennium development goals (MDGs) [fewer than 23/10,000 generally fails to achieve adequate coverage rates for selected primary healthcare intervention (49)].

Training staff in management of mass casualty incidents holds the key to effective and optimum use of available resources (50). However, in the PhuVang district, training on prevention and control of climate change and disaster-related health issues did not meet actual needs. Health staff skills relevant to storm and flood-related health problems were inadequate. Similar results were found in rural India (31) and Mozambique (50). Primary healthcare staff need to be provided with training topics such as dealing with floods, analysis of flood effects, scenario planning based on information from situation management (from the worst to the best), and monitoring of disease situations (51). A comprehensive training and education needs’ assessment is important for identification of the skills required for performance of specific health-related tasks in crisis preparedness and response.

### Medical products and technology

As stated in the 1993 Ordinance on Prevention and Control of Floods and Storms, the health sector is responsible for building reserves of medication and medical equipment, instructing and disseminating the use of emergency techniques, and prevention techniques for epidemics and diseases likely to occur after storms and floods. Healthcare organizations at all levels have a responsibility to fulfill this regulation.

Health facilities have to access standard treatment protocols to ensure uniformity of treatment for populations affected by storms and floods. However, our study revealed that emergency treatment protocols were not available in all health facilities. In the absence of Ministry of Health-guided rescue protocols, WHO treatment protocols or Medicine Sans Frontiers guidelines could be used (31).

The WHO recommends the Interagency Emergency Health Kit (containing essential medicines and medical devices for primary healthcare workers with limited training) which contains oral and topical medicines to be used and tailored to reflect local availability of medicines, devices, and common flood types (27, 52). Reviewing the inventory list of stocked medicines and equipment, together with the essential medicine and device list, would show whether supplies match disaster response needs, as well as the location and accessibility of these supplies (53). More kits might need to be distributed to each village health worker in the district.

### Health information system

An important issue for effective disaster response was having the information to make a disaster response plan. A plan for a quick response includes the medical relief activities, requires access to health services data, and facilities during the pre-disaster phase. In the PhuVang district, data useful for planning and management (including population and epidemiological data) were largely lacking. A study from Indonesia showed that lack of necessary information or a prolonged deficiency of information meant that aid agencies were unable to efficiently distribute relief and provide assistance (54). Another study from Mozambique showed a similar situation (55). Introduction of a computerized health information system in Uganda resulted in health workers putting greater value on generated data, and accurate access to information was extremely important (56).

The PhuVang district lacked a disease early-warning system (DEWS) based on epidemic surveillance. DEWS plays an important role in overseeing the risks and signs needed for a timely response to future flood disasters (57). It remains a big challenge to assure that everyone receives timely warnings, understands the warnings, and can potentially take prompt action (55). Pakistan experienced an extreme flooding in 2010 that affected approximately 18 million persons. In response to the emergency, the Pakistan Ministry of Health and WHO enhanced existing DEWS for outbreak detection and response. Those improvements in DEWS increased system usefulness in subsequent emergencies. An effective community-based early-warning and evacuation system, including cyclone shelters for evacuation, was a crucial factor in saving many lives (58).

### Limitations of the study

Epidemiological studies of the impact of storms and floods, identifying risk groups, and delays in understanding the full extent of disease outcomes (acute and chronic) need further investigation. This study was not able to include such studies, but they are necessary to achieve the full potential of secondary or tertiary prevention strategies in the healthcare sector.

## Conclusion

Primary care system capacity in rural Vietnam is inadequate for responding to preventive and treatment healthcare for storm and flood-related health problems. Developing clear facility preparedness plans with detailed standard operating procedures during floods and identifying specific job descriptions will strengthen the future response to floods. Each facility should have contingency funds available for emergency response. Health facilities should ensure that Ministry of Health emergency treatment protocols for healthcare delivery are available. Introduction of a computerized health information system resulted in health workers putting greater value on generated data and should be used to speed up information and data processing. National and local policies need to be strengthened and developed so that they can be translated into action in rural communities.

## References

[1] Thornes JE, McCarthy JJ, Canziani OF, Leary NA, Dokken DJ, White KS (2001). IPCC, 2001: Climate change 2001: impacts, adaptation and vulnerability, Contribution of Working Group II to the Third Assessment Report of the Intergovernmental Panel on Climate Change.

[2] Noji EK (1991). Natural disasters. Crit Care Clin.

[3] Field CB (2012). Managing the risks of extreme events and disasters to advance climate change adaptation: special report of the intergovernmental panel on climate change.

[4] Ohl CA, Tapsell S (2000). Flooding and human health. BMJ.

[5] Howard MJ, Brillman JC, Burkle FM (1996). Infectious disease emergencies in disasters. Emerg Med Clin North Am.

[6] Abrahams MJ, Price J, Whitlock FA, Williams G (1976). The Brisbane floods, January 1974: their impact on health. Med J Aust.

[7] Kunii O, Nakamura S, Abdur R, Wakai S (2002). The impact on health and risk factors of the diarrhoea epidemics in the 1998 Bangladesh floods. Public Health.

[8] Kshirsagar NA, Shinde RR, Mehta S (2006). Floods in Mumbai: impact of public health service by hospital staff and medical students. J Postgrad Med.

[9] Wind TR, Joshi PC, Kleber RJ, Komproe IH (2013). The impact of recurrent disasters on mental health: a study on seasonal floods in northern India. Prehosp Disaster Med.

[10] WHO (2002). Floods: climate change and adaptation strategies for human health.

[11] World Bank (2010). Vietnam – Weathering the storm: options for disaster risk financing in Vietnam.

[12] Bich TH, Quang LN, Ha LTT, Hanh TT, Guha-Sapir D (2011). Impacts of flood on health: epidemiologic evidence from Hanoi, Vietnam. Glob Health Action.

[13] Vietnamese Government (2008). National target program in response to climate change.

[14] Ministry of Health Vietnam, Health Partnership Group (2008). Joint annual health review 2007.

[15] Ministry of Health Vietnam, Health Partnership Group (2010). Joint annual health review 2010: Vietnam's health system on the threshold of the five-year plan 2011–2015.

[16] Staber U, Sydow (2002). Organizational adaptive capacity: a structuration perspective. J Manag Inq.

[17] van den Berg B, Brouwer WB, Koopmanschap MA (2004). Economic valuation of informal care. An overview of methods and applications. Eur J Health Econ.

[18] Chakravarthy BS (1982). Adaptation: a promising metaphor for strategic management. Acad Manag Rev.

[19] Olmos S (2001). Vulnerability and adaptation to climate change: concepts, issues, assessment methods.

[20] Policymakers Sf, IPCC (2007). Climate Change 2007: Impacts, adaptation and vulnerability.

[21] Turner BL, Kasperson RE, Matson PA, McCarthy JJ, Corell RW, Christensen L (2003). A framework for vulnerability analysis in sustainability science. Proc Natl Acad Sci U S A.

[22] Gallopín GC, Funtowicz S, O'Connor M, Ravetz J (2001). Science for the twenty-first century: from social contract to the scientific core. Int Soc Sci J.

[23] Smit B, Wandel J (2006). Adaptation, adaptive capacity and vulnerability. Global Environ Change.

[24] Jones L, Ludi E, Levine S (2010). Towards a characterisation of adaptive capacity: a framework for analysing adaptive capacity at the local level.

[25] WHO (2007). Everybody's business: strengthening health systems to improve health outcomes WHO's framework for action.

[26] Ahuvia A (2001). Traditional, interpretive, and reception based content analyses: improving the ability of content analysis to address issues of pragmatic and theoretical concern. Soc Indic Res.

[27] PAHO (2000). Natural disasters: protecting the public's health.

[28] Axelrod C, Killam PP, Gaston MH, Stinson N (1994). Primary health care and the Midwest flood disaster. Public Health Rep.

[29] WHO (2005). Communicable disease control in emergencies: a field manual.

[30] Abaya SW, Mandere N, Ewald G (2009). Floods and health in Gambella region, Ethiopia: a qualitative assessment of the strengths and weaknesses of coping mechanisms. Glob Health Action.

[31] (2000). The Johns Hopkins and the International Federation of Red Cross and Red Crescent Societies. Emergency health services.

[32] WHO (2013). Emergency response framework (ERF).

[33] SPHERE (2011). Humanitarian charter and minimum standards in humanitarian response. http://www.sphereproject.org/content/view/720/200/lang,english/.

[34] Phalkey R, Dash SR, Mukhopadhyay A, Runge-Ranzinger S, Marx M (2012). Prepared to react? Assessing the functional capacity of the primary health care system in rural Orissa, India to respond to the devastating flood of September 2008. Glob Health Action.

[35] MOH (2008). Health cluster report: January–June 2008.

[36] Kovats RS (2000). El Niño and human health, in Bulletin of the World Health Organization.

[37] WHO (2005). Using climate to predict infectious disease outbreaks: a review communicable.

[38] WHO (2007). Risk reduction and emergency preparedness. WHO six-year strategy for the health sector and community capacity development.

[39] Kaji AH, Lewis RJ (2006). Hospital disaster preparedness in Los Angeles County. Acad Emerg Med.

[40] Dar OA, Khan MS, Murray V (2011). Conducting rapid health needs assessments in the cluster era: experience from the Pakistan flood. Prehosp Disaster Med.

[41] UNDP (2012). Governance and capacity building in post-crisis Aceh. A Report by Australian National University Enterprise Disclaimer.

[42] (2012). Thua Thien Hue People's Committee: Assessment of prevention of flood, storm and natural calamities in 2011. Orientation, mission of prevention of flood, storm and natural calamities in 2012.

[43] WHO (2009). Strengthening WHO's institutional capacity for humanitarian health action.

[44] Ministry of Health of the Republic of Macedonia (2009). Crisis preparedness planning for the health system in the Republic of Macedonia.

[45] Hellmuth ME, Moorhead A, Thomson MC, Williams J Climate risk management in Africa: learning from practice.

[46] Hanfling D (2006). Equipment, supplies, and pharmaceuticals: how much might it cost to achieve basic surge capacity?. Acad Emerg Med.

[47] Neil R (2003). A call for help. Collaboration with community officials is key. Mater Manag Health Care.

[48] Phu Vang District Thua Thien Hue province. http://www3.thuathienhue.gov.vn/GeographyBook/?sel=3&id=741.

[49] WHO (2010). Monitoring the building blocks of health systems: a handbook of indicators and their measurement strategies.

[50] (2002). Abt Associates, USAID. Mozambique 1999–2000 Floods Impact Evaluation: Resettlement Grant Activity. Emergency Recovery: Agriculture and Commercial Trade (ER: ACT) U. S. Agency for International Development (USAID).

[51] WHO (2009). Response to the 2009 floods emergency in Namibia Preventing diseases, saving lives.

[52] WHO (2010). Natural disasters: protecting the public's health.

[53] WHO (2006). The Interagency Emergency Health Kit 2006. Medicines and medical devices for 10,000 people for approximately 3 months. An interagency document.

[54] Alexander D (2006). World Disasters Report 2005: focus on information in disasters. Disasters.

[55] SARCOF (2002). Flood management in Mozambique.

[56] Fenenga C, de Jager A (2007). Health management information systems as a tool for organizational development.

[57] OCHA (2008). Uganda floods: lesson learn workshop. Final report.

[58] CDC (2012). Early warning disease surveillance after a flood emergency–Pakistan, 2010. MMWR Morb Mortal Wkly Rep.

# Sub article: Epidemiology of dengue fever in Hanoi from 2002 to 2010 and its meteorological determinants

**DOI:** 10.3402/gha.v7.23074

**Published:** 2014-12-08

**Authors:** Dao Thi Minh An, Joacim Rocklöv

**Affiliations:** 1 Department of Epidemiology, Institute for Preventive Medicine and Public Health, Hanoi Medical University, Hanoi, Vietnam; 2 Epidemiology and Global Health, Department of Public Health and Clinical Medicine, Umeå University, Umeå, Sweden

**Keywords:** dengue fever, epidemiology, time-series analysis, weather, climate

## Abstract

**Background:**

Dengue fever (DF) is a growing public health problem in Vietnam. The disease burden in Vietnam has been increasing for decades. In Hanoi, in contrast to many other regions, extrinsic drivers such as weather have not been proved to be predictive of disease frequency, which limits the usefulness of such factors in an early warning system.

**Aims:**

The purpose of this research was to review the epidemiology of DF transmission and investigate the role of weather factors contributing to occurrence of DF cases.

**Methods:**

Monthly data from Hanoi (2002–2010) were used to test the proposed model. Descriptive time-series analysis was conducted. Stepwise multivariate linear regression analysis assuming a negative binomial distribution was established through several models. The predictors used were lags of 1–3 months previous observations of mean rainfall, mean temperature, DF cases, and their interactions.

**Results:**

Descriptive analysis showed that DF occurred annually and seasonally with an increasing time trend in Hanoi. The annual low occurred from December to March followed by a gradual increase from April to July with a peak in September, October. The amplitude of the annual peak varied between years. Statistically significant relationships were estimated at lag 1–3 with rainfall, autocorrelation, and their interaction while temperature was estimated as influential at lag 3 only. For these relationships, the final model determined a correlation of 92% between predicted number of dengue cases and the observed dengue disease frequencies.

**Conclusions:**

Although the model performance was good, the findings suggest that other forces related to urbanization, density of population, globalization with increasing transport of people and goods, herd immunity, government vector control capacity, and changes in serotypes are also likely influencing the transmission of DF. Additional research taking into account all of these factors besides climatic factors is needed to help developing and developed countries find the right intervention for controlling DF epidemics, and to set up early warning systems with high sensitivity and specificity. Immediate action to control DF outbreak in Hanoi should include an information, communication, and education program that focuses on training Hanoi residents to more efficiently eliminate stagnant puddles and water containers after each rainfall to limit the vector population growth.

The incidence of dengue fever (DF) has grown dramatically around the world in recent decades. Over 2.5 billion people – over 40% of the world's population – are now at risk. WHO currently estimates there may be 50–100 million dengue infections worldwide every year (1). The Intergovernmental Panel of Climate Change (IPCC) warned that up until 2080, there may be 1.5–3.5 billion people worldwide who have to face the risk of DF infection due to climate change and the effects of the earth warming (2). New estimates show this may be substantially underestimated if economic development was less positive (3, 4). DF appeared in Vietnam in the late 1950s. Since then, DF became endemic with seasonal peaks occurring yearly and with a repeating epidemic pattern ranging from 4 to 10 years (peaks in 1983, 1987, and 1998). The milestone of DF epidemics in Vietnam was the large-scale outbreak in 1998 that impacted 57 out of total 61 provinces with the number of infected patients reaching 234,920 including 377 deaths. In response to this crisis, the Vietnam Government has approved the national dengue prevention program with the regions. The northern dengue control program, with its head office located in the National Institute of Hygiene and Epidemiology (NIHE), was established and started in 1999 (March/1999) (5). Since then, Vietnam appears to have controlled DF outbreaks for a long period; however, in 2009, the country once again experienced a DF outbreak in which DF cases peaked at 74,000 cases in October 2009 (increased by 17% compared with the same period in 2008) including 58 reported deaths (6).

Hanoi, one of the two biggest cities in Vietnam, experienced 16,263 DF cases in 2009 that spread to all of Hanoi's districts and occupied 87% total DF cases in the northern area. The number of DF cases was 6.7 times compared with the number in 2008 in Hanoi. The Ministry of Health noticed that the outbreak in Hanoi in 2009 was the most severe outbreak during the past 10 years with 121 cases per 100,000 population (7).

According to the Ministry of Health's statistics, the years that Vietnam experienced huge DF outbreaks during the past 10 years (1987, 1998, 2009) coincided with years of increased El Nino and La Nina activity (8). Recently, studies have been published on DF hot spots and the disease dynamics dispersion of DF over the period 2004–2009 in Hanoi, Vietnam, and one study quantifying the Emergence of Dengue in Hanoi, Vietnam: 1998–2009 (9, 10). However, studies to understand how much weather factors influenced the DF epidemic, especially in Hanoi, are scarce. Such studies are important to provide useful evidence for DF control programs through the development of early warnings (11). Therefore, the purpose of this study was to investigate characteristics of DF cases in Hanoi in relation to variation of weather factors over the period 2002**–**2010.

## Materials and methods

### Study area

Hanoi is the capital of Vietnam, ranked second among the country's most populous cities. It has been the most important cultural and political center with a population estimated at around 3 million spread over nine inner and five outer districts in 2008. On 1 August 2008, one further inner and 14 outer districts merged with the metropolitan area of Hanoi which increased Hanoi's total area to 334,470 hectares in 29 subdivisions with the new population reaching 6,232,940, effectively tripling in size. Hanoi's transportation density of people and goods remained second of the nation.

In 2008, Hanoi experienced heavy rain and floods (12). In general, Hanoi is characterized by a warm humid subtropical climate with plentiful precipitation peaking in the summer season and averaging 114 rainfall days per year in modern times. The city experiences a typical climate of northern Vietnam with two separated seasons: hot and humid summers, and relative to other parts of the nation cold and dry winters. Summers, lasting from May to September, are hot and humid with an average temperature of 28.1°C, receiving the majority of the annual rainfall. The winters, lasting from November to March, are relatively mild, dry (in the first half) or humid (in the second half) with an average temperature of 18.6°C. Hanoi also has two transition periods in April and October, that is, spring and fall. The temperature variation width ranges from 8 to 37°C (13).

### Data collection

DF was categorized in group B of infectious diseases in which the Infectious Diseases Act in Vietnam stipulates it mandatory that DF must be notified within 24 hours of diagnosis by all medical clinics and laboratories (14). Circulars of guidance on notification, communication, and reports of infectious diseases regulates that in each province/city, DF cases must be reported *weekly* by the commune health centers and the district hospitals to district health centers which reports to the department of preventive medicine at provincial level (PDPM) using WHO 2009 criteria of DF definition (Annex 1). The weekly reported DF cases were then collapsed into monthly aggregated numbers by PDPM and reported to the NIHE (15). We extracted monthly aggregated DF data reported by 14 old districts of Hanoi to the Hanoi PDPM from 2002 to 2010. We also obtained daily temperatures in centigrade, relative humidity in percentages and rainfall in millimeters for 2002–2010, reported by Lang center (the Hanoi Centre of Hydrometeorology before 2008). Weather data were collapsed into monthly mean values. The monthly aggregated DF data were merged with the monthly mean weather data for the epidemiological time-series analysis.

### Statistical methods

A dengue outbreak is characterized by the occurrence of excess DF cases compared to what would normally be expected in a defined community, geographical area or season. Characteristics of DF epidemic from 2002 to 2010 were described and tested by including variables for the estimation of time trends. Variability of temperature, rainfall, humidity and DF cases were hypothesized to precede the upsurge or decay of DF cases with a lag of up to 6 months. Population changes were adjusted for in the denominator of the dengue count series (offset). Spearman correlation was estimated between DF cases and each five weather factors to identify the most influential preceding months (lag times) that influenced the occurrence of DF cases. The lag variables with correlations running from ∣0.3∣ to ∣1∣ were selected to be independent variables in a subsequent negative binomial regression model. Bonferroni corrections per number of lag tests were conducted to adjust for multiple testing with an adjusted significance level at 0.05/6 (16). The negative binomial regression model was chosen to relax the assumption of mean and variance equality in the Poisson distribution of counts data. In 2009, Yang indicated that mortality rates of adult mosquitoes increase with increasing temperature above 30°C (17). Fouque and Dibo indicated that heavy rainfall can potentially flush away larvae or pupae or the immature stage of Aedes mosquitoes. Heavy rainfall can also increase the mortality rate of adult mosquitoes (18, 19). Therefore, a threshold of 30°C and 450 mm rainfall was used for running piecewise linear spline functions with the hypothesis that there was a positive linear relationship between DF cases when temperature increases from 15 to 30°C and rainfall increases from 0 to 450 mm. Beyond 30°C and 450 mm, these relationships would be in reverse order (negative). Lag variables that were statistically significant in a simple negative binomial regression model would then be included in a multiple regression model. A manual stepwise model selection approach with forward inclusion was used to identify the most appropriate model based on the Generalized Cross Validation (GCV) scores and Akaike Information Criterion (AIC). A time variable was also used as an independent variable to control trends of DF cases over time not explained by the other variables. Predictions from the established models and its relationship to the observed dengue cases were evaluated based on Root Mean Square Error (RMSE), and correlation. We also validated the fit of models by performing residual diagnoses, and graphic examination.

The fitted models can be expressed as follows:

a $$\log\left(DF_{t}\right)=D_{0}+D_{temp}+D_{rain}+D_{AR}+D_{trend}+offset\left(\text{log}(pop)\right)$$

*Where t* refers to the month of the observation; (*DF*_*t*_) denotes the observed monthly DF cases during month *t*.

Thus,

D_*temp*_=lags of monthly mean temperature

D_*rain*_=lags of monthly mean rainfall

D_*AR*_=lags of auto-regression (DF cases)

D_*trend*_=a function of time trend (year)

offset(log(pop))=the DF denominator adjustment of mid-year population.

In addition, penalized cubic spline functions were fit to further explore non-linear patterns in additional models taking the form:

$$\log\left(DF_{t}\right)=D_{0}+s\left(D_{temp},df\right)+s\left(D_{rain},df\right)+s\left(D_{humid},df\right)+s\left(D_{AR},df\right)+s\left(D_{trend},df\right)+s\left(offset(\log(pop)),df\right)$$

Where *s(.)* denotes a smooth function; *df* represent degrees of freedom that are penalized in the model fitting from a start value of 10; *D*_*temp*_ *, D*_*rain*_ *, D*_*humid*_ *, D*_*AR*_ are the mean monthly temperature, rainfall, humidity, and DF case, respectively. *D*_*trend*_ represents factors for year of study period, respectively.

For all statistical tests, two-tailed tests were considered statistically significant with a p-value less than 0.05. All data manipulation were done in STATA and statistical analyses were performed in STATA and using the R package ‘mgcv’ (The R Foundation for Statistical Computing, version 3.0.0).

## Results

During the study period from January 2002 to December 2010, there were 28,793 DF cases in which more than 75% of them were aged between 15 and 44 years. Male cases were higher at all years. DF cases occurred mostly in inner districts (72.07%) and the rest belonged to outer districts. Within inner districts, four bordering districts faced recurrent outbreaks over the 9 years. These were Dong Da, Thanh Xuan, Hoang Mai, Thanh Tri, Hai Ba Trung. Within the outer districts, the two bordering areas Thanh Tri and Tu Liem suffered the highest number of DF cases (Map 1). DF cases increased from 125 cases in 2002 to 649 cases in 2005, and after that, DF cases increased with greater magnitude and intensity with the, at the time, record of 2,707 cases in 2006 to become even worse in 2009 with 16,268 cases. The rate of DF cases per 100,000 population per year increased significantly from 2002 to 2010 (p-value of trend test is 0.03) and numbers of DF cases per month increased significantly over 108 months of 9 years (*p*-value of trend test is <0.000). The highest dengue cases in the study period were reported in September and October 2009 with 4,145 and 4,120 cases, respectively. DF outbreaks occurred in Hanoi from 2006 to 2010 with the number of cases being 4.3, 3.3, 4.1, 25.6, and 5.4 times higher, respectively, compared with previous years (Fig. 1).

**Fig. 1:** Annual distributions of DF cases in Hanoi (study period: 2002–2010).

This study indicated that in the period 2002–2010, DF cases generally occurred annually in a seasonal way, with the exception of 2002. The general pattern revealed a few DF cases that appeared sporadically from December of the previous year to March of the next year, increased gradually from April to July, peaked in September, October, and decreased quickly in November and December. A new circle of DF cases would occur the year after. It is easily observed graphically that before each peak of DF cases, there is always a preceding 1–2 months excessive rainfall (Fig. 2). As can be seen in Fig. 2, rainfall peaks in July and August, and then DF cases peak a few months later in September and October. In the period when rainfall was peaking, temperature were always around 20–30°C and humidity was around 70–80% (Fig. 2). Table 1 reveals that three weather factors are significantly correlated with DF cases on the basis of Spearman correlations. These are temperature, rainfall, and humidity. Temperature is significantly correlated with DF cases through lag 0–3, with the biggest correlation at lag 2 (*r*=0.53). Similarly, rainfall is significantly correlated with DF cases through lag 1–3 with the largest correlation at lag 2 (*r*=0.47). Humidity had only moderate correlation with DF cases at lag 0, while non-significant over the other lags times. Past DF cases were correlated with DF cases at the moment through lag 1–3, but with the highest correlation with *r*=0.84 at lag 1. There is a significantly strong positive correlations between DF cases and population with *r*=0.63. Quasi Poisson Regression showed that there is a significant and positive linear association between temperature and DF cases when temperature was below (≤) 30°C, but this association is reversed when temperature increased beyond 30°C. In contrast, there is only significantly positive linear regression between DF cases and rainfall when temperature was below (≤) 450 mm. Noticeably, only DF cases at lag 1 significantly precede risk of DF cases while temperature and rainfall preceded risks of DF cases by lag 1–3 (Table 1).

**Table 1:** Correlation and regression coefficients (negative binomial) between DF case and independent variables (bivariate Spearman rank correlations), Hanoi, 2002–2010

	Temperature			Rainfall			Case		Population		Humidity	
	
Lag time	Spearman's rho (p)	Spline (^o^C)	Quasi Poisson's coefficient (p)	Spearman's rho (p)	Spline (mm)	Quasi Poisson's coefficient (p)	Spearman's rho (p)	Quasi Poisson's coefficient(p)	Spearman's rho (p)	Quasi Poisson's coefficient (p)	Spearman's rho (p)	Quasi Poisson's coefficient (p)
Lag 0	0.28 (0)*	≤30	0.24 (0)*	0.14 (0.16)	na	na	na	na	0.63 (0)*		−0.35 (0)*	−.1209 (0.000)*
		>30	−0.25 (0.004)*		na	na	na			0.0007 (0)*		
Lag 1	0.47 (0)*	≤30	0.23 (0)*	0.36 (0)*	≤450	0.004 (0)*		0.002 (0.000)*	na			na
		>30	−1.94 (0.008)*		>450	−0.0008 (0.922)	0.84 (0)*			na	−0.10 (0.30)	
Lag 2	0.53 (0)*	≤30	0.22 (0)*	0.47 (0)*	≤450	0.006 (0)*		−0.001 (0.287)	na			na
		>30	−0.52 (0.447)		>450	−0.008 (0.21)	0.57 (0)*			na	0.077 (0.44)	
Lag 3	0.44 (0)*	≤30	0.15 (0)*	0.38 (0)*	≤450	0.006 (0)*						na
		>30	0.75 (0.362)		>450	−0.11 (0.104)	0.37 (0)*	0.00009 (0.884)	na	na	0.135 (0.17)	na
Lag 4	0.25 (0.01)*	na	na	na	na	na	0.27 (0.01)*		na	na	na	na
Lag 5	0.02 (0.81)	na	na	na	na	na	0.21 (0.03)*		na	na	na	na
Lag 6	−0.21 (0.03)	na	na	na	na	na	0.18 (0.07)		na	na	na	na

* s p≤0.05.

**Fig. 2:** Whole period and monthly mean distribution of DF cases, rainfall, temperature, and humidity, Hanoi, 2002–2010.

Four models were developed using the manual multiple forward stepwise regression analysis using lag 1–3 of predictors (Table 2). In the first step, we incorporated an annual time trend factor variable of the nine consecutive years (2002–2010), and developed model-1 considering rainfall and DF cases with the hypothesis that rainfall (lag 1–3) is a necessary condition for mosquito reproduction. In the second steps we put rainfall and temperature (lag 1–3) together in model-2 with the hypothesis that temperature may also play an important role in reproduction and proliferation of disease. The third step involved putting rainfall, temperature, and lag cases together in model-3 with the hypothesis that DF cases would contribute by an inbuilt momentum in the disease growth process after the onset of the epidemic. We also ran model-4 by putting rainfall, temperature, autocorrelation, and interaction among these three factors together in a model with the hypothesis that there would be an interaction among rainfall, temperature and DF cases that could make outbreak more explosive. We found that by different lag time (lag 1–3), model-1 demonstrated a capacity to explain a correlation of maximum 78.1% comparing the predictions of the model to the total variations in the occurrence of DF case, while correlations of model-2, model-3, and model-4 explained a Spearman correlation of the predicted values to the observed of maximum 85.1, 87.8 and 88%, respectively. Finally, a full model (model-5) including interactions between weather factors of lag 1–3 together with main effects was fitted to study more complex associations. In this model, the explanatory capability further increased over the previous four models and could explain a correlation of the predicted to the observed dengue cases to a correlation of 92%. We explored another model with an even higher lag period (>3); however, these models failed to add to explanatory capacity, and rather showed sharp declines in model fit. Hence, model-5 was chosen as the final model. While generating the model, all of the relevant assumptions were checked for assuring best possible model to be selected. For model-5, the AIC value equals 1,110.89 which is the lowest and similarly optimal value compared with those of model-4s from lag 1 to 3 running from 1,157, 1,157, and 1,183, respectively (Table 2). Moreover, its GCV score was the lowest compared with those of model-4 through lag 1–3 (45.2 against 58.9, 115, 171, respectively; see Table 2 and 3) while its RMSE score was the second compared with those of model-4 through lag 1–3 (33.63 against 205.95, 7.85, 1.25, respectively; see Table 2 and 3). Besides, graphs of the actual case against predicted values of model-4 at three lags of time and model-5 showed that model-5 performed the fitness of distribution of observed DF cases against predicted values (Fig. 3), and penalized spline function graphs of these exposure–response relationships are presented in Annex 2 to Annex 5, respectively.

**Fig. 3:** Predicted cases vs. observed DF cases in Hanoi, 2002–2010.

**Table 2:** Stepwise multivariate regression between DF cases and influent factors, Hanoi, 2002–2010

		Model-1				Model-2				Model-3				Model-4			
		
Lag		Coef	*p*	[95% Conf. interval]		Coef.	*p*	[95% Conf. interval]		Coef.	*p*	[95% Conf. interval]		Coef.	*p*	[95% Conf. Interval]	
	p and r correlation	*p*<0.001; *r*=72.6				*p*<0.001; *r*=81.9				*p*<0.001; *r*=87.8				*p*<0.001; *r*=88; AIC=1157; RMSE=205.95; GCV=58.9			
	rain1lag1	0.00	0.00	0.00	0.01	0.00	0.88	0.00	0.00	0.00	0.23	0.00	0.00	0.00	0.79	0.00	0.00
Lag1	temp1lag1					0.22	0.00	0.16	0.28	0.18	0.00	0.13	0.23	0.19	0.00	0.13	0.24
	lagcase1									0.00	0.00	0.00	0.00	0.00	0.00	0.00	0.00
	interact1													0.00	0.13	0.00	0.00
	p and r correlation	*p*<0.001; *r*=78.1				*p*<0.001; *r*=85.1				*p*<0.001; *r*=85.3				*p*<0.001; *r*=85.8 AIC=1157; RMSE=7.85; GCV=115.0			
	rain1lag2	0.01	0.00	0.00	0.01	0.00	0.01	0.00	0.00	0.00	0.00	0.00	0.00	0.00	0.06	0.00	0.00
Lag2	temp1lag2					0.17	0.00	0.11	0.22	0.15	0.00	0.10	0.21	0.16	0.00	0.11	0.21
	lagcase2									0.00	0.06	0.00	0.00	0.00	0.50	0.00	0.00
	interact2													0.00	0.01	0.00	0.00
	p and r correlation	*p*<0.001; *r*=74.0				*p*<0.001; *r*=75.2				*p*<0.001; *r*=75.8				*p*<0.001;*r*=76.4 AIC=1183; RMSE=1.29; GCV=171.0			
	rain1lag3	0.01	0.00	0.00	0.01	0.00	0.00	0.00	0.01	0.00	0.00	0.00	0.01	0.00	0.01	0.00	0.01
Lag3	temp1lag3					0.07	0.01	0.01	0.13	0.08	0.01	0.02	0.14	0.09	0.01	0.03	0.15
	lagcase3									0.00	0.11	0.00	0.00	0.00	0.01	0.00	0.00
	interact3													0.00	0.05	0.00	0.00

The full model also included an additional factor variable for month indicated that increasing monthly mean rainfall significantly preceded risks of increasing DF cases by 1–3 months, respectively (Table 3) while there was no significant association between temperature with DF cases except an inverse relationship by month 3. Numbers of DF cases of the two past months had significant autocorrelation with the number of DF cases of current month while numbers of DF cases at lag 3 had an inverse relationship with that of the current month. Interactions among rainfall, temperature, and DF cases at a lag time of 1–3 months always increase risk of increasing numbers of DF cases of the current month.

**Table 3:** Full multiple regression model coefficient and confidence intervals, Hanoi, 2002–2010

	*p*<0.001; *r*=92 AIC=1110; RMSE=33.63; GCV=45.2			
	
Model-5 Case	Coef.	P>z	95% CI	
_Iyear_2003	0.998	0.00	0.42	1.58
_Iyear_2004	1.013	0.00	0.45	1.57
_Iyear_2005	1.014	0.00	0.46	1.56
_Iyear_2006	2.176	0.00	1.62	2.74
_Iyear_2007	2.195	0.00	1.65	2.74
_Iyear_2008	1.323	0.00	0.77	1.87
_Iyear_2009	2.167	0.00	1.52	2.81
_Iyear_2010	2.277	0.00	1.68	2.87
temp1lag1	0.035	0.21	−0.02	0.09
rain1lag1	0.002	0.01	0.00	0.00
lagcase1	0.003	0.00	0.00	0.00
interact1	0.000	0.07	0.00	0.00
rain1lag2	0.003	0.00	0.00	0.01
temp1lag2	0.026	0.44	−0.04	0.09
lagcase2	0.001	0.03	0.00	0.00
interact2	0.000	0.00	0.00	0.00
rain1lag3	0.004	0.00	0.00	0.01
temp1lag3	−0.065	0.02	−0.12	−0.01
lagcase3	−0.001	0.04	0.00	0.00
interact3	0.000	0.00	0.00	0.00
_cons	−6.800	0.00	−8.42	−5.18

## Discussion

DF cases in Hanoi occurred annually and seasonally over the study period, 2003–2010, with recurrent peaks of DF cases in September and October. It also established a temporal relationship of recurrent patterns of rainfall and temperature preceding the outbreaks similar to Hii et al. (20, 11). In combination to autoregressive variables and incorporating lags in relationship up to 3 months, a correlation between observed and predicted dengue cases of above 90% was observed. This suggests a potential early warning system using these models with a lead time within this delay period (21).

Wilder-Smith and Schwartz from the Geo-Sentinel Surveillance Network had examined seasonality and annual trends for dengue cases among 522 returned travelers which indicated that dengue cases showed region-specific peaks for Southeast Asia (June, September), South Central Asia (October), South America (March), and the Caribbean (August, October) (22). DF has recently re-emerged globally with intensified epidemic and major epidemiological expansion since the 1980s, and has rapidly become a major epidemiological threat in Asia Pacific and South America (23). This current study also indicated that the number of DF cases increased significantly overtime. Hii et al. in their study of intensity and magnitude of dengue incidence in Singapore indicated that from 2000 to 2007, DF cases increased from 673 cases to the peak of 14,209 cases in 2005 (23). In Thailand, DF is on the rise; in 2012, Thailand recorded over 74,000 DF cases (24). Cambodia also observed an increasing trend of DF in which there were 15,597 cases between January and June in 2012 while that of 2011 was 4,604 cases, representing a 239% increase year-on-year (25). Worldwide there has been a 30-fold increase in cases of DF over the last 50 years (26).

Rainfall with stagnant water outdoors is considered a necessary condition for the breeding habitats of Aedes mosquitoes while temperature and humidity are, in combination, also a sufficient condition for effective development. Aedes mosquitoes adapt to harsh environmental conditions, which are sometimes produced by vector control programs or natural weather by laying their eggs in unusual outdoor habitats, or even on dry surfaces to wait up to several months for the appropriate amount of rainwater to hatch (16). Theoretical models of dengue transmission dynamics based on mosquito biology support the importance of temperature and precipitation in determining transmission patterns, but empirical evidence has been lacking. On a global scale, several studies have highlighted common climate characteristics of areas where transmission occurs (3). Meanwhile, longitudinal studies of empirical data have consistently shown that temperature and precipitation correlate with dengue transmission but have not demonstrated consistency with respect to their roles, and predictive performance with sufficient lead times. For example, cumulative monthly rainfall and mean temperature correlated positively with increased dengue transmission on the Andaman Sea side of Southern Thailand. On the Gulf of Thailand side, however, it was the number of rainy days (regardless of quantity) and minimum temperature that associated positively with incidence. Another study, farther north, in Sukhothai, Thailand, found that temperature had a negative effect on dengue transmission (27).

The current study indicated that there was a significant relationship between rainfall and DF cases. A graphic observation displayed that rainfall always peaks 2–3 months preceding the peaks of DF fever cases (Fig. 2) and model-5 showed that rainfall within 3 previous months at a level equal to or less than 450 mm was positively correlated with DF cases of the current month (Table 3). Our result is consistent with studies in Singapore and Brazil, which displayed that rainfall precedes risks of increasing DF cases by 1–5 months with higher risks being evident at 3–4 months (18, 19). Most recently, the outbreak of DF in Portugal which occurred during the unusually dry winter with rainfall predominantly in October through March then as of February 2013, resulted in over 2,000 cases among residents of Madeira in Portugal, most occurring between October and November 2012 (28). Hashizume et al. indicated that there was strong evidence for an increase in DF at high river levels during rainfall season. Hospitalizations increased by 6.9% for each 0.1 meter increase above a threshold (3.9 meters) for the average river level over lags of 0–5 weeks. Conversely, the number of hospitalizations increased by 29.6% (95% CI: 19.8, 40.2) for a 0.1 meter decrease below the same threshold of the average river level over lags of 0–19 weeks (29). This, once again, highlighted evidence of rainfall as a necessary condition for a DF outbreak explosion. Therefore, rainfall is still sensitively used as an indicator of a warning system for the DF outbreaks regarding stagnant water in natural puddles and canned food cover.

Assessment of risk of outbreak was mainly based on case, vector and virus surveillance which is already a part of the routine surveillance activity in many countries (30). The use of metrological data to predict and control dengue epidemics may not be a routine task for a health sector in many countries so far. Evidence of the relationship between rainfall and temperature from this study indicates that the integration of using climatic data into the existing surveillance activity may be beneficial to health workers working in the preventive medicine system in risk assessment and Information, Education and Communication (IEC) programs. In Hanoi, the IEC program to control DF outbreak was conducted in a way that if there was any outbreak of DF occurring in any district, then outbreak communication would be implemented to warn other districts following the IEC program delivered via loud speakers at health commune stations. Therefore, if using the integration approach, health workers should base levels of precipitations every month to make risk assessments along with looking at the number of cases and vectors measured from the surveillance system. Moreover, to prevent DF occurrence, the IEC program should be conducted in early April and last through October annually to remind people to destroy any stagnant puddles to eliminate breeding habitats of Aides mosquitoes after rainfall occurrence. This current study displayed that temperature precedes the risk of increasing DF cases by 1–2 months but this correlation was not statistically significant while an inverse correlation significantly happen at lag 3. The study conducted by Yan in Singapore also indicated that monthly mean temperature does not contribute to the prediction models of DF cases at any level (31).

However, temperature's role could be found to contribute to DF cases indirectly through interaction variables among rainfall, temperature, and DF cases which was significant at previous 2 months while there is a significantly inverse correlation between temperature and DF cases at lag 3. Studies in Singapore and Thailand showed that temperature precedes risks of increasing DF cases by 1–5 months and 6 months, respectively (23, 32).

In epidemiology, the infectious disease process chain of transmission always gives rise to autocorrelation. The autocorrelation arises as a natural feature of infectious disease systems as the number of new infections relates closely to the number of recent infections. A study by Joseph et al. in Puerto Rico (1988–1992) revealed a positive autocorrelation between the past and current DF (33). Hii et al.'s study in Singapore indicated that past DF cases from lag week 1 to 6 were considered to influence the occurrence of DF cases of the current week (34). Halide's study in city of Makassar indicated that the most important input variable in the prediction is the present number of DHF cases followed by the relative humidity 3–4 months previously (35). Autocorrelation also happened in the way of spatial autocorrelation in which geographical characteristics, density of population, and social factors contribute to the occurrence of DF cases in this area influence occurrence of DF cases in other areas especially in bordering areas. Suchithra indicated that there was a significant positive spatial autocorrelation of dengue incidence (36). This current study also indicated a partial correlation of numbers of DF cases significantly precede risks of increasing DF cases of the current month by 1–2 months. Moreover, this current study also showed that there were always DF outbreaks occurred in five bordering inner districts and two bordering outer districts every year where population density remains highest with lower social infrastructures. Mathuros in his study in Thailand implies that villages with geographical proximity shared a similar level of vulnerability to dengue (37).

**Map 1:** Distribution of DF cases in 14 districts of Hanoi from 2002 to 2010.

Overall, the implication of the full model (model-5) is that whenever there was rainfall, DF cases with the appropriate temperature ranging from 15 to 30°C, these three factors would interact together preceding risks of an explosion of DF cases by 1–2 months. Therefore, any time when there are sporadic DF cases and rainfall occurs in warm weather, a risk of DF outbreak should be taken into account. However, more sophisticated automated early warnings systems could potentially give better predictive power.

Studies demonstrated that the extents of contribution of climate factors to the occurrence of DF cases varied remarkably. A study by Nazmul Karim indicated that the model incorporating climatic data of two-lag months explained 61% of variation in the number of reported dengue cases (38). As our study revealed a correlation of 92% (model 5), we could support that the discriminate power of these variables are substantial. Hii et al.'s study in Singapore indicated that climate factors contributed 84% to the occurrence of DF cases. However, Suwich Thammapalo in his study on climate factors and DF cases indicated that variability in incidence was explained mostly (14.7–75.3%) by trend and cyclic change and much less (0.2–3.6%) by independent climatic factors (39). This current study displayed that rainfall, temperature, autocorrelation of DF cases and their interaction contributed 92% to the correlation between predicted and observed DF cases (model-5, the fitness model; see Table 3).

However, limitations of the study are that there are many influential factors of DF epidemiology including urbanization, density of population, and globalization with increasing transport of goods not controlled for. Similarly, the predictive ability of the models does not capture impacts of co-circulation of different dengue virus types and other virus changes causing more disease relating to immunity processes. In general, factors of herd immunity, government vector control capacity, and changes in serotypes contribute to dengue epidemics. However, likewise climate changes, drought, and flood are conditional factors for increasing Aedes population size (40). There is a further need for studies on modeling contributing factors to DF including not only climatic factors but also social demographic and economic factors as well as a program of vector control to supply, and virus monitoring, to establish more accurate DF prediction in the future.

## Conclusion

DF in Hanoi occurred annually and seasonally in the period 2002–2010 in which a couple of DF cases appeared sporadically from December of the previous year to March of the next year, then increased gradually from April to July and afterward sharply peaked in September, October then decreased quickly in November and December. A new circle of DF cases would occur the year after. Monthly mean rainfall, temperature, DF cases, and their interaction from lag 1 to lag 3 contributed up to 92% correlation of predicted and observed DF cases in Hanoi. Monthly mean rainfall, autocorrelation, and interaction were statistical significantly related with monthly DF cases at lag 1–3 while temperatures were significantly related at lag 3.

## Policy recommendation

- To establish a more accurate and comprehensive model of DF prediction and early warning, additional research taking into account other forces or factors related to the urbanization, density of population, globalization with increasing transport of people and goods, herd immunity, government vector control capacity, and changes in serotypes beside climatic factors is needed. However, the findings suggest that the predictive power of weather factors and autocorrelation process are high already without this information, and that timely notifications to control DF outbreak and support immediate action in Hanoi by an information, communication, and education program focusing on training Hanoi residents may be achievable on this basis

## Figures and Tables

**Annex 2:** Graphs of model-4/lag1.

**Annex 3:** Graphs of model-4/lag2.

**Annex 4:** Graphs of model-4/lag3.

**Annex 5:** Graphs of model-5.

## Appendix

Annex 1: Definition of DF case

*Dengue fever:* Presence of high and continuous fever from 2 to 7 days and two or more of the following retro-orbital or ocular pain, headache, rash, myalgia, arthralgia, leukopenia, or hemorrhagic manifestations (e.g. positive tourniquet test, petechiae; purpura/ecchymosis; epistaxis; gum bleeding; blood in vomitus, urine, or stool; or vaginal bleeding) but not meeting the case definition of dengue hemorrhagic fever. Anorexia, nausea, abdominal pain, and persistent vomiting may also occur but are not case-defining criteria for DF.

*Dengue hemorrhagic fever* is characterized by all of the following:

- Fever lasting 2–7 days
- Evidence of hemorrhagic manifestation or a positive tourniquet test
- Thrombocytopenia (≤100,000 cells per mm^3^)
- Evidence of plasma leakage shown by hemoconcentration (an increase in hematocrit ≥20% above average for age or a decrease in hematocrit ≥20% of baseline following fluid replacement therapy), or pleural effusion, or ascites or hypoproteinemia.

*Dengue shock syndrome* has all of criteria for DHF plus circulatory failure as evidenced by:

- Rapid and weak pulse and narrow pulse pressure (>20 mm Hg), or
- Age-specific hypotension and cold, clammy skin and restlessness.

*Laboratory criteria for diagnosis for case definitions*

1. Confirmatory
  - Seroconversion from negative for dengue-specific serum IgM antibody in an acute phase (≤5 days after symptom onset) specimen to positive for dengue-specific serum IgM antibodies in a convalescent-phase specimen collected ≥5 days after symptom onset, *or*
  - Demonstration of a ≥4-fold rise in reciprocal IgG antibody titer or hemagglutination inhibition titer to dengue antigens in paired acute and convalescent serum samples, *or*
2. Criteria for Epidemiologic Linkage
  - Travel to an dengue endemic country or presence at location with ongoing outbreak within previous 2 weeks of dengue-like illness, *OR*
  - Association in time and place with a confirmed or probable dengue case.

DF cases collected in this study included all cases of DF, dengue hemorrhagic fever, and dengue shock syndrome who have laboratory confirmation or meet the criteria for epidemiologic linkage.

## Appendix

Annex 2: Sensitivity analysis using non-linear cubic functions instead of linear for model 4 and 5. Relationships are shown per model, lag and variable.

## References

[1] World Health Organization (2013). Dengue and severe dengue. http://www.who.int/mediacentre/factsheets/en/.

[2] IPCC (2007). IPCC Fourth Assessment Report: Climate Change 2007. http://www.ipcc.ch/publications_and_data.

[3] Astrom C, Rocklov J, Hales S, Beguin A, Louis V, Sauerborn R (2012). Potential distribution of dengue fever under scenarios of climate change and economic development. EcoHealth.

[4] World Health Organization (2011). Regional Office for South East Asia. Comprehensive Guidelines for Prevention and control of dengue fever and dengue haemorrhagic fever.

[5] Website of the National Institute of Hygiene and Epidemiology. DHF Project implementation.

[6] World Health Organization, West Pacific Region, and W.r.o.i. Vietnam http://www.wpro.who.int/vietnam/topics/dengue/factsheet/en/index.html.

[7] Hung H.T (2012). Characteristics of dengue fever in Hanoi from 2002 to 2010 and some determinants.

[8] Website of the National Weather Service Cold and Warm Episodes by Seasons. http://www.cpc.ncepnoaa.gov/products/analysis_monitoring/ensostuff/esoyears.shtml.

[9] Toan do TT, Hu W, Quang Thai P, Hoat LN, Wright P, Martens P (2013). Hot spot detection and spatio-temporal dispersion of dengue fever in Hanoi, Vietnam. Glob Health Action.

[10] Cuong HQ, Hien NT, Duong TN, Phong TV, Cam NN, Farrar J (2011). Quantifying the emergence of dengue in Hanoi, Vietnam: 1998–2009. PLoS Negl Trop Dis.

[11] Hii YL, Zhu H, Ng N, Ng LC, Rocklöv J (2012). Forecast of dengue incidence using temperature and rainfall. PLoS Negl Trop Dis.

[12] Wikipedia (2008). Vietnam Floods. http://en.wikipedia.org/wiki/2008_Vietnam_floods.

[13] Climatetemps.com Climate of Hanoi, Vietnam average weather.

[14] Government of Vietnam (2007). Law 03/2007/QH12 of the national assembly of Vietnam: Law of prevention and control infectious diseases.

[15] Health M. O (2010). Circula 48/2010/TT-BYT. Guidance on declaration, communication, and reporting infectious diseases.

[16] Abdi H, Bonferroni, šidák, Salkind N (2006). corrections for multiple comparisons. Encyclopedia of Measurement and Statistics.

[17] Yang HM, Macoris MLG, Galvani KC, Andrighetti MTM, Wanderley DMV (2009). Assessing the effects of temperature on the population of Aedes aegypti, the vector of dengue. Epidemiol Infect.

[18] Fouque F, Carinci R, Gaborit P, Issaly J, Dominique JB, Sabatier P (2006). Aedes aegypti survival and dengue transmission patterns in French Guiana. J Vector Ecol.

[19] Dibo MR, Chierotti AP, Ferrari MS (2008). Study of the relationship between Aedes (Stegomyia) aegypti egg and adult densities, dengue fever and climate in Mirassol, state of Sao Paulo, Brazil. Mem Inst Oswaldo Cruz.

[20] Hii YL, Rocklov J, Ng N, Tang CS, Pang FY, Sauerborn R (2009). Climate variability and increase in intensity magnitude of dengue incidence in Singapore. Glob Health Action.

[21] Hii YL, Rocklöv J, Wall S, Ng LC, Tang CS, Ng N (2012). Optimal lead time for dengue forecast. PLoS Negl Trop Dis.

[22] Wilder-Smith AS, Schwartz N (2005). Dengue in travelers. Engl J Med.

[23] Hii YL (2012). Climate and dengue fever: early warning based on temperature and rainfall. Dissertation.

[24] Darren C (2013). Dengue Fever in Thailand.

[25] Char Meng Chuor Dengue fever kills 14 Cambodian children in 1st four months.

[26] World Health Organization (2014). Global Alert and Response (GAR).

[27] Kakhapakom K, Tripathi NK (2005). An information value based analysis of physical and climatic factors affecting dengue fever and dengue haemorrhagic fever incidence. Health Geogr.

[28] Frank C, Höhle M, Stark K, Lawrence J (2013). Rapid communications More reasons to dread rain on vacation? Dengue fever in 42 German and United Kingdom Madeira tourists during autumn 2012. Eurosurveillance.

[29] Hashizume M, Dewan AM, Sunahara T (2012). Hydroclimatological variability and dengue transmission in Dhaka, Bangladesh: a time-series study. BMC Infect Dis.

[30] Chang MS, Christophel EM, Gopinath D (2011). Challenges and future perspective for dengue vector control in the Western Pacific Region. Western Pac Surveill Response J.

[31] Yan WU, Member, IAENG (2008). Detect climatic factors contributing to dengue outbreak based on wavelet, support vector machines and genetic algorithm.

[32] Watts DM, Burke DS, Harrison BA (1987). Effect of temperature on the vector efficiency of Aedes Aegypti for Dengue 2 virus. Am J Trap Med Hyg.

[33] Keating J (2001). An investigation into the cyclical incidence of dengue fever. Soc Sci Med (1982).

[34] Hii YL, Rocklöv J, Wall S (2012). Optimal lead time for dengue forecast. PLoS Negl Trop Dis.

[35] Halide H, Ridd P (2008). A predictive model for dengue hemorrhagic fever epidemics. Int J Environ Health Res.

[36] Naish S, Dale P, Mackenzie JS, McBride J, Mengersen K, Tong S (2014). Spatial and Temporal Patterns of Locally-Acquired Dengue Transmission in Northern Queensland, Australia, 1993–2012. PLOS ONE.

[37] Tipayamongkholgul M, Lisakulruk S (2011). Mathuros Tipayamongkholgul1, Sunisa Lisakulruk Socio-geographical factors in vulnerability to dengue in Thai villages: a spatial regression analysis. Geospatial Health.

[38] Karim N, Munshi O, Anwar N, Alarm S (2012). Climatic factors influencing dengue cases in Dhaka city: a model for dengue prediction. Indian J Med Res.

[39] Thammapalo S, Chongsuwiwatwong V, McNeil D (2005). The climatic factors influencing the occurrence of dengue hemorrhagic fever in Thailand. Southeast Asian J Trop Med Public Health.

[40] Colón-González FJ, Fezzi C, Lake LR (2013). The effects of weather and climate change on dengue. PLoS Negl Trop Dis.

# Sub article: Perceptions of climate change and its impact on human health: an integrated quantitative and qualitative approach

**DOI:** 10.3402/gha.v7.23025

**Published:** 2014-12-08

**Authors:** Do Thi Thanh Toan, Vu Duy Kien, Kim Bao Giang, Hoang Van Minh, Pamela Wright

**Affiliations:** 1 Department of Biostatistics and Medical Informatics, Institute of Training for Preventive Medicine and Public Health, Hanoi Medical University, Hanoi, Vietnam; 2 Center for Health System Research, Hanoi Medical University, Hanoi, Vietnam; 3 Department of Health Promotion, Institute of Training for Preventive Medicine and Public Health, Hanoi Medical University, Hanoi, Vietnam; 4 Department of Health Economic, Institute of Training for Preventive Medicine and Public Health, Hanoi Medical University, Hanoi, Vietnam; 5 The Medical Committee Netherlands – Vietnam, Hanoi, Vietnam

**Keywords:** climate change, perception, health, Hanoi, Vietnam

## Abstract

**Background:**

The World Health Organization emphasized that climate change is a significant and emerging threat to public health, especially in lower income populations and tropical/subtropical countries. However, people in Asia and Africa were the least likely to perceive global warming as a threat. In Vietnam, little research has been conducted concerning the perceptions of effects of climate change on human health.

**Objective:**

The aim of this study was to explore the perceptions on climate change and its impact on human health among people in Hanoi.

**Design:**

We applied a combined quantitative and qualitative approach to study perceptions on climate change among people in Hanoi. A total of 1,444 people were recruited, including 754 people living in non-slum areas and 690 people living in slum areas of Hanoi. A structured questionnaire was used to collect quantitative data on their perceptions. In a parallel qualitative study, two focus group discussions and 12 in-depth interviews (IDs) were carried out involving 24 people from both slum and non-slum areas.

**Results:**

The majority of the respondents in the study had heard about climate change and its impact on human health (79.3 and 70.1% in non-slum and slum areas, respectively). About one third of the respondents reported that members of their family had experienced illness in the recent summer and winter compared to the same seasons 5 years ago. The most common symptoms reported during hot weather were headaches, fatigue, and dizziness; hypertension and other cardiovascular diseases were also reported. During cold weather, people reported experiencing cough, fever, and influenza, as well as pneumonia and emerging infectious diseases such as dengue and Japanese encephalitis.

**Conclusions:**

The observed high level of awareness on the links between climate change and human health may help to increase the success of the National Prevention Program on Climate Change. Moreover, understanding the concerns of the people may help policy makers to develop and implement effective and sustainable adaptation measures for Hanoi City as well as for Vietnam as a whole.

Climate change has already been observed globally, including increases in air and water temperatures, reduced number of frost days, increased frequency and intensity of heavy downpours, a rise in sea level, and reductions in snow cover, glaciers, permafrost, and sea ice (1). In 1990, the Intergovernmental Panel on Climate Change concluded that there was broad international consensus that climate change is human induced (2). However, in a study conducted by a Gallup Poll in 128 countries, still one third of residents from Africa, parts of Asia and the Middle East, and a few countries from the former Soviet Union did not agree that climate change is a result of human activities (3).

The link between population health and climate change has been demonstrated by scientists who stated that climate change poses a wide range of risks to population health. As noted by the World Health Organization, climate change is not just a threat to biological systems and the environment but a ‘significant and emerging threat to public health’, especially in lower income populations and tropical/subtropical countries (2, 4). Protecting health from the impacts of climate change is recognized as one of the defining challenges in this century (1). Perceptions on the effects of climate change on health have been studied among community members in developed (5–10) and developing countries (11–14). Haque et al. (13) found in Bangladesh that most local perceptions on climate change were consistent with the scientific evidence regarding the vulnerability of that country to climate change. The people perceived that changes in heat, cold, and rainfall had occurred over the last 5–10 years and linked these problems to identify future threats to themselves and their families and livelihoods (13).

Vietnam is one of the developing countries most affected by climate change (15, 16). The country suffers from many kinds of natural disasters that affect an estimated 1 million Vietnamese annually. The impact of climate change on individuals varies according to many factors like age, occupation, gender, education, and economic status (4, 7, 17, 18). In addition, factors that influence concern about climate change include past experiences of the impact of flooding on livelihood and observation of changes in the climate (19). Understanding the complex links between climate change and human health as well as understanding peoples’ concerns will assist policy makers to develop communication strategies to engage communities in each location most effectively to deal with the consequences of climate change. Such communication is increasingly a priority for Hanoi City, because in recent years, climate change has affected the city with extreme consequences (17).

Nonetheless, to the best of our knowledge, very little research has been conducted in Vietnam about peoples’ perceptions of the effect of climate change on human health. Therefore, the aim of this study was to explore Hanoian's perceptions of climate change and its effect on their health. We also looked for relationships between peoples’ perceptions and a set of socioeconomic characteristics that might influence their knowledge and experiences. The results of this study could be used to inform decision makers in Hanoi responsible for developing or adapting programs of action to cope with climate change.

## Methods

### Study setting

Hanoi, the capital of Vietnam, comprises 29 districts, 10 of which are urban and 19 rural. As of 2009, Hanoi's population was estimated at 6.5 million, of which 2.6 million (41%) lived in urban districts (20). The study was carried out in four urban districts in Hanoi, namely Ba Dinh, Hoan Kiem, Hai Ba Trung, and Dong Da districts, because they included a number of slum areas. We defined the slum areas according to the United Nations definition, as areas where people live in temporary houses, insecure locations, narrow spaces, or polluted environmental conditions.

### Study design and participants

This was a population-based cross-sectional study conducted from December 2012 to March 2013, applying a mixed-methods research design, quantitative and qualitative (21, 22). To obtain a wider range of household perceptions from a large number of households, a quantitative survey was conducted in 30 slum areas (randomly selected from the list of 84 slum areas) and 30 matched, nearby non-slum areas in the four urban districts. In each slum or non-slum area, about 20 households were selected; we applied a door-to-door approach after randomly selecting the first house. In each household, we selected one individual among the household members between 15 and 60 years of age by chance, and a second individual above 60 years old, if available. The non-response rate was only 2–3%, and quite similar in both slum and non-slum areas. Finally, 1,444 people were invited to participate, including 754 people in non-slum areas and 690 people in slum areas. We included a qualitative study to gain deeper insights into local perceptions and experiences of people in Hanoi. Two focus group discussions (FGDs) and 12 key informant interviews were conducted. The participants were selected purposively from both slum and non-slum areas in Hanoi. They included 16 community members, four local political leaders and four district health staff.

### Data collection

In the quantitative study, face-to-face interviews using structured questionnaires were conducted by trained medical students from Hanoi Medical University. The interviewers were supervised by senior staff from the Center for Health System Research, Hanoi Medical University. The questionnaires were developed by the research team and were piloted before being used in the field. The questions included topics such as perception of climate change, impact of climate change, changes in climate as well as illness of family members in summer and winter compared to 5 years earlier.

To characterize the socioeconomic status of the study population, we used principal component analysis to calculate a wealth index based on different variables, including ownership of house, radio, computer, internet connection, telephone, refrigerator, and so on. The wealth index was estimated separately for the non-slum and slum areas, then divided into five quintiles, ranking from the poorest to the richest.

An interview guide based on the broad themes of hot and cold weather and people's perception of climate change was used for the qualitative study. Prior to the official study, a pilot interview was carried out with one female health staff member of the slum area. Interviews were conducted at the interviewees’ house; FGDs were organized in the meeting rooms of two District Health Centers. All of the interviews were recorded both in notes and recordings, with the agreement of the interviewees, and later transcribed.

### Data analysis

Data were analyzed using Stata statistical software, version 12.1. Both descriptive and analytical statistics were applied. The Chi-square test was used to compare discrete variables. Logistic regressions were used to detect any association between people's perceptions of climate change and the impacts of climate change and the social determinants. The independent variables (considered as social determinants) consisted of area of household (slum or non-slum area), education, age group, and socioeconomic status. Significance level was set at *p*<0.05.

Data for qualitative study was transcribed and described according to the above themes of perceptions of climate change and perceptions of impact of climate change on health. Content analysis was used to analyze the interview transcripts. Content analysis is a procedure that organizes transcribed material by coding interview data into blocks that represent a common theme or new themes that emerge from the interviewee quotes (22).

### Ethical considerations

People invited to participate in the study were asked for their informed consent before they were interviewed. They were also informed that they were free to withdraw from the study at any time.

## Results

### The characteristics of the study subjects

Of the 1,444 people selected by the sampling procedure, 1,412 (97.8%) were available and agreed to participate and provided information. Table 1 shows the demographic characteristics of the study population according to living area (non-slum or slum area). The mean age of the respondents was 55.6±16.5 years (range 16–92) in the non-slum areas and 51.3±16.5 (range 16–95) in the slum areas. The distribution of gender, marital status, and wealth index was quite similar between two areas. However, there were significant differences in the distributions of age groups, education, and household size between them. People in the non-slum area seemed to have higher education levels than those in the slum areas, especially more of them had college education.

**Table 1:** Demographic characteristics of study population (n=1,412 people)

	Non-slum area (%)	Slum area (%)	*χ* ^2^	*p*
Gender				
Female	482 (65.8)	436 (64.2)	0.42	0.52
Male	251 (34.2)	243 (35.8)		
Age				
15–24	24 (3.3)	38 (5.6)	32.08	0.00
25–34	76 (10.4)	94 (13.8)		
35–44	101 (13.8)	123 (18.1)		
45–54	104 (14.2)	128 (18.9)		
55–64	195 (26.6)	139 (20.5)		
65–74	144 (19.7)	89 (13.1)		
75+	89 (12.1)	68 (10.0)		
Education				
Primary school or less	50 (6.8)	137 (20.2)	98.90	0.00
Secondary school	158 (21.6)	199 (29.3)		
High school	209 (28.5)	187 (27.5)		
College/university	316 (43.1)	156 (23.0)		
Marital status				
Unmarried	55 (7.5)	62 (9.1)	4.02	0.13
Married	563 (76.8)	490 (72.2)		
Divorced/widowed	115 (15.7)	127 (18.7)		
Household size				
1–3 members	227 (31.0)	329 (48.5)	46.21	0.00
4–6 members	458 (62.5)	309 (45.5)		
≥7 members	48 (6.6)	41 (6.04)		
Socioeconomic status (Wealth index)				
1st (poorest)	145 (19.8)	141 (20.8)	3.29	0.51
2nd	158 (21.6)	124 (18.3)		
3rd	144 (19.7)	142 (20.9)		
4th	154 (21.0)	136 (20.0)		
5th (richest)	132 (18.0)	136 (20.0)		

### Perception of climate change

Most of the survey respondents had heard about climate change and its impact. The proportion of people having heard about climate change and its impact on people was significantly higher in the non-slum area. Among those who had heard about climate change in both slum and non-slum areas, two thirds were aware of the term ‘climate change’ and interpreted it as storms, floods, deep cold, and long heat waves. The impact of climate change most often mentioned by both groups was the impact on human health (92.3 and 91.3% for slum and non-slum areas). However, a small number of respondents were not aware of this term and mentioned other phenomena such as earthquakes or air pollution as manifestations of climate change (13.1 and 12.2% in non-slum and slum areas, respectively) (Table 2).

**Table 2:** Perception of climate change and impacts of climate changes

	Non-slum area (%)	Slum area (%)	*χ* ^2^	*p*
Have you ever heard about climate change? (*n*=1,412)	581 (79.3)	476 (70.1)	15.72	0.00
If yes, what climate change could be? (*n*=1,057)				
Storm	382 (65.8)	325 (68.3)	0.76	0.39
Flood	380 (65.4)	306 (64.3)	0.14	0.71
Deep cold	374 (64.4)	289 (60.7)	1.50	0.22
Drought	274 (47.2)	231 (48.5)	0.20	0.66
Long heat wave	394 (67.8)	302 (63.5)	2.22	0.14
Other (tsunami, earthquake, pollution, hail, etc.)	76 (13.1)	58 (12.2)	0.19	0.66
Have you ever heard about impacts of climate change? (*n*=1,367)	596 (81.3)	470 (69.2)	27.85	0.00
If yes, what are impacts due to climate change? (*n*=1,066)				
Impact on health	544 (91.3)	434 (92.3)	0.39	0.53
Impact on crops	230 (38.6)	166 (35.3)	1.20	0.27
Impact on livestock	202 (33.9)	147 (31.3)	0.82	0.36
Impact on environment	448 (75.2)	324 (68.9)	5.11	0.02
Unknown	14 (2.4)	8 (1.7)	0.54	0.46

This incorrect perception was explained by one man, aged 39 years: ‘I heard from the television that the areas affected by earthquake are often highly vulnerable to climate-related problems, for example more extreme storms’.

Key informant interviews and FGDs revealed that most of the people perceived that ‘climate change is occurring’ in the form of changes in rainfall and temperature; one third perceived it as a change of strong wind during the rainy season. We also asked the participants in a FGD to give their views on the main reason for the changes in climate. Two people said, ‘It is caused mostly by human activities’, while others mentioned that ‘it is caused by both the natural changes in the environment and human activities’.

As shown in Table 3, the majority of the respondents in both non-slum and slum areas stated that changes in climate had occurred during the past 5 years. There were significant differences in perception of climate change in general between those living in non-slum and slum areas. However, there were no significant differences between the non-slum and slum areas when respondents reported about changes in patterns of illness in their families. About one-third of respondents reported that illness among family members had increased during both summer and winter compared to 5 years earlier.

**Table 3:** Perceived changes in heat and cold compared to 5 year ago (*n*=1,412)

	Non-slum area (%)	Slum area (%)	*χ* ^2^	*p*
How do you perceive change in heat in the last summer compared to 5 years ago?				
Hotter	514 (70.1)	464 (68.3)	9.48	0.02
No different	148 (20.2)	116 (17.1)		
Cooler	53 (7.2)	69 (10.2)		
Unknown	18 (2.5)	30 (4.4)		
How do you perceive change in your family members’ sickness in the last summer compared to 5 years ago?				
Sick more	254 (34.6)	260 (38.3)	3.22	0.36
No different	457 (62.4)	394 (58.0)		
Sick less	15 (2.05)	19 (2.8)		
Unknown	7 (0.95)	6 (0.9)		
How do you perceive change in heat in the last winter compared to 5 years ago?				
Colder	453 (61.8)	385 (56.7)	16.67	0.00
No different	156 (21.3)	127 (18.7)		
Warmer	87 (11.9)	134 (19.7)		
Unknown	37 (5.1)	33 (4.9)		
How do you perceive change in your family members’ sickness in the last winter compared to 5 years ago?				
Sick more	256 (43.9)	252 (37.1)	1.52	0.68
No different	447 (61.0)	402 (59.2)		
Sick less	21 (2.9)	20 (2.95)		
Unknown	9 (1.2)	5 (0.74)		

Participants in the qualitative study were also asked to reflect on their perceptions of any changes in illness among their family members compared to 5 years earlier. Most of those interviewed said that their family members, especially children, became ill more easily now than in the past.

One member of health staff said: ‘The number of children coming to our health center has been increasing recently. The hot and humid environment in Hanoi is favorable for the development of bacteria, insects and other disease carriers such as flies and rats. Children are more vulnerable to these diseases’.

According to the respondents, the most common symptoms due to hot weather were headache, fatigue, and dizziness. Hypertension and other cardiovascular diseases were thought to have become more common. The symptoms most often mentioned in relation to cold weather were cough and fever, while common diseases were pneumonia, influenza, and emerging infectious diseases such as dengue fever or Japanese encephalitis.

For example, a male house owner said, ‘Nowadays, there is an increase of infectious diseases such as dengue fever. It occurs periodically throughout the year. The hot weather and rainfall have created favorable conditions for breeding of dengue mosquitoes’.

### Perception of impacts of climate change and social determinants

Table 4 presents the results of the logistic regression analysis of correlates of perception of climate changes impact and selected social determinants. The analysis revealed that for those who lived in both slum and non-slum areas, the perception of climate change impact was significantly associated with gender and education. Men were almost 1.6 times more likely to have heard of the impacts of climate change than women. Respondents in non-slum areas who had completed high school or university were 5.8 and 12.9 times more likely to have heard of climate change than those who had not completed primary school. The odds ratios were similar for the same comparison in slum areas: 4.9 and 11.1. There was no significant association between the perception of impact of climate change and socioeconomic status.

**Table 4:** Association of impacts of climate change perception and social determinants

	Non-slum area (*n*=733)		Slum area (*n*=679)	
	
Independent variables	OR	*P*	OR	*p*
Gender				
Male	1.6	0.04	1.6	0.02
Female	1	–	1	–
Education				
Primary school or below	1	–	1	–
Secondary school	3.5	0.00	1.9	0.00
High school	5.8	0.00	4.9	0.00
College/university	12.9	0.00	11.1	0.00
Socioeconomic status (wealth index)				
1st (poorest)	1	–	1	–
2nd	1.2	0.42	2.7	0.00
3rd	0.9	0.29	1.9	0.02
4th	1.0	0.31	2.7	0.00
5th (richest)	0.6	0.17	3.4	0.00

## Discussion

Most of those living in non-slum areas and more than half living in slum areas had some perception of changes in climate and of the effect on health. According to the majority of respondents, climate change is occurring and results in an increase of temperature during the summer and decrease of temperature during the winter, compared to 5 years ago. They also had the perception that storms, floods, deep cold, and long heat waves were manifestations of climate change. Similar observations have been reported in various reports about climate change from both developed and developing countries, including Japan, Laos, the Philippines, Bangladesh, and several African countries (8, 9, 11, 12, 23). In Japan, in 2008, Aoyagi-Usui compared public opinion surveys from 1997, 2002, 2006, and 2007, and reported that people's awareness of environmental issues was gradually becoming focused on global warming; people were increasingly worried that the rising average temperature of the world would have devastating effects on human life in the next century (11). This phenomenon was also seen in Bangladesh, where the heat during summers was felt to have increased and rainfall to have decreased, compared to 5 or 10 years earlier (12).

Different population groups may have different opinions about climate change (13, 23, 24). We found that those living in non-slum areas and those with higher education levels had heard about climate change significantly more than had people living in slum areas and having less education. Carew-Reid (24) found that poor people seemed to have less knowledge about the adverse impacts of climate change. For example, poor migrants had more difficulties adapting to climate change as a result of having less knowledge, less support and fewer networks, as well as less experience with floods and storms compared to local residents (23). Chelsea and Patrick (25) reported that climate change awareness most strongly depended on the respondent's level of education. Heads of households with higher levels of education were more likely to be aware of climate change than those with lower education levels (26). Another study conducted in Vietnam among poor people suggested that women had less access to information about weather than did men; 60% of the women ‘had not heard’ weather forecast information compared to 35.3% of men, and 35.6% of women had ‘heard but not understood’ the information compared to 26.2% of the men. Limited access to early warning weather information or lack of communications may exacerbate women's difficulties in dealing with climate disaster (24).

The impact of climate change on human health has been seen in several studies worldwide (27, 28). In this study, people reported that they more easily became ill now than some years ago, although most of the symptoms and diseases mentioned were common, with the exception of the increase in emerging diseases such as the new influenzas, dengue and Japanese encephalitis. This perception might be a result of the findings from another study in Northern Vietnam, which showed that a warming climate would change seasonal structure, and a warming winter would result in changes in people's biological rhythm (27). The results of a study conducted in Australia, Canada, UK, USA, and Sweden (28) suggested that a warmer climate could have adverse impacts on people's health which could lead to negative health consequences for vulnerable groups such as old people and those suffering from cardiac diseases, while a colder climate could lead to increases in coughs/colds, headaches, asthma, pneumonia, and especially in re-emerging diseases such as dengue or Japanese encephalitis (28).

A high level of awareness on the links between climate change and human health may help to increase the success of the National Prevention Program on Climate Change. Moreover, understanding the concerns of the people may help policy makers to develop and implement effective and sustainable adaptation measures. However, our results also showed that about 10% of respondents had incorrect ideas, that earthquakes or air pollution were caused by climate change. According to Dr. Nguyen Huu Ninh of the Center for Environment Research Education and Development of Vietnam (personal communication), not many people in Vietnam fully understand about climate change, even among policy makers. Many people are still not aware of the threats of climate change and misunderstand that it is primarily an environmental issue (29).

Re-framing climate change as a threat to human health can be the principle catalyst for people to change their behavior and increase their support for climate change mitigation and adaptation policies. The public will need to learn to appreciate that climate change is a public health issue too, as it affects people's health and well-being.

This study does have some limitations. Firstly, the study was conducted in four urban districts in Hanoi, so it may not represent the perceptions elsewhere in Hanoi or Vietnam. Secondly, the issue under study is complicated and difficult to measure. People's perceptions on changing health could not be checked against real data on health and possible changes related to climate in this study. Such data could be collected in future to give more precision to the accuracy of people's perceptions. There is, however, evidence for an increase in emerging infections such as dengue and Japanese encephalitis in recent years, as shown in another study by Toàn et al. (30).

## Conclusions

Our study revealed that a high proportion of surveyed respondents in the capital city did perceive that climate change is occurring and could mention possible consequences such as heavier rainfall and higher temperatures. The most influential factor on such awareness was the level of education of the respondent; those with higher education had more knowledge about climate change and its impact. Recent climate change has affected Hanoi and may have led to an increase in health problems for its inhabitants.

### Policy recommendations

Understanding local perceptions and concerns will assist policy makers to develop effective communication strategies for each location in Vietnam. In this regard, the main lessons emerging from this study are the following:

- More education and raising awareness is needed for people to appreciate that climate change is a public health issue. A high level of awareness on the links between global environmental change and human health may help to increase the success of the National Prevention Program on Climate Change.
- The Program should make use of community groups as climate change communication channels, especially for the more excluded groups such as women and people with lower levels of education who presently have less awareness of the issues.

## References

[1] World Health Organization (2009). Protecting health from climate change. Global research priorities.

[2] IPCC (2001). Climate change: impacts, adaptation, and vulnerability.

[3] Dunlap RE, McCright AM (2008a). A widening gap: republican and democratic views on climate change. Environment.

[4] Be TT, Sinh BT, Miller F (2007). Challenges to sustainable development in the Mekong delta – regional and national policy issues and research needs.

[5] McMichael AJ, Butler CD (2007). Health promotion challenges: emerging health issues: the widening challenge for population health promotion. Health Promot Int.

[6] Preet R, Nilsson M, Schumann B, Evengård B (2010). The gender perspective in climate change and global health. Glob Health Action.

[7] Dolan AH, Walker IJ (2003). Understanding vulnerability of coastal communities to climate change related risks. J Coastal Res.

[8] Gonzalez LE, da Silveira P (1997). The people's attitudes towards global environmental phenomena: a case study. Clim Res.

[9] Leiserowitz AA (2005). American risk perceptions: is climate change dangerous?. Risk Anal.

[10] Debono R, Vincenti K, Calleja N (2012). Risk communication: climate change as a human-health threat, a survey of public perceptions in Malta. Eur J Public Health.

[11] Emch M, Feldacker C, Yunus M, Streatfield PK, DinhThiem V, Canh DG (2008). Local environmental predictors of cholera in Bangladesh and Vietnam. Am J Trop Med Hyg.

[12] Chaudhary P, Bawa KS (2011). Local perceptions of climate change validated by scientific evidence in the Himalayas. Biol Lett.

[13] Haque MA, Yamamoto SS, Malik AA, Sauerborn R (2012). Household's perception of climate change and human health risk: a community perspective. Environ Health.

[14] Alam, Khurshid, Naureen F, Wahida BA (2008). Gender, climate change and human security in Bangladesh.

[15] DANIDA (2005). Climate change in Vietnam. Country report.

[16] MONRE/PEP/UNDP (2008). Climate change adaptation and the poor. http://www.gdnonline.org/resources/UNIFEM_Vietnam_Gender_CC_FinalReport.pdf.

[17] MONRE (2008). National target program to respond to climate change (Unofficial translation of Vietnamese draft version of 13/5/2008). http://www.isgmard.org.vn/VHDocs/NationalPrograms/NTP%20RespondtoClimateChange.pDF.

[18] Bank TW (2010). Public attitudes toward climate change: findings from a multicountry poll.

[19] Vietnam Ministry of Health (MOH) (2007). National strategy on preventive medicine and master plan on health care system development. http://www.wpro.who.int/health_services/VTN_2011-2015.pdf.

[20] General Statistics Office (2012). Statistical yearbook of 2009. http://www.gso.gov.vn/default.aspx?tabid=387&idmid=3&ItemID=9860.

[21] Patton MQ (2002). Qualitative research & education methods.

[22] Côte J, Salmela JH, Baria A, Russell S (1993). Organizing and interpreting unstructured qualitative data. Sport Psychol.

[23] Aoyagi-Usui M (2008). An analysis of the effective factors for promoting proenvironmental actions from the information gain and social capital point of view. Rev Environ Econ Policy Stud.

[24] Carew-Reid J (2008). Rapid assessment of the extent and impact of sea level rise in Vietnam. http://www.icem.com.au/documents/climatechange/icem_slr/ICEM_SLR_final_report.pdf.

[25] Combest-Friedman C, Christie P, Miles E (2012). Household perceptions of coastal hazards and climate change in the Central Philippines. Journal of Environmental Management.

[26] Semenza JC, Wilson DJ, Parra J, Bontempo BD, Hart M, Sailor DJ (2008). Public perception and behavior change in relationship to hot weather and air pollution. Environ Res.

[27] Kien TM, Hanh TTT, Cuong HD, Shaw R, Rajib S, Juan MP, Joy JP (2010). Chapter 20 identifying linkages between rates and distributions of malaria, water-born diseases and influenza with climate variability and climate change in Vietnam. Climate change adaptation and disaster risk reduction: an Asian perspective (Community, Environment and Disaster Risk Management, Volume 5).

[28] Haq G, Snell C, Gutman G, Brown D (2013). Global ageing and environmental change. Attitudes, risks and opportunities.

[29] Ninh NH (2012). Coping with climate change for a sustainability development. http://www.canthostnews.vn/?tabid=306&NDID=36088&keyword=%C3%90oi-pho-voi-bien-doi-khi-hau-de-phat-trien-ben-vung.

[30] Toàn DTT, Hu W, Quang Thai P, Hoat LN, Wright P, Martens P (2013). Hot spot detection and spatio-temporal dispersion of dengue fever in Hanoi, Vietnam. Glob Health Action.

# Sub article: Influenza-like illness in a Vietnamese province: epidemiology in correlation with weather factors and determinants from the surveillance system

**DOI:** 10.3402/gha.v7.23073

**Published:** 2014-12-08

**Authors:** Dao Thi Minh An, Nguyen Thi Bich Ngoc, Maria Nilsson

**Affiliations:** 1 Institute for Preventive Medicine and Public Health, Hanoi Medical University, Hanoi, Vietnam; 2 Epidemiology and Global Health, Department of Public Health and Clinical Medicine, Umeå University, Umeå, Sweden

**Keywords:** influenza, ILI, epidemiology, time series analysis, ILI surveillance system, weather, climate

## Abstract

**Background:**

Seasonal influenza affects from 5 to 15% of the world's population annually and causes an estimated 250,000–500,000 deaths worldwide. The World Health Organization (WHO) recommends ‘sentinel surveillance’ for influenza-like illness (ILI) because it is simple and calls for standardized methods at a relatively low cost that can be implemented throughout the world. In Vietnam, ILI is a key priority for public health also because of its annually recurring temporal pattern. Two major factors, on which the spread of influenza depends, are the strain of the virus and its rate of mutation, since flu strains constantly mutate as they compete with host immune systems. In the context of global climate change, the role of climatic factors has been discussed, as they may significantly contribute to the cause of large outbreaks of ILI.

**Objectives:**

1) To describe the epidemiology of ILI in Ha Nam province, Vietnam; 2) to seek scientific evidence on the association of ILI occurrence with weather factors in Ha Nam province; and 3) to analyze factors from the Ha Nam ILI surveillance system that contribute to explaining the correlation between the ILI and the weather factors.

**Design:**

A data set of 89,270 monthly reported ILI cases from 2008 to 2012 in Ha Nam was used to describe ILI epidemiological characteristics. Spearman correlation analyses between ILI cases and weather factors were conducted to identify which preceding period of months and weather patterns influenced the occurrence of ILI cases. Ten in-depth interviews with health workers in charge of recording and reporting ILI cases at different levels of the ILI surveillance system were conducted to gain a deeper understanding of factors contributing to explaining the relation between the ILI and the weather factors.

**Results:**

The results indicated that the ILI occurred annually in all districts of the Ha Nam province in the five studied years. An epidemic occurred in 2009 with the number of cases three times higher than the average threshold. There was a relation between the ILI cases in the previous 1 month with ILI cases of the following month. A seasonal cycle of ILI and correlation between weather elements were not clearly detected. A qualitative study showed that the number of ILI cases reported by the Provincial Preventive Medicine Centre (PPMC) in Ha Nam might not have reflected the accurate number of seasonal ILI occurring in this area. This was due to three gaps in the ILI surveillance system that initially were detected through key in-depth interviews in the Duy Tien and Binh Luc districts. They reported inconsistent ways of recording and reporting ILI cases among communes, lack of ILI survey forms, and irregular and delayed feedback from the PPMC.

**Conclusions:**

There were no clear patterns of association between weather factors and ILI cases detected from the five studied years. The number of ILI cases reported by the PPMC in Ha Nam may not reflect adequately the actual number of seasonal ILI occurring in this area due to three weak points in the ILI surveillance system initially detected through the case of the Duy Tien and Binh Luc districts. These three weak points of the system should be examined by a study conducted in the remaining districts in Ha Nam.

According to the US Centre of Disease Control and the European Influenza Surveillance Scheme, ‘Influenza-like illness (ILI), also known as acute respiratory infection (ARI) and flu-like syndrome/symptoms, is a medical diagnosis of possible influenza or other illness causing a set of common symptoms such as fever, shivering, chills, malaise, dry cough, loss of appetite, body aches and nausea, typically in connection with a sudden onset of illness. Common causes of ILI include the common cold and influenza, which tends to be less common but more severe than the common cold’ (1, 2). Any clinical influenza diagnosis is in technical terms an ILI diagnosis. Mostly this is not seen as a problem as most ILI cases regardless of its causes are self-limiting and not severe (3). The 20th century experienced three huge pandemics of influenza, hereinafter called ‘ILI’. The first ILI epidemic caused by A/H1N1 virus occurred in 1918 in Spain and spread all over the world. It affected 20–40% of the worldwide population and 50 million people died (4). The second wave of ILI epidemic occurred in February 1957 and March 1958, which was caused by a new ILI virus (A/H2N2) identified in the Far East (5, 6). The third wave of ILI epidemic occurred in early 1968, caused by an ILI virus detected in Hong Kong, which was similar in some ways to the 1957 pandemic ILI virus (7).

In the 21st century, A/H5N1 ILI first infected humans in 1997 during a poultry outbreak in Hong Kong. From December 12, 2003, to April 12, 2004, the total number of confirmed cases in Thailand and Vietnam combined were 25, of which 19 were fatal (8). In spring 2009, a new ILI virus called ‘A/H1N1/2009’ spread quickly across the United States and throughout the world. There were 74 countries that were affected by the pandemic. CDC estimated that 43–89 million people had H1N1 between April 2009 and April 2010, in which there were approximately 8,870–18,300 H1N1 related deaths (4). Recently, an outbreak of human infections with a new avian influenza A (H7N9) virus was first reported in China by the World Health Organization (WHO) on April 1, 2013 (9).

According to the United Nations Framework Convention on Climate Change, the environment changes with a changing climate, for example, impacting its resilience. Climate change also has different impacts on eco systems and their reproduction and on human health (10). Some scientific studies have indicated the relationship between the ILI prevalence and climatic factors, as well as between ILI cases and climate, with the scale of space and time (11). More specifically, some studies have shown that almost all ILI cases were detected in the summer when the study sites had the greatest rainfall, the highest temperature, and most humidity, revealing that climate factors could be important in the formation of big ILI epidemics (12).

Vietnam is a tropical country experiencing several subtypes of ILI, such as H3N2, H5N1, H1N1, and B. In 2009, a new subtype of ILI, the A/H1N1 virus, appeared in Vietnam, causing an epidemic. This outbreak made the ILI situation in Vietnam more severe and complicated. By May 2010, according to the Ministry of Health, Vietnam had 11,214 cases of ILI A/H1N1/2009 with 58 deaths (13). In the Ha Nam province, an outbreak of A/H3N2 ILI with 200 cases occurred in the industrial zones in 2008 and was followed by a pandemic of A/H1N1, in which many other countries were involved. It occurred in all 116 communes with over 8,000 probable cases in 2009 (14). ILI cases in Ha Nam were reported through routine monthly surveillance reporting of 28 infectious diseases (presented in Annex 1).

This study aims to: 1) describe the epidemiology of ILI in the Ha Nam province, Vietnam; 2) seek scientific evidence on the association of ILI occurrence with weather factors in the Ha Nam province; and 3) analyze factors from the Ha Nam ILI surveillance system that contribute to explaining the correlation between ILI and weather factors.

## Material and methods

### Study area

The Ha Nam province is located in the Red River Delta area, and in 2012, it had a population of about 800,000 people. It is located at the southern gateway of Hanoi and shares borders with six other provinces. The Ha Nam province consists of five districts and one city: Duy Tien, Kim Bang, Binh Luc, Ly Nhan, Thanh Liem, and the city of Phu Ly (herein generally called six study districts). Ha Nam has a tropical monsoon climate. There are two contrasting seasons, which are summer and winter and two transition periods of spring and autumn. The annual average temperature is about 23–24°C, with an average number of hours of sunshine ranging from 1,300 to 1,500 hours/year. The average rainfall is about 1,900 mm and annual average humidity is 85% (15).

### Study design

An initial cross sectional study was conducted in 2008–2012 by reviewing ILI cases reported to the surveillance system of the Preventive Medicine System. The cross-sectional study motivated follow-up questioning, aiming to understand more deeply some of the issues and problems, identified from the quantitative results. Therefore, 10 in-depth interviews were conducted in Ha Nam, consisting of two targeted village health workers, three targeted commune health staff, three targeted district health staff, and two targeted provincial preventive medicine staff. The quantitative study and in-depth interviews were carried out in sequence and were connected.

### Data collection

ILI (International Classification of Disease-10 (ICD-10) with code J10-J11) is categorized in group B of infectious diseases in Vietnam. In the study period, ILI cases were recorded and reported monthly through four levels of the ILI surveillance system, which were from (i) the village level to (ii) the commune level to (iii) the District Health Center (DHC), and finally, to (iv) the Provincial Preventive Medicine Centre (PPMC). Reporting followed a vertical and horizontal system according to the legal provisions on the 28 infectious diseases notifiable in Vietnam (16) as presented in Annex 1. Information on ILI cases were collected through reviewing monthly reporting sheets from 2008 to 2012, which were stored at the PPMC in Ha Nam. Every sheet displayed the monthly aggregated number of ILI cases of all six districts. From these sheets with monthly aggregated ILI data, aggregated ILI cases by district and year were entered into a database, using MS Excel software. Weather data included average temperature (°C), rainfall (mm), relative humidity (%), and sun hours (hours) per month during the period 2008–2012 as recorded by the Centre of Hydrometeorology and Statistics Office of Ha Nam. The monthly aggregated ILI data set was merged with the monthly weather data, for the time series analyses.

The sample in the interview study was purposively selected with the aim of having key informants, who were able to give information to get a deeper understanding of the results identified from the quantitative analysis. Therefore, we approached health workers who were in charge of coordinating and implementing ILI surveillance activities at different levels at definite sites of the ILI surveillance system. The sites and levels selected were those that the quantitative data indicated had problems. Information of these initial interviews would direct the selection of the next persons at different levels for further information. In-depth interviews were carried out until saturation was reached, and there was information enough to suggest a hypothesis for further comprehensive qualitative studies exploring weaknesses in the surveillance system.

A bachelor student, trained and experienced in conducting in-depth interviews and previously coached by senior experienced interviewers, made all of the qualitative data collection for this study as part of her bachelor thesis. The first author of this paper, who was also the supervisor, together with the student used the quantitative data to identify themes that would be raised in the in-depth interviews. An interview guide with themes and questions was prepared and used. The questions were open ended to allow for elaboration and probing by the interviewer. After each interview ended, the student and the supervisor sat together, reviewed the data gathered, discussed issues that needed to be further investigated, and focused for the next interview. In each discussion, the supervisor also helped the student to identify, whom to invite to be interviewed next. This process was implemented for nine rounds and ended when saturation was reached after the tenth interview. From village to province level, all informants gave their informed consent to participate and the interviews were carried out at their work place.

The interviews were recorded with an electronic recorder and on average, each interview lasted 1 hour. The interviewer also took notes by hand in a notebook. The main themes focused on answering problems that were identified from the quantitative study regarding the following issues: 1) practices of health staff recording and reporting ILI cases, 2) the regulation of recording and reporting, 3) other factors of the preventive system that might influence health workers’ practices in identifying, recording and reporting ILI cases from the village- and commune-level to the provincial level, and 4) the mechanism of monitoring and giving feedback from higher levels to their subordinate levels.

### Analysis

In the quantitative analysis, the incidence rate of ILI per 100,000 people was used to describe the distribution of the disease by years and by districts over the period 2008–2012. An np-trend test was performed to examine the tendency of the epidemic from 2008 to 2012 using Stata 10 software (Stata Corporation). An np-trend test is a non-parametric test for trend across ordered groups, developed by Cuzick (18), used here to test whether the rate of ILI increased or decreased significantly through the years, when applying it to longitudinal data. The Analytical Software for Epidemiological Time Series (Epipoi) was used to show the seasonality of ILI cases over years (19). ArcGIS 9.3 was used to map ILI cases by districts in which a spectrum of colors display the average rate of ILI cases/100,000 populations. An auto-correlation (AC) function was estimated to describe the correlation between the ILI cases occurring in the preceding periods of 12 months, hereinafter called ‘lag case’, and ILI cases occurrence of current month using Stata 10.

Spearman correlation analysis was conducted on ILI cases and each of the four weather factors to identify the right preceding periods of months (hereinafter called lag times), which had an influence on the occurrence of ILI cases. The lag times with a correlation between ∣0.3∣ and ∣1∣ were selected to be included in subsequent regression models.

The in-depth interviews were transcribed verbatim. A descriptive content analysis approach focusing on the manifest content in the text was carried out to understand the essence in the data from the interviews.

### Ethical considerations

This study collected monthly aggregated flu cases without having personal information of the cases. Therefore, there was no risk of disclosing any individual information. Feedback of research results to communities in Ha Nam and recommendations from the study to the relevant agencies to take appropriate action to control ILI will be carried out after the completion of this study. In addition, informed consent for the 10 in-depth interviews in the qualitative study was obtained from the key informants.

## Results

The incidence rate of ILI per 100,000 populations ranged from 1,889 to 3,081 annually in the study period. In 2009, the highest rate of ILI was reported, followed by a gradual insignificant decrease in numbers of ILI cases in the years 2010, 2011 and 2012 with *p=*0.07 (Fig. 1). ILI occurred annually and its seasonality was unclear. There were two peaks of ILI cases in 2009, of which the second peak in October was three times higher than the average threshold presented in Fig. 2. The intra-annual cycle of the ILI epidemics occurred twice a year from 2009 until mid-2010 and is presented in Figs. 3 and 4. ILI occurred yearly in all six districts of the Ha Nam province. The average rate of ILI/100,000 people in the period 2008–2012 and over years in the Binh Luc district was always the highest, while the rate of Duy Tien district was always the lowest (Map 1). There was clearly an AC between the number of ILI cases of the previous month with the number of ILI cases of the current month (AC=0.32 with *p<*0.05; presented in Table 1). There were no statistically significant correlations between ILI and temperature, ILI and rainfall, and ILI and hours of sunshine in the study period, except for 2011.

**Fig. 1:** Incidence rate of ILI per 100,000 over the period 2008–2012.

**Fig. 2:** Cycle and seasonality of seasonal ILI from 2008 to 2012. The red line is the average number of ILI cases estimated from 2008 to 2012 . The green area expresses the number of ILI cases over the years 2008–2012 in the Ha Nam province .

**Fig. 3:** The cyclic pattern of the ILI from 2008 to 2012. The red line limits the statistical significance: . The pink line is the area of statistical significance: .  Dark blue color represents the strongest cyclic pattern.  Lighter blue color represents weaker cyclic pattern. Color density represents characteristics of seasonality, of which areas in circled pink displayed the level of statically significance. This figure indicates that the period from 2009 to mid-2010 was statistically significant. However, this period was not enough to show the ILI epidemic.

**Fig. 4:** The statistical significance of the flu cycle. The pink line is the area of statistical significance: . The back line displays the seasonality of ILI cases: . Y-axis displayed period of time (years) for the seasonality of ILI reiterated. X-axis displayed the peak of epidemic after a cycle reiterated. This figure shows four peaks of the ILI corresponding to the period of time of the ILI cycle. The ILI cycle of 1 year once, 4 years once, and 8 years once are very clear but only the cyclic pattern twice a year was significantly statistic (the area lies in the pink line).

**Fig. 5:** Seasonal ILI surveillance system in Ha Nam province from level of village to province from 2008 to 2012 (17).

**Map 1:** Average incidence rate of ILI per 100,000 people/year in Ha Nam by districts, 2008–2012. Average rate per 100,000 people in 5 years.

**Table 1:** Auto-correlation between ILI cases within 12 lags

LAG	AC	PAC	Q	Prob>Q
1	**0.32**	0.32	6.38	0.01
2	**0.01**	−0.10	6.39	0.04
3	0.02	0.04	6.39	0.09
4	0.04	0.04	6.55	0.16
5	0.06	0.04	6.84	0.23
6	0.14	0.13	8.23	0.22
7	0.09	0.01	8.79	0.26
8	0.03	0.01	8.87	0.35
9	−0.01	−0.03	8.89	0.44
10	0.05	0.07	9.09	0.52
11	0.03	−0.02	9.17	0.60
12	0.02	0.01	9.22	0.68

Note: Values in bold to display the auto-correlation between the number of ILI cases in the previous month and ILI cases in the later month (lag 1, lag 2) with p<0.05. However, this correlation is weak. The remaining values of AC were not statistic significance with p>0.05.

Therefore, the qualitative study focused on understanding if there were obvious reasons, that the stakeholders could reveal, which could potentially explain the finding of no association between weather and occurrence of ILI. It was also of interest to explore why Duy Tien and Binh Luc always had the lowest and the highest number of ILI annually. For example, could there be bias from ILI cases reported that influenced these results? To answer these questions, we conducted a tracking interview, in which we initially interviewed health workers who were responsible for ILI surveillance system at PPMC to understand mechanism of the ILI surveillance system.

The information of interviewers was labeled with numbers below in each quoted sentence (Fig. 5):

^1^A person in a leading position at PPMC in Ha Nam

^2^A person with a leading role in the health staff at PPMC in Ha Nam

^3^A person in the health staff with responsibility in summarizing and reporting ILI cases monthly in the Binh Luc district

^4^A person in a leading role of the CHS in the Duy Tien district

^5^A person in the health staff with responsibility in summarizing and reporting ILI in the Duy Tien district

^6^A VHW in Duy Tien district

^7^A health worker in Binh Luc district

^8^A health station worker in the department of control communicable diseases in DHC of Duy Tien

^9^A person with a leading role at the department of disease control in Binh Luc DHC

^10^A person with a leading role in the department of communicable disease in Duy Tien DHC

### Provincial Preventive Medicine Center

The first key informant^1^ illuminated that:

^1^ILI cases were firstly detected by Village Health Workers (VHWs) or identified by health workers at the Community Health Station (CHSs). CHSs are required to record ILI cases weekly and then report monthly to DHC according to the regulation of reporting infectious diseases. On the fifth every month, CHSs report ILI cases to DHC; on the 10th DHC report aggregated ILI cases to the PPMC and then on the 15th, the PPMC will report aggregated ILI cases to the Preventive Medicine Department, Ministry of Health. Almost all CHSs and DHCs were trained regarding recording and monitoring ILI cases. However, these capacities among districts are different and often depend on the competencies of health workers.

The following informant,^2^ said that:

^2^Capacity of health staff at the DHCs in monitoring ILI cases reported from CHSs depended on leadership, qualification and quality of health staff at each DHC … We ourselves had weak points in capacity of analyzing, interpreting, and giving feedback to the DHC.

When being asked about which level that PPMC staff thought to be the weak link of the ILI surveillance system that might induce bias. The informant^1^ said that:

^1^Communities, where ILI cases from communes were detected is the most important link but still there are issues of concern regarding ILI data.

Therefore, at the next level we decided to conduct an in-depth interview for better understanding of data quality of data at a community level in the Duy Tien and Binh Luc Districts, where quantitative data suggested that there might be problems in recording and reporting ILI cases.

### Commune Health Station (CHS)

In-depth interviews were conducted with the persons who were responsible for 28 infectious diseases surveillance in CHS in Duy Tien and Binh Luc. Through in-depth interviews with these people, we understood that ILI cases were detected, identified, recorded and reported to the CHS by three sources.

The first source was:

^3^People living around the CHS, if they get ill, they primarily visit CHS for being health examined. With ILI cases, based on clinical symptoms such as cough, fever, shortness of breath or epidemiological exposure, we could identify ILI cases at the CHS.

The second source was a network of collaborators called Village Health Workers (VHWs):

^4^Residences staying in the commune such as retired health staff or elderly people having spare time were recruited to work as VHWs. These VHWs were trained to detect and record ILI cases mostly based on clinical symptoms and their individual experiences.

The third source was:

^5^Private health clinics located in the commune, if they had patients with ILI cases (usually severe ones), they have informed the CHSs, but not many of them did that work.

From these three informants at the commune level, it was understood that ILI cases reported by CHSs were underestimated due to many influencing factors as follows:

There were ILI patients of the community but they did not go to CHSs for care.

^3^Not many people came to the CHSs because people would rather go to the district hospital directly. People with mild ILI symptoms might treat themselves without examination at CHS.

VHWs in Binh Luc recorded and reported all ILI cases at any age from three sources

^3^In Binh Luc, we identify all cases of ILI based on clinical symptoms and epidemiology exposure regardless age of patients. We hold a meeting every week with VHWs and they report numbers of ILI cases that they detect during a week. We also call the private clinics to know whether there were cases of ILI identified by these facilities. The aggregated number of ILI cases reported from all three sources were added together then be reported to the district health centre (DHC) on the 5th of each month.

However, health staff in different communes in Duy Tien had recorded and reported ILI cases inconsistently. In the CHS in Trac Van commune, Duy Tien district, health staff only recorded and reported ILI cases of children:

^4^In Duy Tien, only children with ILI were recorded and reported monthly to the DHC. Up until 2012, the PPMC noticed that Duy Tien had reported low numbers of ILI cases and then required our center to record ILI both in adults and children from 2013 upward.

While health staff in Chau Son commune of Duy Tien had recorded and reported ILI cases regardless of ages.

^5^We recorded all ILI cases at all of age.

The length of time that VHWs at Duy Tien and Binh Luc were asked to report ILI cases was quite different as follows:

^3^In the Binh Luc district, the DHC usually send their staff to the CHS to hold a meeting with health staff and VHWs every Thursday.

^4^In Duy Tien that only occurred monthly on the 25th.

### Village health workers (VHWs)

In-depth interviews with VHWs at Binh Luc and Duy Tien districts also indicated that these two districts asked VHWs to report ILI cases in different periods:

^6^In Duy Tien, we often identified ILI symptoms such as cough, fever, running nose, fatigue. Currently, there was no form of recording ILI cases except the form for A/H1N1/09 and A/H5N1. Therefore, any ILI cases identified would be taken notes on in a notebook. Numbers of ILI cases were often required to report on the 25th day every month. To be honest, I am responsible for a village of nearly 1,000 people and surely, I could not contact all people living in the village regularly. When the 25th day is coming, I tried to estimate ILI cases by ask some people living around or the pharmacist whether there were ILI cases occurred. For those ILI cases that were not severe and did not share their illness status with others, we actually could not identify them. The way we recorded ILI was to count numbers of ILI cases identified and add them up to an aggregated number and then report it to the CHS.

^7^In Binh Luc, basic individual information of ILI cases such as name, age, village, symptoms, etc. were noted in a notebook but only aggregated number was reported to the CHS. CHS often holds a meeting with village health workers on Thursday every week. If a VHW was busy and could not be present at the CHS on that day, he or she could delay to report the ILI-cases identified of that week to the following week.

In addition to different practices in recording and reporting ILI among VHWs between Binh Luc and Duy Tien, through the in-depth interviews we also realized that there were factors influencing quality of ILI data collection.

^6^I do this job with 300,000 Vietnamese dong (VND~ 15 USD) allowance per month. This is not enough to live so I have to work on some other jobs and did not much time to work for the CHS.

^7^We were responsible for a large area with a crowded population so there were two VHWs assigned for ILI surveillance in this area but each one only got a half of the monthly allowance (150,000 VND).

### District Health Centre (DHC)

Reasons for the differences in recording and reporting ILI cases between Binh Luc and Duy Tien was raised for discussion in the in-depth interviews with two DHC health staff at Duy Tien and their explanation for the differences as follows:

A health worker, who worked in the department of control communicable diseases in DHC of Duy Tien said that:

^8^VHWs were occupied by many health programs of the CHSs. Sometimes they could not attend the periodical meeting and they asked another person to come instead … Honestly, I can say that VHWs are always changed, old VHWs dropped out, and new VHWs come that make the network of VHW in variation. Duy Tien only reported ILI cases in children. This might be due to some mistakes from receiving ILI form delivered together with immunization form; the new VHWs may think that ILI cases should be identified among children only.

Aggregated numbers of ILI cases were reported to the DHC instead of individual cases and it was confirmed by health staff at the DHC of Binh Luc that:

^9^ILI has no form for recording and reporting, so only aggregated numbers were recorded and reported to DHC while the A/H1N1/2009 investigation did use a form for data collection.

One person in DHC Duy Tien indicated that:

^10^Indeed, we have to accept the aggregated numbers of ILI cases reported from the village and commune, while understanding that without using a form for recording and reporting ILI, biases would occur.

## Discussion

Although this current study indicates that ILI occurred yearly in Ha Nam from 2008 to 2012, seasonality characteristics of ILI cases were not clearly identified. While studies from all over the world showed that ILI epidemics occurred with seasonality characteristics, which marked winter peaks in most countries and regions in the northern hemisphere, such as the United States, Canada and Europe. This current study also indicated that there were characteristics of autocorrelation of ILI among months of the year. That is the correlation of ILI cases of the previous month and the current month with AC1=0.32. According to Johansson, the autocorrelation is a natural element in the process of transmission of infectious diseases and is the relationship between the number of new cases and the number of previous cases (20). A similar result was seen in studies of Soebiyanto et al. (21).

However, this current study does not indicate the relationship between weather factors and ILI, except in 2011, while some studies in the region and elsewhere showed that the spread of ILI was affected partly by weather and climate change (12, 22, 23). Looking back to the original ILI data set reported by Ha Nam preventive medicine department and standard ILI surveillance of the General Preventive Department of Vietnam (Annex 2), it could be seen that, according to this standard, any primary cases with respiratory symptoms and systemic symptoms were to be recorded and reported in order to warn of ILI outbreak. Therefore, these initial cases should receive a laboratory test. If their test results were positive with ILI and, at the same time, there were increasing numbers of similar clinical cases in the community, the epidemiologist would report that there was an ILI outbreak occurring. However, due to limited resources, the system could not test all cases that have suffered from respiratory symptom and systemic symptoms; therefore, after the initial ILI cases are confirmed, if any similar clinical cases occur during that period, they should be recorded, reported and counted as ILI cases without laboratory test. Therefore, the system called ILI surveillance and data extracted from this ILI surveillance could include cases of non-ILI infections and ILI cases. Agents of respiratory infections have differences in seasonality and distribution of transmission pathways. These issues could explain partly the result of there not being an association and no clear pattern of seasonality of ILI in Ha Nam. Besides, the effects of temperature and humidity on ILI transmission may not be equal in temperate and tropical/subtropical climate zones. However, Pham Van Hau et al., in their study of ILI in Vietnam, showed that an increase of the average temperature to 1.5°C may promote the proportion of ILI to 5% (12). Therefore, in-depth interviews conducted in this study helped in interpreting results adding new information.

Results from the in-depth interviews in the current study give initial clues for interpreting this unclear correlation. Firstly, by approaching key persons, who were in charge of conducting and implementing ILI surveillance at different levels of the system, we could identify at which level the weakness of the ILI data collection process mainly originated from. The findings suggested that the community level might have more frequent underestimations of ILI cases at the CHSs, due to several reasons.

The ILI cases surveillance in Ha Nam was passive through the text reports from the level of village and commune. However, the network of VHWs did not appear well-functioning because of the lack of VHWs in terms of quantity and quality. Influencing factors were low wages for VHWs, which forced these VHWs to do extra work for their living and as a result did not dedicate their time for the ILI surveillance work as expected. The backgrounds of VHWs were quite diverse and the variation in this group made the training inefficient. These problems may result in mistakes of Duy Tien such as some VHWs not understanding protocol clearly and only reporting ILI in children. Secondly, the time span for reporting ILI cases was inconsistent between Binh Luc and Duy Tien. Binh Luc asked their VHWs to report ILI cases weekly while Duy Tien asked for monthly ILI reporting. Being asked for a monthly report resulted in VHWs not collecting information regularly on a daily or weekly basis, which may also result in under estimation of ILI cases. Errors in reporting may have occurred consequently because the cases, which happened earlier than the point of reporting time, would have completely recovered and would not be recorded accordingly. In addition, there were ILI cases that were not so weak, did not go to the pharmacists, and/or were informed about their ILI status; therefore, if VHWs did not actively seek this information by going to the commune daily or weekly, they may not get these ILI cases’ information at all.

In Binh Luc, although the CHS asked their VHWs to report ILI cases weekly, some delays still occurred when VHWs were busy at the weekly meeting day and reported ILI cases of previous weeks on the later meetings. Thirdly, almost all ILI cases coming to a private clinic were not recorded and therefore not reported sufficiently to the CHSs.

Although in-depth interviews indicated that community level is the weak point of the ILI surveillance system, it also indicated that some weak points existed at the PPMC and DHC. The case of Duy Tien, that only ILI cases among children were recorded and reported for many years, was realized by DHC in 2012, considering that Duy Tien always had the lowest numbers of ILI cases over the years. This indicates that capacity for analyzing, interpreting, supervising, and giving feedback from the PPMC to the DHCs and from the DHCs to CHSs was very poor.

These issues above were also indicated by findings from a study on ‘Assessment of communicable disease surveillance system and pilot intervention measures’ conducted by Nguyen Thi Phuong Lien in eight provinces/cities in Viet Nam, 2008–2009, with a sample of all health units at commune, district and province level. First, it was indicated that the surveillance system lacked involvement of the network of private health offices and people in communities. Secondly, it was highlighted that the percentage of health units at commune, district and province level having weekly data reported were 26.6, 83.8, and 76.9%, respectively. Data analysis was only conducted in 31.3% at district level and in 62.5% in province level. Feedback information from the district level to CHSs was low at 7.5% while from the province level to DHC it was high at 75%. The study also showed that the quality of surveillance activities was inconsistent between all levels. The rate of units having timely and completed reports was 19.1% in communes and 43.3% at the level of district (25).

Moreover, a big issue regarding forms of recording and reporting ILI cases was also identified from our in-depth interviews. According to the national surveillance system regarding ILI cases report, all PPMCs often submitted the aggregated numbers of ILI cases monthly without any individual information, with the exception when using a survey form when there was a serious epidemic-like A/H1N1/2009 flu. This has resulted in only recording ILI cases as aggregated numbers at community level. According to the WHO and the CDC's regulation of infectious surveillance systems, individual information should be collected sufficiently, especially in the context of emerging variation of ILI in recent years (17). With only aggregated numbers of ILI cases and no form for collecting individual information might have resulted in either overlapping or lacking ILI cases recorded and reported. Without information on age, sex, geography, location, etc., which is considered basic information in a surveillance system, analyses of epidemiological characteristics of ILI cases is neither informative nor effective enough. In comparison with China, a study of Peng Yang et al. illustrated that a surveillance system for ILI and virology data was established in Beijing, from 1 September 2007 to 30 April 2008. This assisted China in the early detection of ILI, defining the distribution of ILI in the community, providing timely information about circulating strains. These data, in turn, can be used to analyze geographical, temporal, and biological differences in circulating ILI strains and assist in monitoring for emerging unusual or critical situations, such as a pandemic (26).

With all of these above issues discussed, it can be understood that the number of ILI cases reported by PPMC in Ha Nam most likely was not adequate and likely under-estimated. As such, it can partially explain why this current study did not report clear correlation between weather factors and ILI cases except in 2011, as well as not finding seasonality of ILI cases in Ha Nam.

This study used the interviews carried out at all three levels (PPMC, DHC, and CHS) as a basis to decide on which level to go deeper in to. This approach helped to reduce redundant interviews and focused more at the community level, which was considered the ‘weakest’ link in the system. Moreover, in this study, we only focused on Duy Tien and Binh Luc districts, where quantitative data suggested having issues that needed to be addressed. The focus, when interviewing key persons who were in charge of coordinating and implementing the ILI surveillance system at the three levels in these two districts, was to build hypotheses as a starting point for future comprehensive studies to be able to later learn and draw conclusions about the whole system. Although the number of in-depth interviews was limited and conducted only in the two districts, trustworthiness and saturation of information was still ensured by using a triangulation approach, in which information from one level was checked by other levels and interviews only ended when there was overlapping and repeated information at different levels and no new information identified.

## Conclusions

The ILI occurred annually in all districts of Ha Nam in 5 years of research. Especially in 2009, the epidemic occurred with the number of cases tripling compared with the average threshold. There was no association between weather factors and ILI cases for the five studied years. Seasonal cycle of ILI and correlation between weather elements and ILI cases were detected without any clear patterns. The number of ILI cases reported by the PPMC in Ha Nam may not reflect the accurate number of seasonal ILI occurring in this area due to some gaps of the ILI surveillance system that were initially detected in the case of Duy Tien and Binh Luc district. Some weak points of the system of ILI surveillance in these two districts were found including: 1) Inconsistent ways of recording and reporting ILI cases between some communes in Duy Tien. In addition, survey forms of ILI cases were not available to analyze the distribution of ILI cases following people's characteristics such as age, sex, immunization status, occupation, etc. 2) The feedback from PPMC in Ha Nam was irregular and delayed.

## Recommendations

It is important to have a good quality system for ILI surveillance, in order to protect public health in the long run. The weak points described in this study need to be further studied in other districts in order to improve the system by getting a comprehensive interpretation of the quality and quantity of ILI cases reported, in relation to the epidemiology of ILI and the scientific evidence of the association between ILI occurrence and weather factors.

## Annex 1

The system of communicable disease surveillance in Viet Nam (17).

## Annex 2

Definition of an ILI case following European Centre for Disease Prevention and Control (24).

The European-CDC ILI case definition requires meeting all of three major criteria: 1) sudden onset of symptoms; 2) at least one of the following four systemic symptoms – fever or feverish, malaise, head-ache, or myalgia; 3) at least one of the following three respiratory tract symptoms – cough, sore throat, or shortness of breath (24).

Definition of an ILI case following General Department of Prevention in Vietnam (17).

✓ Clinical criteria:

At least one of the following four systemic symptoms:

– Fever or feverishness

– Malaise

– Headache

– Myalgia

AND

At least one of the following three respiratory symptoms:

– Cough

– Sore throat

– Shortness of breath

✓ Epidemiological Criteria:

− An epidemiological link by human-to-human transmission

✓ Laboratory Criteria:

At least one the following three:

− Isolation of flu virus from a clinical specimen

− Flu specific antibody response

− Detection of flu virus nucleic acid in a clinical specimen

## References

[1] CDC (2014). Influenza like illness. http://samaritanid.com/H1N1_symptoms.html.

[2] European Influenza Surveillance Scheme (2013). Case definitions. http://www.euroflu.org/html/case_definitions.html.

[3] CDC (2001). Considerations for distinguishing influenza-like illness from inhalational anthrax. MMWR Morb Mortal Wkly Rep.

[4] Flu.gov (2013). Pandemic Flu history. http://www.flu.gov/pandemic/history.

[5] Homeland Security (2011). 1957 Asian Flu pandemic. http://www.globalsecurity.org/security/ops/hsc-scen-3_pandemic-1957.htm.

[6] The U. S. Department of Health & Services (2012). Pandemic Flu history. http://www.flu.gov/pandemic/history/index.html#1918.

[7] Homeland Security (2011). 1968 Hong Kong Flu. http://www.globalsecurity.org/security/ops/hsc-scen-3_pandemic-1968.htm.

[8] WHO (2011). Avian influenza. http://www.who.int/mediacentre/factsheets/avian_influenza/en/.

[9] CDC (2013). Avian influenza A (H7N9) virus. http://healthvermont.gov/prevent/flu/avianflu/h7n9_cdc.aspx.

[10] The Department of Natural Resources and Environment (2011). Climate change and the effects of climate change.

[11] Manangan AP (2006). Influenza prevalence in the US associated with climatic factors, analyzed at multiple spatial and temporal scales.

[12] Hau PV, Thanh Thao PT, Nhu Nguyen TM, Lan PT (2010). The relationship between climate and flu in Dak Lak. J Prev Med.

[13] General Department of Prevention in Vietnam (2010). The situation of influenza A/H1N1 in Vietnam updates to 30/8/2010. http://vncdc.gov.vn/index.php?option=com_content&view=article&id=360:tinh-hinh-cum-ah1n1-ti-vit-nam-n-3082010&catid=34:tin-hot-ng&Itemid=40.

[14] The Department of Epidemiology in Hanam (2010). The report of flu epidemic in the project strengtheningnflu surveillance system in Hanam province.

[15] Ha Nam Portal (2013). Climate in Ha Nam province. http://hanam.gov.vn/vi-vn/Pages/Article.aspx?ChannelId=4&articleID=92.

[16] Ministry of Health in Vietnam (2010). Field of epidemiology.

[17] General Department of Prevention in Vietnam (2010). Field of epidemiology.

[18] Statistical Help (2013). Cuzick's test for trend. http://www.statsdirect.com/help/default.htm#nonparametric_methods/cuzick.htm.

[19] Alonso WJ, McCormick B (2013). Analytic software for epidemiological time series (Epipoi). http://www.epipoi.info/.

[20] Brännäs K, Johansson P (1994). Time series count data regression. Taylor Francis Online.

[21] Soebiyanto R, Adimi F, Kiang R (2010). Modeling and predicting seasonal influenza transmission in warm regions using climatological parameters. PLoS One.

[22] Tamerius JD, Shaman J, Alonso WJ, Bloom-Feshbach K, Uejio CK, Comrie A (2013). Environmental predictors of seasonal influenza epidemics across temperate and tropical climates. PLoS Pathog.

[23] Viboud C, Pakdaman K, Boëlle PY, Wilson ML, Myers MF, Valleron AJ (2004). Association of influenza epidemics with global climate variability. Eur J Epidemiol.

[24] European Centre for Disease Prevention and Control (2013). Influenza case definitions. http://www.ecdc.europa.eu/en/activities/surveillance/EISN/surveillance/Pages/influenza_case_definitions.aspx.

[25] Lien NTP (2010). Assessment of communicable disease surveillance system and pilot intervention measures.

[26] Yang P, Duan W, Lv M, Shi W, Peng X, Wang X (2009). Review of an influenza surveillance system, Beijing, People's Republic of China. Emerg Infect Dis.

# Sub article: Estimates of meteorological variability in association with dengue cases in a coastal city in northern Vietnam: an ecological study

**DOI:** 10.3402/gha.v7.23119

**Published:** 2014-12-08

**Authors:** Le Thi Thanh Xuan, Pham Van Hau, Do Thi Thu, Do Thi Thanh Toan

**Affiliations:** 1 Department of Environmental Health, Institute for Preventive Medicine and Public Health, Hanoi Medical University, Hanoi, Vietnam; 2 Pasteur Institute in Ho Chi Minh City, Ho Chi Minh City, Vietnam; 3 Department of Biostatistics and Medical Informatics, Institute for Preventive Medicine and Public Health, Hanoi Medical University, Hanoi, Vietnam

**Keywords:** dengue fever, coastal city, Vietnam, weather, meteorology

## Abstract

**Background:**

Dengue fever (DF) is a vector-borne disease that is sensitive to weather and climate variability. To date, however, this relationship in coastal northern Vietnam has not been well documented.

**Objectives:**

This paper aims to examine the associations between meteorological variables and dengue incidence in Haiphong, Vietnam, over the period 2008–2012.

**Methods:**

Monthly data on dengue incidence from all commune health stations and hospitals of Haiphong (with a total population of ~1.8 million) were obtained in accordance with the WHO's recommendations over a 5-year period (2008–2012). Temperature, rainfall, and humidity were recorded as monthly averages by local meteorological stations. The association between ecologic weather variables and dengue cases was assessed using a Poisson regression model. The estimation of regression parameters was based on the method of maximum likelihood using the R program package.

**Results:**

From 2008 through 2012, 507 cases of dengue were reported. The risk of dengue was increased by sevenfold during the September–December period compared with other months over the period 2008–2012. DF cases in Haiphong were correlated with rainfall and humidity. In the multivariable Poisson regression model, an increased risk of dengue was independently associated with months with a higher amount of rainfall (RR=1.06; 95% CI 1.00–1.13 per 50 mm increase) and higher humidity (RR=1.05; 95% CI 1.02–1.08 per 1% increase).

**Conclusion:**

These data suggest that rainfall and relative humidity could be used as ecological indicators of dengue risk in Haiphong. Intensified surveillance and disease control during periods with high rainfall and humidity are recommended. This study may provide baseline information for identifying potential long-term effects and adaptation needs of global climate change on dengue in the coming decades.

Dengue fever (DF), a climate-sensitive mosquito-transmitted viral infection, is a public health problem on the rise in many tropical regions (1–6). According to the World Health Organization (WHO), DF has re-emerged globally and has increased over space and time since the 1980s. The disease is currently endemic to more than 100 countries with an estimated approximately 100 million people infected every year worldwide (7). DF outbreaks were recorded as early as the late 1940s in South East Asia (8), and the disease has become a major public health burden in the region for several decades (9, 10). If global climate continues to change, it is estimated that about 50–60% of the projected global population would be at risk of dengue transmission (11). In addition to the health consequences, the disease also creates an economic burden at family and societal levels (12).

Some weather types and climate fluctuations have been shown to contribute to increasing DF incidence (7, 13–24). A few previous studies have indicated an association between specific meteorological variables and dengue incidence using time series analyses. They found that rainfall (3, 18, 22, 25, 26), high relative humidity (3, 14, 26), and high temperature (7, 10, 16, 20, 23, 27–30) correlated with DF through increased survival time and shortening of the development cycle of mosquitoes that subsequently increased the possibility of transmission of DF among other mechanisms. The strongest weather–dengue case correlation was associated with combining the three variables: rainfall, humidity, and high temperature (22, 24, 31). Identification of the association between DF and weather could, at present, serve as a basis for early warning systems, surveillance, and prediction, and in the longer run help adapt to the potential negative impacts that climate change can have on dengue disease proliferation (10, 22, 28, 30, 32). In addition, it will be also useful for effective prevention and control strategies in regions with limited resources, such as developing countries (29, 30).

Vietnam's climate is humid and tropical, with seasonal and geographical variance (33, 34). Overall, the climate is generally favorable for mosquitoes to transmit DF. DF was first described in Vietnam in 1958 (35) and the first reported outbreak occurred in southern Vietnam in 1963 (36). Dengue transmission occurs throughout the year in Vietnam, with peak numbers of cases (72% of total cases) reported between June and November. However, there are regional differences in the climate in Vietnam and therefore seasonal transmission patterns can vary. The Northern and Central Highland regions have a cool, dry winter from December to March each year, and dengue notifications are low during this time, whereas in southern Vietnam that has a warm stable climate throughout the year, the peak transmission occurs between July and September, coinciding with the rainy season (37).

Previous studies found that high temperature and rainfall were associated with the increase of dengue case in Vietnam (1, 12, 37–42). However, the association between weather and dengue and, subsequently, climate changes and DF in a coastal city in northern Vietnam is currently not well understood (42). The current paper aims to examine the relationship between weather factors and DF incidence among the population in Haiphong, a coastal city in northern Vietnam, over the period 2008–2012. The findings of this study could result in better control planning, surveillance, and prevention strategies to date in the region, and to the development of adaptation strategies to minimize potential negative impacts of climate change on dengue incidence.

## Methods

### Study settings

The study was conducted in Haiphong (Fig. 1), with the total square of 152.300 ha. The administrative levels in Vietnam are nation, province, district, and commune, and there are five biggest provinces in Vietnam also named city. Haiphong city has a population of about 1.837 million people in 2009 (43). Haiphong's climate is typically hot, humid, and rainy. The summer period from May to September receives highest amount of rainfall in the year (1,600–1,800 mm rainfall/year). The average temperature during the year was 23–26°C with the highest figure in June and July. The annual average humidity was about 80–85% with levels peaking in July, August, and September and lowest in December and January.

**Fig. 1:** Map of Vietnam showing Haiphong province (in dark grey area).

### Data collection

In Haiphong, there is a network of health stations located in each commune. Each commune health station is in charge of dengue surveillance and reports the monthly surveillance data with dengue case notifications to the district level and later to Haiphong city's Center for Preventive Health. This surveillance system was mainly carried out by government staff, not by private practitioners.

Meteorological data, including temperature (°C), amount of rainfall (mm), and relative humidity (%), were obtained from the local bureaus of meteorology across Haiphong. Meteorological data in Haiphong comes from nine meteorological stations and 23 rain gauge stations distributed around this province. Data from these stations were aggregated to district, then city. The data represent monthly averages for each year during the study period 2008–2012.

### Surveillance and data analysis

The main aim of data analysis was to describe the dengue incidence and its association with weather variables. The outcome considered in the analysis was the actual number of dengue cases of the whole city, while the weather variables were monthly local (city and district), temperature, rainfall, and humidity. Because the number of dengue cases was small relative to the provincial population, it is reasonable to assume that the distribution of dengue cases followed the Poisson distribution. Accordingly, a zero-inflated Poisson regression model was used to model the relationships between the potential risk factors and dengue cases (44). Zero-inflated Poisson regression is a model for count data (number of cases) with excess zeros (districts had no cases in months), it assumes that with probability *p*, the only possible observation is 0, and with probability 1 – *p*, a Poisson (λ) random variable is observed. In this model, the log incidence of dengue, log(Y), was assumed to be related to a risk factor *X* by an additive linear function as follows: Log(Y)=*a*+*bX*+*e*, where *a* and *b* are regression parameters to be estimated from the observed data, and *e* represents the residual not explained by the variable *X*. As temperature, rainfall and humidity could be varied by month and locality, the relationship between these variables and dengue disease counts were adjusted for population, time (month, season, year), and area (urban, rural or island districts). Population was put in the model as an offset (log). The estimation of regression parameters was based on the method of maximum likelihood with the R program package (45). The fitted data from an optimal model was evaluated to the observed dengue disease counts by correlation and mean square error. Non-linear relationships such as polynomial regression analysis and square for satisfying normal distribution and stabilizing variance were considered but finally a model was chosen based on Akaike information criterion (AIC) (Table 3). Different types of functions, including linear, non-linear relationship, or data transformation, were used to examine factors related to dengue disease counts, and the best fit model was selected based on AIC; the model with the smaller AIC has the better fit.

## Results

During the follow-up period (2008–2012), there were 507 cases of dengue reported in Haiphong, making the morbidity rate of 27.2 per 100,000 persons. The number of cases strongly fluctuated from year to year (Fig. 2) without any apparent systematic trend, and between months within a year. There was an epidemic in 2009 that accounted for 51.7% of cases during the surveillance period (Fig. 2).

**Fig. 2:** Total number of reported dengue fever cases in Haiphong, 2008–2012.

Within a year, the number of dengue cases by month during the 2008–2012 period is shown in Table 1 and Fig. 3. The number of cases reported with a peak in the September–December period; from 2008 to 2012, the total number of dengue cases recorded during the September–December period accounted for 78% of total cases. On the other hand, the risk of dengue was increased by sevenfold during the September–December period compared with the other month.

**Fig. 3:** Number of reported dengue fever cases in Haiphong by month, 2008–2012.

**Table 1:** Meteorological factors and occurrence of dengue cases stratified by month

Month	No. of cases^a^	Temperature^b^ (°C)±SD	Rainfall^b^ (mm)±SD	Humidity^b^ (%)±SD
Jan	8	15.0±1.5	38.5±31.9	86.2±5.1
Feb	14	17.1±2.8	19.3±9.4	91.0±2.8
Mar	6	19.3±1.8	45.2±29.8	91.0±1.3
Apr	36	23.1±0.8	95.1±56.4	91.4±2.1
May	11	26.4±1.03	217.5±117.1	89.2±1.2
Jun	3	28.6±0.8	202.1±85.0	87.0±3.4
Jul	10	28.7±0.5	209.4±51.3	87.4±1.5
Aug	25	27.9±0.6	358.4±149.0	90.2±2.1
Sep	92	27.1±0.5	296.9±80.5	88.8±2.5
Oct	135	25.3±1.1	83.1±54.2	84.6±3.5
Nov	115	22.0±1.2	37.2±22.1	80.4±5.4
Dec	52	18.5±1.0	21.6±10.8	82.8±2.8
Total	507	23.2±4.8	137±133	87.5±4.5

a Data are total number of cases tallied from 2008 to 2012.

b Data are averages of 5 years (2008–2012).

Results of univariate analysis (Table 2) showed that after adjusting for seasonality, the risk of dengue was significantly associated with increased rainfall and increased humidity. The risk ratio was from 1.02 to 1.03 (*p*<0.05).

**Table 2:** Risk factors RR (95%CI) for dengue incidence in Haiphong: univariate analysis

Risk factors	Unit of comparison	RR (95%CI)	*p*
Temperature	Per 1°C increase	1.02 (0.99–1.05)	>0.05
Rainfall	Per 50 mm increase	1.03 (1.00–1.07)	<0.05
Humidity	Per 1% increase	1.02 (1.00–1.05)	<0.05

There was a significant correlation between monthly mean temperature and rainfall with *r*=0.68; *p*<0.00001 (Fig. 4); a multivariable Poisson regression model was fitted to the data to search for independent factors.

**Fig. 4:** Scatter plot of monthly mean temperature and rainfall.

In the multivariable Poisson regression model (Table 3), an increased risk of dengue was independently associated with months with a higher amount of rainfall (RR=1.06; 95% CI 1.00–1.13 per 50 mm increase) and higher humidity (RR=1.05; 95% CI 1.02–1.08 per 1% increase). The final Poisson regression model (Table 3) with two meteorological variables showed 11.7% climate factors contributing to the occurrence of dengue cases.

**Table 3:** Risk factors RR (95%CI) for dengue incidence in Haiphong: multivariate analysis

Risk factors	Unit of comparison	RR (95%CI)	*p*
Mean temperature	Per 1°C increase	0.97 (0.94–1.01)	>0.10
Rainfall	Per 50 mm increase	1.06 (1.00–1.13)	<0.05
Humidity	Per 1% increase	1.05 (1.02–1.08)	<0.001

## Discussion

A previous study found that climate factors had positive effects in increasing DF transmission in Vietnam (39–42). Particularly, rainfall and relative humidity affected the rates of biological development, feeding, reproduction, population density, and survival of Aedes mosquitoes, thus increasing the DF transmission. The present study illustrated DF reported in a coastal city in northern Vietnam with the highest number of cases from September to December over the period 2008–2012. Rainfall and relative humidity were correlated with an increased occurrence of DF, while elevated temperature was not associated in this study.

Seasonality in the incidence of DF has been seen in a number of countries that can be explained by seasonal variation of climate factors. A study in Singapore reported that dengue incidence was generally higher during the June–October period (30). A study in Trinidad in the period 2002–2004 illustrated a dengue season between June and November (25). In Vietnam, previous studies also demonstrated that most cases were reported during the rainy season, July–December (1, 39, 41). However, the number of DF cases in the present study does not increase at the beginning of the high-rainfall and relative humidity in Haiphong. It was observed that the rainfall (358.4 mm) and relative humidity (90.2%) reached the highest level in August then started decreasing in September, when the number of DF cases started increasing. Previous studies have reported that dengue epidemics take several months to reach a level where they can be recognized as the result of previous climate conditions (46, 47). This finding provides data for further investigation of ‘delay effects’ of rainfall and relative humidity for better prediction, planning, and intervention.

Our result is consistent with previous studies, which found that rainfall and humidity were associated with the number of DF incidence in developing countries (3, 14, 18, 22, 25, 26). Rainfall and relative humidity are found to increase survival time and shorten the development cycle of mosquitoes (3), resulting in an increased number of breeding sites and adult female mosquitoes (7, 13–24). However, heavy rainfall may also reduce the survival rate of adult mosquitoes. An increase in the number of mosquitoes increases the probability of DF transmission (16). Our results implied that DF-control programs and surveillance need to be intensified during the period with high rainfall and high relative humidity, even when there is no apparent increase in the cases of DF.

In this study, the correlation between temperature and DF incidence was not significant. This result is similar to a study in Trinidad 2007 (25) and Metro Manila 1996–2005 (18) and in the Philippines (48). However, previous studies found that temperature was associated with the occurrence of DF (7, 10, 16, 20, 23, 27–30). Particularly, the variable temperature (maximum and minimum) was the best predictor for the increased number of dengue cases in Singapore (23). However, in this study, the maximum and minimum temperatures for data collection were not available with recommendations for future analysis. Previous studies in Vietnam also found the temperature–dengue relationship in Hanoi (41) and Central Highlands (39). The difference in our study might be explained by the fact that the range of mean temperature in Haiphong was lower than other areas. In fact, being a coastal area, the temperature in Haiphong is often 1°C higher in winter and 1°C lower in summer compared to Hanoi capital (43). Furthermore, a study has shown that dengue viruses may reduce incubation time in mosquitoes from approximately 2 to 1 week at temperatures of 32°C and above (49), while the maximum of monthly mean temperature in our study was 29°C. It suggested that daily temperature data is needed if we want to measure the temperature–dengue relationship in a coastal area.

This study should be interpreted within the context of some strengths and potential limitations. The strength of this study is that it could ascertain most (if not all) dengue cases in Haiphong based on available public surveillance system. The health workers at all levels in the city are very familiar with DF identification and reporting systems. As a result, the low case-fatality rate and the standard clinical case definition for dengue cases in the whole city have been used for more than 10 years without any substantial change (6). However, the study used monthly data for DF cases, which might not be the ideal compared to daily counts (29). In addition, data based on surveillance systems might be underestimated. To our knowledge, there were a number of dengue patients treated in private health services that could not be recorded. A study of the diagnosis of acute undifferentiated fever in Vietnam showed that acute dengue was found in ~34% cases, which suggests the possibility of underreporting of dengue in commune health stations (50). WHO classification schemes in 1997 and 2009 had high sensitivity but lacked specificity (51). This study has demonstrated the effects of climate factors on the occurrence of DF but has not considered socio-economic, immunological determinants that have contributed to dengue (4, 52) and some other factors such as degree of urbanization (53), El Niño (19, 22), and mosquitoes index (31). The study to measure the association between DF and other social–economic variables needs to be investigated further.

## Conclusion

In summary, this study measured the correlation of meteorological factors–DF in Haiphong. Rainfall and relative humidity could be used as ecological indicators of dengue risk in Haiphong. Intensified control programs and surveillance during rainfall and high relative humidity seasons are necessary preventive measures. This study may provide baseline information for identifying the potential long-term effects of global climate change on dengue expected in the coming decades.

## References

[1] Cuong HQ, Vu NT, Cazelles B, Boni MF, Thai KT, Rabaa MA (2013). Spatiotemporal dynamics of dengue epidemics, southern Vietnam. Emerg Infect Dis.

[2] Guzman A, Isturiz RE (2010). Update on the global spread of dengue. Int J Antimicrob Agents.

[3] Nitatpattana N, Singhasivanon P, Kiyoshi H, Andrianasolo H, Yoksan S, Gonzalez JP (2007). Potential association of dengue hemorrhagic fever incidence and remote senses land surface temperature, Thailand, 1998. Southeast Asian J Trop Med Public Health.

[4] Thai KT, Anders KL (2011). The role of climate variability and change in the transmission dynamics and geographic distribution of dengue. Exp Biol Med (Maywood).

[5] WHO (2012). Dengue and dengue haemorrhagic fever. http://www.who.int/mediacentre/factsheets/fs117/en/.

[6] WHO/TDR (2009). Dengue: guidelines for diagnosis, treatment, prevention and control.

[7] Chowell G, Sanchez F (2006). Climate-based descriptive models of dengue fever: the 2002 epidemic in Colima, Mexico. J Environ Health.

[8] Gubler DJ (1998). Dengue and dengue hemorrhagic fever. Clin Microbiol Rev.

[9] Ooi EE, Gubler DJ (2009). Dengue in Southeast Asia: epidemiological characteristics and strategic challenges in disease prevention. Cad Saude Publica.

[10] Hii YL, Rocklov J, Ng N, Tang CS, Pang FY, Sauerborn R (2009). Climate variability and increase in intensity and magnitude of dengue incidence in Singapore. Glob Health Action.

[11] Hales S, de Wet N, Maindonald J, Woodward A (2002). Potential effect of population and climate changes on global distribution of dengue fever: an empirical model. Lancet.

[12] Harving ML, Ronsholt FF (2007). The economic impact of dengue hemorrhagic fever on family level in Southern Vietnam. Dan Med Bull.

[13] Fuller DO, Troyo A, Beier JC (2009). El Nino Southern Oscillation and vegetation dynamics as predictors of dengue fever cases in Costa Rica. Environ Res Lett.

[14] Halide H, Ridd P (2008). A predictive model for dengue hemorrhagic fever epidemics. Int J Environ Health Res.

[15] Jetten TH, Focks DA (1997). Potential changes in the distribution of dengue transmission under climate warming. Am J Trop Med Hyg.

[16] Johansson MA, Dominici F, Glass GE (2009). Local and global effects of climate on dengue transmission in Puerto Rico. PLoS Negl Top Dis.

[17] Promprou S, Jaroensutasinee M, Jaroensutasinee K (2005). Climatic factors affecting dengue haemorrhagic fever incidence in Southern Thailand. Dengue Bull.

[18] Su GLS (2008). Correlation of climatic factors and dengue incidence in Metro Manila, Philippines. AMBIO.

[19] Cazelles B, Chavez M, McMichael AJ, Hales S (2005). Nonstationary influence of El Niño on the synchronous dengue epidemics in Thailand. PLoS Med.

[20] Descloux E, Mangeas M, Menkes CE, Lengaigne M, Leroy A, Tehei T (2012). Climate-based models for understanding and forecasting dengue epidemics. PLoS Negl Trop Dis.

[21] Githeko AK, Lindsay SW, Confalonieri UE, Patz JA (2000). Climate change and vector-borne diseases: a regional analysis. Bull World Health Organ..

[22] Zambrano LI, Sevilla C, Reyes-Garcia SZ, Sierra M, Kafati R, Rodriguez-Morales AJ (2012). Potential impacts of climate variability on dengue hemorrhagic fever in Honduras, 2010. Trop Biomed.

[23] Pinto E, Coelho M, Oliver L, Massad E (2011). The influence of climate variables on dengue in Singapore. Int J Environ Health Res.

[24] Cassab A, Morales V, Mattar S (2011). [Climatic factors and cases of dengue in Monteria, Colombia: 2003–2008]. Rev Salud Publica (Bogota).

[25] Chadee DD, Shivnauth B, Rawlins SC, Chen AA (2007). Climate, mosquito indices and the epidemiology of dengue fever in Trinidad (2002–2004). Ann Trop Med Parasitol.

[26] Wiwanitkit V (2006). An observation on correlation between rainfall and the prevalence of clinical cases of dengue in Thailand. J Vector Borne Dis.

[27] Chen SC, Hsieh MH (2012). Modeling the transmission dynamics of dengue fever: implications of temperature effects. Sci Total Environ.

[28] Colon-Gonzalez FJ, Lake IR, Bentham G (2011). Climate variability and dengue fever in warm and humid Mexico. Am J Trop Med Hyg.

[29] Hii YL, Rocklov J, Wall S, Ng LC, Tang CS, Ng N (2012). Optimal lead time for dengue forecast. PLoS Negl Trop Dis.

[30] Hii YL, Zhu H, Ng N, Ng LC, Rocklov J (2012). Forecast of dengue incidence using temperature and rainfall. PLoS Negl Trop Dis.

[31] Yi B, Zhang Z, Xu D, Xi Y (2003). [Relationship of dengue fever epidemic to Aedes density changed by climate factors in Guangdong Province]. Wei sheng yan jiu [J Hyg Res].

[32] Dibo MR, Chierotti AP, Ferrari MS, Mendonca AL, Chiaravalloti Neto F (2008). Study of the relationship between *Aedes* (Stegomyia) *aegypti* egg and adult densities, dengue fever and climate in Mirassol, state of Sao Paulo, Brazil. Memorias do Instituto Oswaldo Cruz.

[33] United Nations (2011). Climate change Fact Sheet: the effects of climate change in Viet Nam and the UN's Responses. http://www.un.org.vn/en/publications/cat_view/130-un-viet-nam-joint-publications/209-climate-change-joint-un-publications.html.

[34] Ministry of Natural Resources and Environment (2009). Climate change, sear level rise scenarios for Vietnam.

[35] Mihov C, Tuong CV, Tuong HP (1959). A propos d une epidemie du type des fievres hemorragiques a Hanoi. Focia Medica.

[36] Ha DQ, Huan TQ (1997). Dengue activity in Vietnam and its control program, 1997–1998. Dengue Bull.

[37] Ha DQ, Tien NT, Huong VT, Loan HT, Thang CM (2000). Dengue epidemic in southern Vietnam, 1998. Emerg Infect Dis.

[38] Cuong HQ, Hien NT, Duong TN, Phong TV, Cam NN, Farrar J (2011). Quantifying the emergence of dengue in Hanoi, Vietnam: 1998–2009. PLoS Negl Trop Dis.

[39] Pham HV, Doan HT, Phan TT, Minh NN (2011). Ecological factors associated with dengue fever in a Central Highlands province, Vietnam. BMC Infect Dis.

[40] Thai KT, Cazelles B, Nguyen NV, Vo LT, Boni MF, Farrar J (2010). Dengue dynamics in Binh Thuan province, southern Vietnam: periodicity, synchronicity and climate variability. PLoS Negl Trop Dis.

[41] Toan do TT, Hu W, Quang Thai P, Hoat LN, Wright P, Martens P (2013). Hot spot detection and spatio-temporal dispersion of dengue fever in Hanoi, Vietnam. Glob Health Action.

[42] Tsuzuki A, Vu TD, Higa Y, Nguyen TY, Takagi M (2009). High potential risk of dengue transmission during the hot-dry season in Nha Trang City, Vietnam. Acta Trop.

[43] GSO (2009). Census of population and housing in Vietnam in 2009.

[44] Yang Y, Kang J, Mao K, Zhang J (2007). Regression models for mixed Poisson and continuous longitudinal data. Stat Med.

[45] R D Core Team (2005). R: A language and environment for statistical computing.

[46] Guzman MG, Kouri G, Valdes L, Bravo J, Alvarez M, Vazques S (2000). Epidemiologic studies on Dengue in Santiago de Cuba, 1997. Am J Epidemiol.

[47] Newton EA, Reiter P (1992). A model of the transmission of dengue fever with an evaluation of the impact of ultra–low volume (ULV) insecticide applications on dengue epidemics. Am J Trop Med Hyg.

[48] Su GL (2008). Correlation of climatic factors and dengue incidence in Metro Manila, Philippines. Ambio.

[49] Watts DM, Burke DS, Harrison BA, Whitmire RE, Nisalak A (1987). Effect of temperature on the vector efficiency of *Aedes aegypti* for dengue 2 virus. Am J Trop Med Hyg.

[50] Phuong HL, de Vries PJ, Nga TT, Giao PT, Hung le Q, Binh TQ (2006). Dengue as a cause of acute undifferentiated fever in Vietnam. BMC Infect Dis.

[51] Chaterji S, Allen JC, Chow A, Leo YS, Ooi EE (2011). Evaluation of the NS1 rapid test and the WHO dengue classification schemes for use as bedside diagnosis of acute dengue fever in adults. Am J Trop Med Hyg.

[52] Astrom C, Rocklov J, Hales S, Beguin A, Louis V, Sauerborn R (2012). Potential distribution of dengue fever under scenarios of climate change and economic development. EcoHealth.

[53] Wu PC, Lay JG, Guo HR, Lin CY, Lung SC, Su HJ (2009). Higher temperature and urbanization affect the spatial patterns of dengue fever transmission in subtropical Taiwan. Sci Total Environ.

# Sub article: The effect of temperature on cardiovascular disease hospital admissions among elderly people in Thai Nguyen Province, Vietnam

**DOI:** 10.3402/gha.v7.23649

**Published:** 2014-12-08

**Authors:** Pham Ngan Giang, Do Van Dung, Kim Bao Giang, Hac Van Vinhc, Joacim Rocklöv

**Affiliations:** 1 Administration of Science technology and Training, Ministry of Health, Hanoi, Vietnam; 2 Department of Medical Statistics, University of Medicine and Pharmacy at Ho Chi Minh city, Ho Chi Minh City, Vietnam; 3 Institute for Preventive Medicine and Public Health, Hanoi Medical University, Hanoi, Vietnam; 4 Faculty of Public Health, University of Medicine and Pharmacy, Thai Nguyen, Vietnam; 5 Epidemiology and Global Health, Department of Public Health and Clinical Medicine, Umeå University, Umeå, Sweden

**Keywords:** hospital admission, cardiovascular diseases, temperature, distributed lag non-linear model

## Abstract

**Background:**

Projected increases in weather variability due to climate change will have severe consequences on human health, increasing mortality, and disease rates. Among these, cardiovascular diseases (CVD), highly prevalent among the elderly, have been shown to be sensitive to extreme temperatures and heat waves.

**Objectives:**

This study aimed to find out the relationship between daily temperature (and other weather parameters) and daily CVD hospital admissions among the elderly population in Thai Nguyen province, a northern province of Vietnam.

**Methods:**

Retrospective data of CVD cases were obtained from a data base of four hospitals in Thai Nguyen province for a period of 5 years from 2008 to 2012. CVD hospital admissions were aggregated by day and merged with daily weather data from this period. Distributed lag non-linear model (DLNM) was used to derive specific estimates of the effect of weather parameters on CVD hospital admissions of up to 30 days, adjusted for time trends using b-splines, day of the week, and public holidays.

**Results:**

This study shows that the average point of minimum CVD admissions was at 26°C. Above and below this threshold, the cumulative CVD admission risk over 30 lag days tended to increase with both lower and higher temperatures. The cold effect was found to occur 4–15 days following exposure, peaking at a week's delay. The cumulative effect of cold exposure on CVD admissions was statistically significant with a relative risk of 1.12 (95% confidence interval: 1.01–1.25) for 1°C decrease below the threshold. The cumulative effect of hot temperature on CVD admissions was found to be non-significant and was estimated to be at a relative risk of 1.17 (95% confidence interval: 0.90–1.52) for 1°C increase in the temperature. No significant association was found between CVD admissions and the other weather variables.

**Conclusion:**

Exposure to cold temperature is associated with increasing CVD admission risk among the elderly population.

The changing of the climate system is well documented and complex. It includes changes in the variability and average temperature, humidity, precipitation, and sea level. Global climate change is part of human-induced global environmental changes caused by greenhouse emissions aggravated by deforestation and ocean saturation (1).

The impact of climate change is likely to be crucial in water resources, agriculture, forestry, fishery, energy, transportation, and health sectors (1–4). Indirect health impacts are also caused by changing microbial ecology (1, 5), which increases risk of infectious diseases such as malaria and dengue fever (6, 7), and diarrheal diseases (8). Direct human health impacts include increased mortality and morbidity as results of heat waves, extreme weather events, and temperature-enhanced levels of urban air pollutants. The impacts of heat and cold temperatures have been documented in international studies (9, 10) and studies carried on in North America (11–13), Europe (14, 15), Asia (16, 17), and Australia (18–20), with a significant effect of heat waves on mortality and morbidity being described. In developed countries, there have been several studies on the specific effects of extreme temperatures on stroke and cardiovascular events (11, 21–23). There have been pathophysiologic explanations of these effects. Low temperature causes blood vessels to narrow, which increases blood pressure and the risk of stroke and other cardiovascular events (24) while high temperature causes blood vessels to dilate, which increases cardiac output and risk of decompensate heart failure (25, 26). Both low and high temperatures put stress on the cardiovascular system, especially among the elderly with limited adaptive responses, and increase the risk of coronary heart disease (27, 28).

Impacts of climate change will likely affect poor nations (3, 4) and poor people the most (25). However, in developing countries, studies on direct impacts of climate change on human health, which help to develop policy to mitigate the problems, are scanty. We have not found any quantitative publication on the impact of extreme temperatures on cardiovascular morbidity in Vietnam.

Vietnam has historically been a poor country, but has lately experienced a rapid economic, demographic, and epidemiological transition (29). Vietnam has been traditionally a country with mainly communicable diseases, but recently a mixed pattern of communicable (including classical and emerging infectious diseases) and non-communicable diseases (e.g. cardiovascular disease, cancer, diabetes, mental illness) are characterizing the current epidemiological health profile of the population (30). Geographically, Vietnam is situated in a tropical and monsoon area of the Southeast Asia peninsula. It lies in the tropic inner belt of the Northern hemisphere and is profoundly impacted by the Sea of East Vietnam. Sun elevation angle at noon time is lesser and length of day light is more varied, thus the radiation balance is lower and more varied in the North of Vietnam than in the South of Vietnam. Therefore, the temperature in winter is lower and the seasonal variation in temperature is higher in the North than in the South (31). For example, in Ho Chi Minh city, the average high temperature in May is 33°C and the average low temperature in December is 22°C, whereas in Thai Nguyen, the average high temperature in June is 32°C and the average low temperature in January is 11°C (32). Annual average precipitation commonly is within the range of 1,400–2,400 mm (31).

In this study, we aim to find out the relationship between weather variables such as hours of sunshine, relative humidity, temperature, evaporation, rainfall, and CVD hospital admissions among the elderly in Thai Nguyen province by analyzing the medical records of daily hospitalizations over the period 2008 to 2012.

## Methods

This study was conducted in Thai Nguyen, a northern province of Vietnam with a total population of 1,131,300. Thai Nguyen includes a city as the center of the province and eight rural districts. The population density is 318 km^2^. This density is higher than that of the whole country which is 259. The number of males per 100 females was 97.6. Nine percent of the population was aged 60 or above (33).

Daily weather data were obtained for the Thai Nguyen province over the period 2008 to 2012 from Vietnam National Climate and Weather Center, Hanoi. Selected weather data include daily high temperature, daily low temperature, daily average temperature (degrees celsius), daily average air pressure (mbar), daily average humidity (%), daily evaporation (mm), daily precipitation (mm), and daily sunshine time (hours). The daily weather parameters were averaged over all weather stations in the province to produce a geographically representative exposure measure.

All hospital admissions due to cardiovascular diseases (CVD) over the 5-year period were included in the study. The hospital admissions were collected from four national and provincial hospitals of Thai Nguyen. There is one national hospital in this province named Thai Nguyen National General Hospital (TNGH), and three provincial hospitals referred to as hospital A, hospital C, and hospital of Corporation of Iron and Steel. Hospital admission data were extracted from computerized patient database by Departments of Planning and Informative Center. Data include patients’ age, sex, date of admission, and final diagnosis (as made by attending physicians at discharge). CVD cases included acute myocardial infarction, angina pectoris, congestive heart failure, hypertension, and stroke. All CVD hospital admissions were recorded, and from this all 18,975 elderly (>60 years) patient cases were used in data analysis. In our study, we defined elderly cases as people aged 60 or above. This definition is consistent with that stipulated in The Law of Elderly of Vietnam (34).

Statistical analysis was done using R software version 3.0.1. Descriptive statistics for the weather variables and number of CVD admissions are presented in Tables 1 and 2. Spearman's correlation coefficients was used for exploring the monotonic relation between daily admissions and weather variables. Pearson correlation coefficients between the weather variables was used for interpreting if daily admissions are associated with two or more weather variables. Distributed lag non-linear models (DLNMs) were used to estimate the association between CVD hospital admissions and lags of weather variables, adjusted for time trends using natural cubic splines, day of the week, and public holidays with the R package dlnm (35, 36). To account for possible delayed associations, we examined the impact of weather up to 30 days before the admissions. To derive specific estimates of heat and cold slopes, the DLNM was applied with either a ‘V’- or ‘U’-shaped piecewise linear exposure–response relationship. This relationship involved specifying a single or double threshold for temperature and then estimating a log-linear change in risk of admissions above (or below) the hot (or cold) threshold(s).

**Table 1:** Description of daily weather and CVD hospitalized cases during 2008–2012 in Thai Nguyen province

Variables	Mean	SD	5%	50%	95%
Minimum temperature (°C)	21.0	5.3	10.8	22.6	27.4
Average temperature (°C)	23.6	5.5	13.0	25.1	30.4
Maximum temperature (°C)	27.5	6.2	15.6	28.8	35.2
Average humidity (%)	93.4	5.9	81	95	98
Evaporation (mm)	3.13	1.50	0.8	3.1	5.7
Precipitation (mm)	4.7	13.7	0.0	0	28.9
Sunshine time	3.53	3.54	0.0	2.5	9.8
Air pressure (mb)	1006.8	6.8	996.7	1006.4	1018.9
No. of daily CVD admissions	19.4	9.4	6		35
No. of daily elderly CVD admissions	10.4	5.9	2		21

**Table 2:** Percentage and number () of CVD hospital admissions and number of elderly CVD hospital admissions during the years, 2008–2012

Year	Elderly (*N*=18,975)	Total (*N*=35,353)
2008	16.4% (3,111)	16.9% (5,981)
2009	18.1% (3,437)	19.8% (7,017)
2010	19.0% (3,604)	18.6% (6,583)
2011	23.8% (4,519)	22.5% (7,946)
2012	22.7% (4,304)	22.1% (7,826)

## Results

Figure 1 presents the elderly CVD admissions (hollow circles) and daily average temperature (solid line) during the 5-year period and shows the seasonal variation of daily average temperature and the erratic variation of elderly CVD admissions. Therefore, the relationship between these two variables is not clear in this graph.

**Fig. 1:** Daily average temperature (line) and number of daily elderly admissions (hollow circles).

Using Pearson's correlation coefficients, the daily average temperature was strongly inversely correlated with daily average air pressure (*r*=−0.8561, *p*<0.001), and was moderately or weakly positively correlated with daily sunshine hours, daily average humidity, daily evaporation, and daily precipitation with correlation coefficients amounting to 0.4948, 0.2538, 0.2314, 0.1360, respectively (*p*<0.001 for all four above correlations coefficients). Spearman's rank correlation coefficients were used to measure the crude relationship of each pair of temperature, air pressure, evaporation, humidity, precipitation, and hospital admitted cases allowing for potential non-linearities. The correlation coefficients are presented in Table 3 and show a positive correlation of the temperature and negative correlation of daily average air pressure to the elderly CVD admissions. All of these correlations were significant with p-values less than 0.001. Daily evaporation, daily average humidity, and daily precipitation did not show a significant correlation with elderly CVD admissions.

**Table 3:** Spearman's rank correlation between the numbers of elderly CVD admissions and daily weather variability in Thai Nguyen province

	Spearman's rank correlation of elderly CVD cases
Minimum temperature	0.1043*
Average temperature	0.0918*
Maximum temperature	0.0863*
Average air pressure	−0.0810*
Evaporation	0.0157
Average humidity	0.0084
Precipitation	−0.0017

* *p*<0.001.

By using a DLNM with a linear threshold model for temperature, it was found that the exposure–response relationship for CVD admissions was described by a ‘V’ shape with a threshold temperature (of minimum CVD admissions) of 26°C. This relationship was adjusted for other time-varying factors (Fig. 2).

**Fig. 2:** The estimated covariate adjusted effect (relative risk, RR) of daily average temperature on elderly cardiovascular admissions (adjusted to time trends with natural cubic splines, day of the week, and public holidays). The graph shows that the average point of minimum cardiovascular diseases (CVD) admissions was at 26°C. Above and below this temperature, cumulative CVD admission risk over 30 lag days tended to increase.

The cold effect was found significant while the heat effect was found to be non-significant. The overall effect of cold exposure on CVD admissions over lag 0–30 was estimated at a relative risk of 1.12 (95% confident interval: 1.01–1.25) for 1°C decrease below the threshold. The overall effect of heat exposure on CVD admissions was estimated at a non-significant relative risk of 1.17 (95% confidence interval: 0.90–1.52) for 1°C increase above the threshold (Fig. 3).

**Fig. 3:** The 30 days’ cumulative relative risk of cardiovascular diseases admission at different daily average temperature. Reference at 26°C. The 95% confident intervals are reported as shaded areas.

In Figure 4, the left graphs from top present the relative risk by temperature at selected lags 0, 4, 7, 14, and 30 (the relationship for all lags modeled are presented in Fig. 2). Low temperature did not have a clear effect at lag 0 but significantly increased CVD admission risk at lag 4, lag 7 (most significant), and lag 14, whereas high temperature reduced CVD admission risk at lag 0 and increased the risk of CVD admission at lag 4, lag 7, and lag 14 (but not significant). The effects of both low temperature and high temperature on CVD admissions became negligible at lag 30.

**Fig. 4:** Relative risk (RR) of cardiovascular diseases (CVD) admissions by temperature, which is denoted as Var, and lag using cross-basis smoothing. Left from top: RR by temperature at selected lags 0, 4, 7, 14, and 30°C. Right from top: RR by lag at selected temperatures 13, 20, 28, and 30°C. Risk of CVD admission is scaled to be relative to that at 26°C, the temperature that is sustained so that the CVD admission would be the lowest. Reference at 26°C. The 95% confident intervals are reported as shaded areas.

In Figure 4, the right graphs from top present the relative risk by lag at selected temperatures 13, 20, 28, and 30°C. From these graphs, one can see that the effect of low temperature varies by lag time. Cold effect was found to generally occur 4–15 days following exposure, peaking at a week's delay. However, it is clear that overall, low temperatures increase the risk of CVD. The effect of the more extreme temperature (13°C) was more pronounced on CVD admissions than that of less extreme temperature (20°C). The effect of temperature at 25°C on CVD admissions is almost identical to that at 26°C, which is the reference temperature. The effect of temperature of 30°C which is higher than the reference temperature reduced the risk of CVD admissions at lag 0 and increased the risk of CVD admissions from lag 5 to lag 10. However, the overall effect of high temperature is not as pronounced as that of low temperature. The right graphs of Fig. 4 also show that the effects of both low temperature and high temperature on CVD admissions became negligible at lag 30.

Using DLNM, we could not find any association of daily sunshine time, precipitation, or humidity on the elderly CVD admissions (results not shown).

We observed a significant effect of day of the week and public holidays in the elderly CVD admissions. During public holidays, the elderly CVD admissions is reduced by 50%. Weekday patterns revealed that compared to Friday, the admissions on Saturday and Sunday decreased but the admissions on Monday, Tuesday, Wednesday, and Thursday increased.

## Discussions

The goal of this study was to estimate the effects of the weather parameter during the years 2008 to 2012 on elderly CVD hospital admissions in the Thai Nguyen province. This study shows that the average point of minimum CVD admissions was at 26°C. Above and below this threshold, cumulative CVD admission risk over 30 lag days tended to increase with both lower and higher temperatures. Cold effect was found to generally occur 4–15 days following exposure, peaking at a week's delay. The cumulative effect of cold exposure on CVD admission was statistically significant at a relative risk of 1.12 (95% confidence interval: 1.01–1.25) for every 1°C decrease below the threshold of 26°C. The cumulative effect of hot temperature on CVD admission was found to be non-significant and estimating a relative risk of 1.17 (95% confidence interval: 0.90–1.52) for 1°C increase in temperature. No significant association was found between CVD admissions and the other weather variables.

The effect of low temperature on CVD events can be explained by pathophysiology and have been well documented by several studies. At low temperature, the blood vessels become narrow and the blood pressure increase. Cold temperature could lead to thrombosis, and physical activity during cold weather can increase the risk of stable angina and acute coronary syndrome (11, 24). Several studies have also shown that cold temperature increases the risk of CVD events (9, 10, 20, 24, 36). However, the effect of low temperature on CVD admissions found in this study occurs at higher temperature thresholds and are of greater magnitude than the effect found in other studies. This supports the hypothesis that cold effect in a warmer climate, where people usually do not have good houses to protect them from the cold weather, appears to be severe than that in a colder climate (10, 12). The cold effect found in this study generally occurred 4–15 days following exposure, peaking at a week's delay, and is consistent with the results of delayed cold effect found in other studies (17, 36, 37).

In this study, we could not find a significant cumulative heat effect on CVD admission as in some other studies (11, 22). These results are, however, derived for admission and the situation may look different for mortality. For admissions, we observed a potentially large cumulative estimate amounting to 17% increase in mortality per degree increase in heat exposure on warm days. The less strong signal of high temperature on elderly in Thai Nguyen province suggests that the adaptation of people living in warmer climates, through physical adaptation, special housing characteristics or behavioral patterns, may make them less affected by effects of warm temperature. This finding is consistent with the finding from a study based on populations in a hot desert climate (21), and in general when comparing cities with a warmer climate to cities with a cooler climate it appears the heat association is weaker in climatologically warmer areas (9). Another explanation is that, in this study, hypertension is the most common cause of CVD admissions, and cold temperatures increase the blood pressure, whereas the effect of hot temperatures reduces the blood pressure.

Although the cumulative effect of high temperature increases the risk of CVD admissions, one intriguing result in this study is that high temperatures reduced the CVD admissions at lag 0. This reduction counters studies on the effects of hot temperature on mortality (12, 18, 28, 36) and on hospital admission (11), where the effect of increasing risk starts at lag 0. Therefore, this result has to be confirmed or refuted by more studies carried out in developing, tropical countries. However, this may be explained by the fact that a high proportion of CVD admissions are for hypertensive disorders, whereas at high temperatures, the blood pressure will be reduced by decreased sympathetic tone and by dilated blood vessels (38, 39). Non-significant increase in hospital admissions at lag 5 may be due to the harvesting effect (25). The results also indicate a presumed behavioral adaptation to the prevailing weather conditions with respect to health-seeking behavior. Likewise, higher mortality rates in Europe during extreme heat waves are suggested to be partly due to people avoiding the bad weather and staying indoors instead of seeking medical care (2, 40). It is important to study mortality rates in relationship to admissions to examine if the decrease in admissions further increases the associated mortality rates.

In conclusion, the relationship between daily average temperature and elderly CVD admissions found in this study can be described as a ‘V’ shape with an optimal temperature (the average point of minimum CVD admissions) of 26°C, which is higher than the threshold found in studies in temperate climate. This pattern is consistent with the adaptation hypothesis.

## References

[1] McMichael AJ (2013). Globalization, climate change, and human health. N Engl J Med.

[2] Rocklöv J, Forsberg B (2009). Comparing approaches for studying the effects of climate extremes – a case study of hospital admissions in Sweden during an extremely warm summer. Glob Health Action.

[3] Richardson K, Steffen W, Schellnhuber HJ, Alcamo J, Barker T, Kammen DM (2009). Synthesis report on climate change: global risks, challenges and decisions.

[4] Stern N (2007). The economics of climate change – the stern review.

[5] Shuman EK (2010). Global climate change and infectious diseases. Perspective. N Engl J Med.

[6] Aström C, Rocklöv J, Hales S, Béguin A, Louis V, Sauerborn R (2012). Potential distribution of dengue fever under scenarios of climate change and economic development. Ecohealth.

[7] Lyons C, Coetzee M, Chown S (2013). Stable and fluctuating temperature effects on the development rate and survival of two malaria vectors, Anopheles arabiensis and Anopheles funestus. Parasit Vectors.

[8] Morand S, Owers KA, Waret-Szkuta A, McIntyre KM, Baylis M (2013). Climate variability and outbreaks of infectious diseases in Europe. Sci Rep.

[9] McMichael AJ, Wilkinson P, Kovats RS, Pattenden S, Hajat S, Armstrong B (2008). International study of temperature, heat and urban mortality: the ‘ISOTHURM’ project. Int J Epidemiol.

[10] Barnett AG, Dobson AJ, McElduff P, Salomaa V, Kuulasmaa K, Sans S (2005). Cold periods and coronary events: an analysis of populations worldwide. J Epidemiol Community Health.

[11] Schwartz J, Samet JM, Patz JA (2004). Hospital admissions for heart disease the effects of temperature and humidity. Epidemiology.

[12] Anderson BG, Bell ML (2009). Weather-related mortality: how heat, cold, and heat waves affect mortality in the United States. Epidemiology.

[13] Goldberg MS, Gasparrini A, Armstrong B, Valois MF (2011). The short-term influence of temperature on daily mortality in the temperate climate of Montreal, Canada. Environ Res.

[14] Mastrangelo G, Fedeli U, Visentin C, Milan G, Fadda E, Spolaore P (2007). Pattern and determinants of hospitalization during heat waves: an ecologic study. BMC Public Health.

[15] Belmin J, Auffray J-C, Berbezier C, Boirin P, Mercier S, Revier Bed (2007). Level of dependency: a simple marker associated with mortality during the 2003 heatwave among French dependent elderly people living in the community or in institutions. Age Ageing.

[16] Huang W, Kan H, Kovats S (2010). The impact of the 2003 heat wave on mortality in Shanghai, China. Sci Total Environ.

[17] Goggins WB, Chan EY, Yang C, Chong M (2013). Associations between mortality and meteorological and pollutant variables during the cool season in two Asian cities with sub-tropical climates: Hong Kong and Taipei. Environ Health.

[18] Nitschke M, Tucker GR, Bi P (2007). Morbidity and mortality during heatwaves in metropolitan Adelaide. Med J Aust.

[19] Wang XY, Barnett AG, Yu W, FitzGerald G, Tippett V, Aitken P (2011). The impact of heatwaves on mortality and emergency hospital admissions from non-external causes in Brisbane, Australia. Occup Environ Med.

[20] Turner LR, Connell D, Tong S (2010). Exposure to hot and cold temperatures and ambulance attendances in Brisbane, Australia: a time-series study. BMJ Open.

[21] Farajzadeh M, Darand M (2009). Analyzing the influence of air temperature on cardiovascular, respiratory and stroke mortality in Tehran, Iran. J Environ Health Sci Eng.

[22] Ebi K, Exuzides K, Lau E, Kelsh M, Barnston A (2004). Weather changes associated with hospitalizations for cardiovascular diseases and stroke in California, 1983–1998. Int J Biometeorol.

[23] Ohshige K, Hori Y, Tochikubo O, Sugiyama M (2006). Influence of weather on emergency transport events coded as stroke: population-based study in Japan. Int J Biometeorol.

[24] The Eurowinter Group (1997). Cold exposure and winter mortality from ischaemic heart disease, cerebrovascular disease, respiratory disease, and all causes in warm and cold regions of Europe. The Eurowinter Group. Lancet.

[25] Greenberg JH, Bromberg J, Reed CM, Gustafson TL, Beauchamp RA (1983). The epidemiology of heat-related deaths, Texas–1950, 1970–79, and 1980. Am J Public Health.

[26] Rowell LB (1974). Human cardiovascular adjustments to exercise and thermal stress. Physiol Rev.

[27] Kenney WL, Munce TA (2003). Invited review: aging and human temperature regulation. J Appl Physiol (1985).

[28] Basu R, Samet JM (2002). Relation between elevated ambient temperature and mortality: a review of the epidemiologic evidence. Epidemiol Rev.

[29] (2011). Vietnam Consultative Group Meeting. Vietnam development report: Market economy for a middle-income Vietnam.

[30] Tu NTH, Huong NTL, Diep NB (2004). Globalization and its effects on health care and occupational health in Vietnam.

[31] Institute of Strategy and Policy on Natural Resources and Environment (2009). Vietnam assessment report on climate change.

[32] Canty and Associates LLC (2014). http://www.weatherbase.com/weather/weather.php3?s=13884&cityname=Thai-Nguyen-Thai-Nguyen-Vietnam.

[33] General Statistics Organization of Vietnam (2009). Main results of National Census in 2009.

[34] Vietnam Assembly (2000). The Ordinance of Elderly of Vietnam No 23/2000/PL-UBTVQH dated 28/04/2000.

[35] Gasparrini A (2011). Distributed Lag Linear and Non-Linear Models in R: The Package dlnm. J Stat Softw.

[36] Armstrong B (2006). Models for the relationship between ambient temperature and daily mortality. Epidemiology.

[37] Gasparrini A, Armstrong B (2013). Reducing and meta-analysing estimates from distributed lag non-linear models. BMC Med Res Methodol.

[38] Jansen PM, Leineweber MJ, Thien T (2001). The effect of a change in ambient temperature on blood pressure in normotensives. J Hum Hypertens.

[39] Kunutsor SK, Powles JW (2010). The effect of ambient temperature on blood pressure in a rural West African adult population: a cross-sectional study. Cardiovasc J Afr.

[40] Kovats RS, Hajat S, Wilkinson P (2004). Contrasting patterns of mortality and hospital admissions during hot weather and heat waves in Greater London, UK. Occup Environ Med.

# Sub article: Diet and nutritional status among children 24–59 months by seasons in a mountainous area of Northern Vietnam in 2012

**DOI:** 10.3402/gha.v7.23121

**Published:** 2014-12-08

**Authors:** Le Thi Huong, Le Thi Thanh Xuan, Le Hong Phuong, Doan Thi Thu Huyen, Joacim Rocklöv

**Affiliations:** 1 Institute for Preventive Medicine and Public Health, Hanoi Medical University, Hanoi, Vietnam; 2 Epidemiology and Global Health, Department of Public Health and Clinical Medicine, Umeå University, Umeå, Sweden

**Keywords:** Vietnam, nutritional status, diet, children, Tuyen Quang, seasons

## Abstract

**Background:**

Seasonal variation affects food availability. However, it is not clear if it affects dietary intake and nutritional status of children in Vietnam.

**Objectives:**

This paper aims at examining the seasonal variation in nutrition status and dietary intake of children aged 24–59 months.

**Design:**

A repeated cross-sectional study design was used to collect data of changes in nutritional status and diets of children from 24 to 59 months through four seasons in Chiem Hoa district, Tuyen Quang province, a predominately rural mountainous province of northern Vietnam. The quantitative component includes anthropometric measurements, 24 hours dietary recall and socio-economic characteristics. The qualitative component was conducted through focus group discussions (FGDs) with mothers of the children surveyed in the quantitative component. The purpose of FGDs was to explore the food habits of children during the different seasons and the behaviours of their mothers in relation to the food that they provide during these seasons.

**Results:**

The prevalence of underweight among children aged 24–59 months is estimated at around 20–25%; it peaked in summer (24.9%) and reached a low in winter (21.3%). The prevalence of stunting was highest in summer (29.8%) and lowest in winter (22.2%). The prevalence of wasting in children was higher in spring and autumn (14.3%) and lower in summer (9.3%). Energy intake of children was highest in the autumn (1259.3 kcal) and lowest in the summer (996.9 kcal). Most of the energy and the nutrient intakes during the four seasons did not meet the Vietnamese National Institute of Nutrition recommendation.

**Conclusions:**

Our study describes some seasonal variation in nutrition status and energy intake among children in a mountainous area northern Vietnam. Our study indicated that the prevalence of stunting and underweight was higher in summer and autumn, while the prevalence of wasting was higher in spring and autumn. Energy intake did not always meet national recommendations, especially in summer.

Nutrition is very important for the growth and development of children (1). Child development depends on many factors such as: genetic, endocrine, autonomic nervous system and nutritional status (1). The first three factors certainly play important roles for cognitive development of children, while proper nutrition supplies necessary substances for maximisation of the potential development (1).

Results from National Nutrition Surveillance, from 2000 to 2009, showed that malnutrition among children aged less than 5 years in Vietnam (especially severe malnutrition and underweight) has reduced significantly and sustainably. However, the prevalence of underweight among children aged less than 5 years in some mountainous areas was up to 25–35% and stunting prevalence up to 37–47% (2–4). While in the urban areas, the prevalence of underweight was around 10% and prevalence of stunting was around 20% (5).

Xuan Quang is an agriculture area where subsistence farming is practiced. It is assumed that differences of weather through seasons may have an impact on household food availability and food consumption, which affects the child's nutritional status (6–9). Few studies have examined the effect of different season on nutrition status of children (10–13). The aims of this study are: 1) to describe the seasonal change of children's diet in Xuan Quang commune, Chiem Hoa district, Tuyen Quang province; 2) to describe the seasonal change of nutrition status of children from 24 to 59 months in Xuan Quang commune; 3) to explore the food habits of children and behaviours of the mothers in relation to the food that they provide to their children during these different seasons. This study would help develop strategies to protect vulnerable populations to overcome the potential negative effects of seasonal variability in climate.

## Methods

### Study setting

Xuan Quang is located in a rural mountainous area in the North of Chiem Hoa district where the climate varies by season. The commune has 13 villages with 10 groups of ethnic minority people (78.3%), while the Kinh group contains 11.7% of the population. The number of households in Xuan Quang was 1,192, of which 234 and 331 households were poor and near to poor, respectively (following the ranking of the local authority). Based on the commune health centre report in 2011, the commune had 5,000 people, of them about 200 children aged 24–59 months (14).

There are four seasons in Tuyen Quang province: spring, summer, autumn and winter. The dry and cold winter lasts from November to February. The spring lasts from March to April; rainy hot summer lasts from May to August. Autumn lasts from September to October. Autumn and spring are the two short transitional seasons. The annual rainfall average of the region is normally in the interval of 1,295–2,266 mm; the average temperature varies from 26 to 29°C (Fig. 1), and the annual humidity average is around 85%. Resulting from geographic location, Tuyen Quang province is divided into two regions with different weather conditions; the northern part of this province, including Xuan Quang commune, where this study is carried out, has a longer winter, lower temperature and much more rainfall in summer (15).

**Fig. 1:** Average temperature of Tuyen Quang province in 2012 (15).

### Study subjects

#### Quantitative study

All children 24–59 months living in Xuan Quang commune of Chiem Hoa district without congenital, chronic or acute diseases were included in each survey together with their mothers. We selected this group because between the ages of 2–5, children start eating with their family and are less likely to receive special diets.

#### Qualitative study

We invited mothers who have children from 24 to 59 months (6–8 mothers for each focus group discussion).

Mothers/caregivers were informed about the purposes of the survey, and asked to sign an informed consent before participating in the study.

### Study design and data collection

A repeated cross-sectional study design was used to collect data of seasonal changes in nutrition status and nutrients intake of children from 24 to 59 months. Data were collected at the beginning of spring (March), summer (June), autumn (September) and winter (December) (15).

A list of mothers and children was provided by village health workers. The mothers and children were invited by the village health workers to the kindergarten, village meeting room or community health centre for the anthropometric measurements and the 24-hour recall interviews. The mothers that participated in the FGDs were invited to a separate room for these sessions.

#### Anthropometric measurement

All children aged 24–59 months were measured for weight and height by project researchers with support from village health workers. We used Microtoise height meter for measuring children's height with the precision of 0.1 cm. Weight was measured by electronic Nhon Hoa scales with the precision of 100 g. All children were undressed for weighing. In each survey, the well-trained staff from the Nutrition Department at Hanoi Medical University conducted one measurement for each child.

#### Nutrition status identification

Three indicators were used to identify the nutritional status of the children, using the WHO recommendation applied in Vietnam since 2006: weight for age, height for age and weight for height (16–18). Underweight was defined as: weight for age z-score (WAZ) ≤2; stunting was defined as: height for age z-score (HAZ) ≤2; and wasting was defined as: weight for height z-score (WHZ) ≤2 (19). All children of the commune aged 24–59 months were included in the anthropometric measurement at each study time (spring: 195 children; summer: 237 children; autumn: 196 children and winter: 225 children).

#### Food intake of children (24 hours recall)

To be able to assure a large enough sample size to perform valid statistical tests with a power of 80% and a significance level of 5%, we performed sample size calculations with a standard *t*-test. The sample size calculations were subject to the outcome of food intake of children:

Sample size was calculated based on the following formula (20):

$$N=\frac{t^{2}.\delta^{\text{2}}.n}{e^{2}.n+t^{2}.\delta^{\text{2}}}$$

where:

*N*: Sample size (people)

*t*: The standard percentile (=2 with a probability of 0.954)

δ: Standard Deviation (base on previous study estimated to 400 kcal)

*n*: Total number of children from 24 to 59 months of population

*e*: Relative precision (70 kcal was selected)

The sample size was calculated to include 79 children. An additional 10% was added to account for children who may not completed the survey. This resulted in a sample of 90 children for 24-hour recall in each survey.

Based on the list of children aged 24–59 months of the commune that was provided by the commune health staff, 90 children and their mothers were then randomly selected for each season. To make the balance for three age groups: 24–35 months, 36–47 months and 48–59 months, we randomly selected 30 children from each age group.

The 24-hour recall questionnaire was designed to collect data in the four seasons. One day 24-hour recall was collected in normal day for each season. The face-to-face interview was conducted by the well-trained staff from Nutrition Department at Hanoi Medical University. The researchers were equipped with instruments like food pictures, various kinds of spoons and mugs to help the mothers estimate the amount of food provided to children during the day. In this study area, the mothers were the main caregivers of children, who prepared foods and fed the children. In case a mother could not feed the children during the day, their grandfather or grandmother usually fed the children using food prepared by the mother.

#### Focus group discussion

Two focus group discussions (FGDs) (6–8 mothers) were applied during the first round of this study. The main focus of the FGDs was to explore the mother's view on their children's food habits depending on season. This discussion also focused on exploring the mother's behaviours regarding food selection and preparation for children through these seasons. The FGD was conducted by researchers with experience in qualitative methods to ensure quality of the data (21).

Mothers/caregivers were randomly selected and invited to a community health centre for the FGDs. The FGD was conducted in a separate and private room to make mothers and researchers feel comfortable. The guideline was developed to facilitate the FGDs and to create a positive interview atmosphere, where the informants could share the experiences. Each focus group discussion lasted for about 45 min. It was facilitated by one researcher with the support from another researcher responsible for taking notes.

### Data analysis

Anthropometric data was entered in Anthro software version 2005 and analysed using STATA version 10.0 (22). Data of 24-hour recalls were entered and analysed in Access office package for nutrition analyses. A module in Access had been developed by Vietnam National Institute of Nutrition (NIN).

The ranksum-test was used to test for significant difference between the medians of two groups and Chi-square test was used to test for significant differences between the proportions of groups. A *p*-value under 0.05 was considered statistically significant. Kruskal-Wallis test was used to test for significant difference in the medians of more than two groups.

Qualitative data were analysed by using a content analysis method. The information was coded following themes, and the analysis was carried out by the researcher conducting the FGD. More specifically, the researcher read the notes, arranged the content following the topic, coded, analysed into themes, and presented integrated with quantitative data (23).

### Ethical considerations

The protocol of this study was approved by the Scientific and Ethical Committee in Biomedical Research, Hanoi Medical University. All subjects in the study were asked for their informed consent before collecting data, and all had complete rights to withdraw from the study at any time without any threats or disadvantages.

## Results

The number of children involved in the anthropometric measurement during the spring, summer, autumn and winter were 195, 237, 196 and 225, respectively.

This study did not show a large difference in mean weight and height of children between seasons. The mean weight varied from 12.4 to 12.7 kg and the mean the height varied from 92.4 to 93.1 cm. There was no significant difference between the seasons on the mean WAZ, HAZ and WHZ of the children (Table 1).

**Table 1:** Mean weight, height and z-score of the children following the seasons

	Mean	SD	*p*
Weight			
Spring	12.7	2,1	*p*>0.05
Summer	12.6	2,0	
Autumn	12.7	2,2	
Winter	12.4	1,9	
Height			
Spring	93.1	8.1	*p*>0.05
Summer	93.0	7.8	
Autumn	92.6	8.0	
Winter	92.4	8.3	
WAZ (weight for age z-score)			
Spring	−1.4	0.9	*p*>0.05
Summer	−1.4	0.9	
Autumn	−1.4	1.0	
Winter	−1.4	0.9	
HAZ (height for age z-score)			
Spring	−1.5	1.2	*p*>0.05
Summer	−1.5	1.2	
Autumn	−1.5	1.2	
Winter	−1.5	1.2	
WHZ (weight for height z-score)			
Spring	−0.8	1.1	*p*>0.05
Summer	−0.8	1.1	
Autumn	−0.8	1.2	
Winter	−0.9	1.1	

Figure 2 presents the seasonal changes in the prevalence of children's malnutrition. The prevalence of stunting was around 20–30%, highest in summer (29.8%) and lowest in winter (22.2%). There was no significant difference on stunting prevalence between age groups (24–35 months, 36–47 months and 48–59 months). The proportion of underweight was lowest in winter (21.3%) and ranging from 23 to 24% in the other seasons with no statistical significant difference (Chi-squared test, *p*>0.05). By contrast, the proportion of wasting children was higher in spring and autumn (14.3%) and lower in summer (9.3%). There was no statistical significant difference among seasons (*p*>0.05).

**Fig. 2:** Prevalence of Underweight, Stunting and Wasting by season.

The results of the focus group discussion indicated that the main food source supplying energy intake for children in this commune was rice. Foods rich in protein available to access for local people in all seasons were pork, fish, eggs and bean. Crabs, eels, snails and mussels are available mainly in the summer. However, some nutritious foods were not fed to children because mothers did not know how to prepare these food items or they were afraid that their children risk having gastrointestinal problems after eating these.

My neighbour *don't know how to cook crabs, she doesn't know what the nutrient value of crabs* are. Many mothers are afraid that when their child *eat crab, they may have* gastrointestinal problems. (Mother, 28 years old, Xuan Quang. Mentioned during the FGD)

The data from FGD also indicated seasonal variation in the availability of vegetable and fruit, but not for protein-rich foods. During winter, more green leaf vegetables are being consumed compared to other seasons.

Table 2 showed a significant seasonal variation in total energy intake (*p*<0.01): highest in autumn (1259.3 kcal), lower in spring and winter and lowest in summer (996.9 kcal). In addition, the amount intake of carbohydrates protein and lipids among children in autumn were higher than other seasons (*p*<0.05). Most of the children's diets did not meet the recommendation of the NIN for energy, protein, carbohydrates and lipid intake.

**Table 2:** Energy, protein, glucid and lipid intake of children aged 24–59 months by season

Age group (*n*=90)	Recommendation	Spring (*n*=90)	Summer (*n*=90)	Autumn (*n*=90)	Winter (*n*=90)	(Kruskal-Wallis test)
Energy (kcal/day)						
24–35 months	*1,180*	1041.3	991.7	1194.2	1008.7	>0.05
36–47 months	*1,180*	1049.6	957.9	1247.3	1166.1	**<0.05**
48–59 months	*1,470*	1103.7	1053.9	1327.7	1053.6	**<0.05**
All		1067.8	996.9	1259.3	1075.4	**<0.01**
Protein (g/day)						
24–35 months	*35–44*	39.3	40.7	48.2	40.2	>0.05
36–47 months	*35–44*	43.9	40.2	51.5	42.8	>0.05
48–59 months	*44–55*	40.7	44.7	54.4	38.3	**<0.01**
All		41.1	41.4	51.5	40.4	**<0.01**
Lipid (%=lipid energy demand/total energy)						
24–35 months	*44–51*	18.4	23.5	28.1	24.3	**<0.05**
36–47 months	*44–51*	23.6	19.0	29.5	24.4	>0.05
48–59 months	*32–40*	20.1	22.5	30.6	20.1	**<0.05**
All		20.5	22.2	29.5	22.8	**<0.01**
Glucid (g/day)						
24–35 months	*175–202*	178.3	153.4	187.3	157.8	**<0.05**
36–47 months	*175–202*	164.2	157.0	192.9	194.4	**<0.05**
48–59 months	*219–251*	189.4	166.6	206.9	180.5	**<0.05**
All		178.8	157.0	196.1	177.7	**<0.01**

FGD with mothers in the local study area showed a similar result with mothers indicating that children eat more in the wintertime:

It also depends on the weather condition, for example, in autumn the children often eat better whereas in hot conditions of summer children prefer to eat fruits rather than main diets. (Mother, 28 years old, Xuan Quang. Mentioned during the FGD)

Figure 3 presents the proportion of total energy intake in diets of children by season. The results show that glucid is the main macronutrient for energy supplement, and represented more than 60% in all seasons, highest in spring (67.2%) and in winter (66%).

*In the summer*, the *kids will eat more junk food*, the number of *main meals is still the same but the child eats less*. *Mainly because of the weather*. The *hot* weather makes children *tired and as a consequence they* do not eat much. (Mother, 28 years, Xuan Quang. Mentioned during the FGD)

Energy supplement from protein was 14%, which was lower in spring and winter. There was no statistical significant difference of supplement balance among protein (P), glucid (G) and lipid (L) between seasons (*p*>0.05). In general, the proportion of the energy intake from nutrients intake (P:L:G) is similar to the recommendation (P:L:G=14:20:66).

**Fig. 3:** Proportion of substances supplying total energy in diets of children aged 24–59 months by season.

Findings from group discussion supported that children often eat more protein-rich foods in the autumn:

In the autumn, they eat more than the pre-harvest period. There is no fasting in pre-harvest period, mother do not want to buy meat or fish because they do not have money. (Mother, 28 years old, Xuan Quang. Mentioned during the FGD)

A difficulty identified through the FGD was that mothers find it harder to feed their children with protein-rich food because of inaccessibility, particularly, in rainy season when it can be very difficult to get to the market. Another reason that many mothers mentioned is the poor economic condition, and the lack of money to buy good foods such as beef, milk, etc.

Table 3 presents seasonal intakes of some vitamins, minerals and micronutrients by age group of children. The amount of vitamin A intake for children followed the recommendation whereas the amount of iron and calcium are below the recommendations. Results also show that the intake of iron and calcium are higher in the summer and the autumn (*p*<0.05). The amount of phosphorus consumed met the requirement; the resulting Ca/P ratio is rather high with 1:1.2 as the lowest level.

**Table 3:** Actual amount of vitamin and mineral intake by season and children age group

Age group (*n*=90)	Recommendation	Spring (*n*=90)	Summer (*n*=90)	Autumn (*n*=90)	Winter (*n*=90)	*p* (Kruskal-Wallis test)
Vitamin A (mcg/day)						
24–35 months	*400*	558.7	716.2	843.8	459.7	**<0.05**
36–47 months	*400*	519.1	687.4	780.3	486.5	>0.05
48–59 months	*450*	544.6	802.5	795.9	463.0	**<0.01**
All		542.7	769.9	**805.6**	469.5	**<0.01**
Calci (mg/day)						
24–35 months	*500*	346.2	537.0	433.4	394.8	>0.05
36–47 months	*500*	316.9	376.2	491.8	308.3	>0.05
48–59 months	*600*	300.2	381.0	403.7	269.8	**<0.01**
All		320.5	**466.6**	444.3	322.5	**<0.01**
Iron (mg/day)						
24–35 months	*7.7*	6.1	6.6	10.8	4.9	**<0.01**
36–47 months	*7.7*	5.5	6.4	6.9	6.9	**<0.05**
48–59 months	*8.4*	6.5	8.3	10.7	6.2	**<0.05**
All		6.1	6.9	**8.4**	6.0	**<0.01**
Ca/P						
Proportion Ca/P	*1.0:1.5*	1.0:1.7	1.0:1.2	1.0:1.5	1.0:1.7	

Table 4 indicated that malnourished children generally had a lower intake of glucid in spring and lower intake of lipid in summer (*p*<0.05). The table also shows that underweight children usually had a lower intake of energy, protein, carbohydrate and lipid than normal-weight children (*p*>0.05).

**Table 4:** Nutritional status (underweight) and dietary intake of children by season

	Spring (*n*=90)		Summer (*n*=90)		Autumn (*n*=90)		Winter (*n*=90)	
	
	Malnutrition	Non-malnutrition	Malnutrition	Non-malnutrition	Malnutrition	Non-malnutrition	Malnutrition	Non-malnutrition
Energy intake	981.4	1092.5	964	1008.2	1162.4	1290.7	1199.9	1050.5
*p* (ranksum-test)	>0.05		>0.05		>0.05		>0.05	
Protein	40.1	41.4	40.4	41.8	48.4	52.5	42.9	39.9
*p* (ranksum-test)	>0.05		>0.05		>0.05		>0.05	
Glucid	150.4	187.0	156.4	157.2	179.8	201.4	188.7	175.5
*p* (ranksum-test)	**<0.05**		>0.05		>0.05		>0.05	
Lipid	23.0	19.8	18.4	23.6	26.9	30.3	30.7	21.2
*p* (ranksum-test)	>0.05		**<0.05**		>0.05		**<0.05**	

## Discussion

The prevalence of underweight was highest in the summer (24.9%) and lowest in winter (21.3%). This level is much lower than the findings of another study in rural and mountainous areas carried out in Quan Son of Thanh Hoa province (about 30%) (24). The results showed that the proportion of stunting in Xuan Quang was highest in summer (29.8%), followed by autumn (29.1%), spring (27.7%) and winter (22.2%). The stunting rate, however, reflects the malnutrition status of children over a long time, while this study was carried out in 1 year. Therefore, the above results could not show the correlation between stunting prevalence and climatic extreme events and climate change. For such studies, it would be necessary to carry out longitudinal studies to examine the correlation between children malnutrition status and climate change over many years. However, this study may still be used as a reference if shifts in the seasonality of climatic extreme events would occur that would potentially effect the populations. Prevalence of stunting in our study was lower than that in a mountainous district of Thanh Hoa province (about 40%) (25). Our study found that wasting (the indicator that better reflects current acute malnutrition) prevalence ranged from 9.3 to 14.3%. Unlike underweight and stunting, the prevalence of wasting was highest in spring and autumn (14.3%), and lowest in summer (9.3%). This number was higher than the reported statistics of developing countries with 9% (26). A study of L. Loutan and J-M. Lamotte in Niger showed that a reduction of weight of children under 5 years from February to May caused a rapid increase of wasting from 7% in November to 17% in May (the peak time of the dry season is from February to May in Nigeria) (11).

The result from our study showed that the prevalence of stunting and underweight was higher in summer time in comparison with other seasons. A significant difference was, however, only observed for wasting in the summer time. In the study from Nigeria, children's weight did not increase in dry season but reduced by 113 g. After the dry season the children's weight increased again (11). Our study's results could not reflect the acute malnutrition of children caused by lower energy intake in summer due to time limitations of our study. The anthropometric data were collected at the beginning of the four seasons, so that the low prevalence of the wasting (acute malnutrition) at the beginning of summer (9.3%) can be explained by better food intake during the spring time, then the higher prevalence of wasting at the beginning of autumn (14.3%) as a result of poor nutrition intake during summer time. Moreover, the nutritional status of children not only depends on the food intake but also on others factors such as infectious illness, children caring, among other factors.

Although there was some significant seasonal variation in Xuan Quang commune in nutritional status, seasonality did, in general, not affect the production and access to food. Diets of the children were adapted to the circumstances, and mothers tried to get enough food per each four food-type group in each meal of the children. It is likely due to this adaptation that we observed that the prevalence of underweight and malnutrition did not differ by seasons. In comparison with Quan Son district of Thanh Hoa province, where a similar pattern was observed, this study area had poorer economic conditions and more seasonally contrasting weather patterns (24).

There was a significant seasonal difference in nutrition intake of children aged 24–59 months (*p*<0.01). The average energy intake, amount of proteins, lipid, glucid intake of children were all highest in autumn (*p*<0.01). The results from qualitative section explained that cool weather in autumn made children eating more than other seasons. Nutrition intake of children in all four seasons mostly failed to reach the recommendation for energy and nutrients. This result was similar with the result of NIN studies showing poor density of energy intake, fatty food, animal fatty and micronutrients (27). This finding resulted from the mothers work burden, especially in rural and mountainous areas, where they have less time to feed their children directly. In addition, results from group discussions showed the lack of mother's knowledge on caring and requirements of children's nutrition:

It's because of mothers’ knowledge. For example, they do not know how to prepare food from crabs as well as nutrients form crabs. Some children do not eat food from crabs, some have digestion problems. (Mother, 27 years old, Xuan Quang. Mentioned during a FGD)

The structure of the energy intake among children aged 24–59 months was similar in the four seasons. Glucid was the main substance supplying energy in all seasons with more than 60% (highest in spring and winter with 67 and 66%, respectively), which exceeded the recommendation (66%). Energy supplement from protein was higher than the recommendation's balance, ranging from 15 to 16.7%. This is consistent with the study by Le Thi Hop et al. (15.4%), which also showed an improvement in children's diets (28).

Iron is also an important and essential micronutrient for the human body, especially for young children. The amount of iron intake of children in our study did not meet the recommendation. Significantly higher amounts of iron intake were found in autumn and summer than spring and winter. Underweight children usually had lower energy intake, protein, glucid and lipid than children who were not underweight, where no significant difference was found. This result was different from another study finding that malnourished children had lower energy consumption, lower energy intake from animal protein and lipids than normal children (29); therefore, the higher prevalence of malnutrition may combine with the high prevalence of iron deficiency.

Our study found no differences in energy intake between stunting, wasting children and normal children in all four seasons (*p*>0.05). The sample size of this study may, however, not have been big enough to show a statistical significant difference for the differences observed. It is necessary to conduct a longer-term prospective study to find out the correlation between children's diets and the prevalence of malnutrition in relation to climate variability in the future.

## Conclusion

Our study shows some seasonal variation in nutrition status and energy intake. Prevalence of stunting and underweight were observed to be higher in summer and autumn, while the prevalence of wasting was observed to be higher in spring and autumn. Energy intakes did not always meet the recommendation, especially in summer.

## References

[1] Hop LT, Khoi HH (2009). Nutrition and growth. J Food Nutr Sci.

[2] Hop LT, Khoi HH (2010). Secular trend in growth of Vietnamese people and the orientation of national nutrition strategy period 2011–2020. J Food Nutr Sci.

[3] National Institute of Nutrition (2010). The trend of decreasing in malnutrition in mother and children less than 5 years old from 2000 to 2009.

[4] National Institute of Nutrition (2008) Children malnutrition results from provinces surveillance 2007.

[5] Do TT, Tuyen LD, Hoa NP (2012). The trend of changing mean value of Z-score in evaluation nutrition status of children from 2003–2011. J Food Nutr Sci.

[6] Food Agriculture Organization (2002) The state of food insecurity in the world 2001.

[7] Food Agriculture Organization (2006) The state of food insecurity in the world 2006.

[8] Choudhury AY, Bhuiya A (1995). Effects of biosocial variables on changes in nutritional status of rural Bangladeshi children, pre- and post-monsoon flooding. J Biosoc Sci.

[9] Singh MB, Fotedar R, Lakshminarayana J, Anand PK (2006). Studies on the nutritional status of children aged 0–5 years in a drought-affected desert area of western Rajasthan, India. Public Health Nutr.

[10] Baertl JM, Morales E, Verastegui G, Graham GG (1970). Diet supplementation for entire communities. Growth and mortality of infants and children. Am J Clin Nutr.

[11] Loutan L, Lamotte J-M (1984). Seasonal variations in nutrition among a group of nomadic pastoralists in Niger. Lancet.

[12] Ferguson EL (1993). Seasonal food consumption patterns and dietary diversity of rural preschool Ghanaian and Malawian children. Ecol Food Nutr.

[13] Zhang J, Shi L, Wang J, Wang Y (2009). An infant and child feeding index is associated with child nutritional status in rural China. Early Hum Dev.

[14] Tuyen Quang (2011). Commune Health center report of Xuan Quang, Chiem Hoa, Tuyen Quang, Vietnam.

[15] Tuyen Quang Hydro meteorological Department of Tuyen Quang report, Vietnam.

[16] Ministry of Health- National Institute of Nutrition (1998). Guideline for nutritional and dietary assessment in the community.

[17] WHO (1995). Physical status: the use and interpretation of anthropometry.

[18] WHO (2006). WHO Child Growth Standards based on length/height, weight and age. Acta Paediatrica.

[19] WHO (2012). Nutrition Landscape Information System (NLIS) country profile indicators: interpretation guide.

[20] Khoi HH (1997). Method of Nutritional epidemiology. Medicine Publisher in Vietnam.

[21] Dahlgren L, Emmelin M, Winkvist A (2007). Qualitative Methodology for international Public Health.

[22] WHO (2011). WHO Anthro software for assessing growth and development of the world's children, version 3.2.2.

[23] Graneheim UH, Lundman B (2004). Qualitative content analysis in nursing research: concept, procedures and measures to achieve trustworthiness. Nurse Educ Today.

[24] Thuy TX, Phu PV, Son PT (2012). Nutrition status of children under 5 years old of Thai ethnic group in Quan Son district, Thanh Hoa province from 2007–2011. J Med Res.

[25] Huong LT (2009). Knowledge and practices on child feeding of mothers and children nutrition status in a mountainous district of Thanh Hoa province. J Prac Med.

[26] Rice AL, Sacco L, Hyder A (2000). Malnutrition as an underlying cause of childhood deaths associated with infectious diseases in developing countries. Bulletin of the World Health Organization.

[27] National Institute of Nutrition (2006). Action plan of children feeding from 2006–2010, Vietnam.

[28] Hop LT, Tuyen LD (2010). Analysis structure household diets in Vietnam from 1990 to 2010. J Med Res.

[29] Tam BTT, Chien HD (2003). Diets and Nutrition status of children under 5 years old of a population living on the boat in Phu Binh commune of Hue city. J Sci.

# Sub article: Seasonality in mortality and its relationship to temperature among the older population in Hanoi, Vietnam

**DOI:** 10.3402/gha.v7.23115

**Published:** 2014-12-08

**Authors:** Le Thi Thanh Xuan, Thaddaeus Egondi, Le Tran Ngoan, Do Thi Thanh Toan, Le Thi Huong

**Affiliations:** 1 Department of Environmental Health, Institute for Preventive Medicine and Public Health, Hanoi Medical University, Hanoi, Vietnam; 2 African Population and Health Research Center, Nairobi, Kenya; 3 Epidemiology and Global Health, Department of Public Health and Clinical Medicine, Umeå University, Umeå, Sweden; 4 Department of Occupational Health, Institute for Preventive Medicine and Public Health, Hanoi Medical University, Hanoi, Vietnam; 5 Department of Biostatistics and Medical Informatics, Institute for Preventive Medicine and Public Health, Hanoi Medical University, Hanoi, Vietnam; 6 Department of Nutrition and Food Safety, Institute for Preventive Medicine and Public Health, Hanoi Medical University, Hanoi, Vietnam

**Keywords:** temperature, mortality, older, Vietnam

## Abstract

**Background:**

Several studies have established a relationship between temperature and mortality. In particular, older populations have been shown to be vulnerable to temperature effects. However, little information exists on the temperature–mortality relationship in Vietnam.

**Objectives:**

This article aims to examine the monthly temperature–mortality relationship among older people in Hanoi, Vietnam, over the period between 2005 and 2010, and estimate seasonal patterns in mortality.

**Methods:**

We employed Generalized Additive Models, including smooth functions, to model the temperature–mortality relationships. A quasi-Poisson distribution was used to model overdispersion of death counts. Temporal trends, seasonality, and population size were adjusted for while estimating changes in monthly mortality over the study period. A cold month was defined as a month with a mean temperature below 19°C.

**Results:**

This study found that the high peak of mortality coincided with low temperatures in the month of February 2008, during which the mean temperature was the lowest in the whole study period. There was a significant relationship between mean monthly temperature and mortality among the older people (*p*<0.01). Overall, there was a significant decrease in the number of deaths in the year 2009 during the study period. There was a 21% increase in the number of deaths during the cold season compared to the warm season. The increase in mortality during the cold period was higher among females compared to males (female: IRR [incidence relative risk] =1.23; male: IRR=1.18).

**Conclusions:**

Cold temperatures substantially increased mortality among the older population in Hanoi, Vietnam, and there were gender differences. Necessary preventive measures are required to mitigate temperature effects with greater attention to vulnerable groups.

Globally, the older population is growing rapidly in developing countries, and this has increased attention toward understanding health risks among older people. Likewise, the older population in low- or middle-income countries is suspected to be at higher risk of death due to weather variations (1–10). The reason might be partly attributable to developing countries’ limited capacity to address health problems in general but also, more particularly, to a lack of health interventions targeting older populations in these countries. Older people also have a reduced ability to maintain a normal temperature of 37°C (11–13). Furthermore, evidence indicates that factors such as poor living conditions, lack of access to proper medical care systems, and lack of family and/or social support have been shown to be modifiers of temperature-related effects among older people (14). These factors are worse in most developing countries, and, therefore, the effect of temperature among older people is expected to be greater. However, little evidence exists on temperature-related impacts among older populations in low- and middle-income countries.

Recently, there has been a growing body of literature on the temperature–mortality relationship among older people in both developed (13, 15–23) and developing countries (2, 4, 5, 8, 9, 24–26). Most previous studies have investigated the association between temperature and mortality, and found significant association (27). Different temperature indicators have been used in previous studies to assess temperature-related mortality. The indicators considered in previous studies included mean temperature (2–4, 10, 25, 26, 28), diurnal temperature (29, 30), and ambient (22, 31) or apparent temperatures (7, 32, 33). All of these indicators are found to produce similar results on temperature-related mortality (10). The temperature–mortality relationship has been found to show U, V, or J shapes, with a U-shaped pattern being a common shape (10, 24). The observed shapes show that, given a particular temperature threshold, increases and decreases in temperature are associated with increases in mortality (2–4, 15–17, 20, 21, 23–25, 34), but the magnitude of heat-related effects seemed to be larger than that of cold effects within a global context (9). Similar observations have been made among older populations (28, 35). Establishing a temperature–mortality relationship is critical for weather-based early warnings and the identification of susceptible groups. In addition, knowledge on temperature effects would show the importance of mitigation and adaptation strategies aimed at reducing the temperature-related effects.

Numerous studies have also investigated seasonal mortality patterns and cold-related excess mortality in the populations living in temperate climate regions (36, 37). So far, there are few such studies from warmer or subtropical areas. Excess mortality during cold temperatures may be a result of such factors as housing conditions, health status and demography, seasonality in infectious disease and indoor crowding, and fuel type (37–40). Studies from Europe show less winter excess mortality in Scandinavian countries (around 10%) compared to Southern European countries (36, 37, 39). The observed difference in winter-related mortality is partly attributed to the adaptation of housing to cold outdoor conditions, which is much more developed in Northern Europe compared to Southern Europe.

Vietnam is one of the countries that experience a tropical and humid climate. Over the last five decades, the temperature has increased by 0.2°C (41). The average temperature is projected to increase by 2.3°C in 2100 in Vietnam (baseline period: 1980–1999), although the changes vary across the country (42, 43). A recent study found that the older population is one of the most vulnerable groups affected by temperature changes in urban areas (44). However, so far, little information exists on the association between mortality and temperature among older populations in Vietnam. The current article aims to examine the seasonality in mortality and to quantify temperature-related mortality among the older population in Hanoi, Vietnam, during the period between 2005 and 2010.

## Methods

### Study location

Hanoi is the capital of Vietnam; it is situated in the north and located at latitude 16°0′ N. It has a population of about 6.5 million people, with 12% aged 60 years and older (45). Hanoi experiences a climate in which summers are hot and humid, and winters are relatively cool and dry (46). The summer period is from May to September, during which the highest amount of rainfall is experienced (1,682 mm rainfall). The winter months from December to March are relatively dry, while light rains are experienced during spring (from 18.6 mm to 23.4 mm). The minimum temperature during winter reaches about 6–7°C with no chilly wind, while summer can get to a maximum of 38–40°C.

### Data collection

We obtained daily weather data from Lang Station on daily mean temperature, minimum and maximum temperature, relative humidity, and rainfall. This station is located at the urban area of Hanoi city. Mortality data were obtained from the Vietnam health system's commune-level health stations. The Vietnam health system consists of four administrative levels: central, provincial, district, and commune levels. There is a policy that every death in Vietnam should be registered at commune health centers. We collected aggregated individual data (for sex and age group) for all 69,690 registered deaths between 1 January 2005 and 31 December 2010 from all 27 commune health centers in Hanoi city. The number of deaths of people aged 60+ was 47,172 cases, representing 68% of the total deaths. The data collected included month of death, sex, age, and location of the diseased person. Therefore, the analysis was conducted on aggregated mortality data at a monthly time scale.

### Data analysis

Summary measures were generated for both weather and death counts (all deaths and deaths among older people). Spearman correlation analyses were conducted using STATA software (47) to assess the bivariate association between the counts of death among older people and temperature measures (minimum (min), maximum (max), and mean), as well as other weather indicators (rainfall and relative humidity). The daily mean temperature was used as a temperature indicator to assess the relationship between weather and mortality. The mean temperature indicator has been shown to produce similar effect estimates as compared to other temperature indicators (min, max, and range) (10).

Counts of monthly deaths and mean temperature were modeled using quasi-Poisson regression to account for overdispersion. The use of quasi-Poisson does not have an effect on the coefficient estimates of the model but adjusts standard errors for overdispersion. The temperature–mortality relationship was examined through Generalized Additive Models (GAMs) with the use of smooth functions. The model included nonlinear effects of weather variables, trend, and season. The model equation for the GAM is described here (48). The Akaike Information Criterion (AIC) was used to assess different models with lags up to three months, while smoothness parameters were determined using graphical visualization. The initial assessment did not show any delay effect of temperature, and therefore the final model did not include lag terms. Lack of lag effect could be because of the aggregation of mortality data at a monthly scale.

$$Y_{t}\sim Poisson\left(\mu_{t}\right)$$

$$\text{log}\left(\mu_{\text{t}}\right)=\alpha +s\left(time,df\right)+s\left(month,df\right)+s\left(tmean,df\right)$$

where *t* refers to the month of the observation; (*Y*_*t*_) denotes the observed monthly death counts during month *t*; *s(.)* denotes a smooth function; *df* represents degrees of freedom, or the smoothing parameter; *tmean* is the mean monthly temperature; and *time* and *month* represent both trend and seasonal terms, respectively. The GAM model was fitted using R statistical software (R Foundation for Statistical Computing, version 3.0).

To quantify the cold-season-related mortality among older people, a dummy variable was created to represent cold and warm seasons. At the initial inspection of the temperature seasonality, the cold season was identified to be during the months of December, January, February, and March with an average temperature of 18.9°C. Quasi-Poisson regression was used to estimate the relative risk related to cold weather using a calendar year as a factor variable. The interaction between season variable and year was checked to examine whether the season effect differed across the years. The interaction term was dropped from the model because it was insignificant, and the final model was:

$$\text{log}\left(\mu_{t}\right)=\beta_{0}+\sum_{i=1}^{5}\beta_{i}Year_{i}+\beta_{6}Season$$

where the year 2005 was taken as the baseline and the season is ‘1’ for cold months. Therefore, the exponential of *β*_6_ gives the relative risk of death among older people during the cold season compared to the warmer season. The model was also fitted for males and females separately. The total population of older people was used as an offset in all quasi-Poisson models. For all statistical tests, two-tailed tests were considered statistically significant with a *p*-value less than 0.05. All data manipulation was done in STATA, and statistical analyses were performed using the ‘mgcv’ functions of R packages.

## Results

Summary statistics for monthly weather conditions (temperature, humidity, and rainfall) and death counts are presented in Table 1. The monthly average estimates for mean, minimum, and maximum temperatures were 24.5°C, 22.0°C, and 28.4°C, respectively. Average relative humidity was 78% for the study period. The average mortality count for all-cause deaths among older people was 662 deaths (346 males and 316 females).

**Table 1:** Summary statistics for monthly weather and number of deaths in Hanoi, Vietnam, 2005–2010

		Percentiles				
		
	Mean (SD)	Min	P_25_	Median	P_75_	Max
Weather measures						
Mean monthly temperature (°C)	24.5 (4.7)	13.8	20.9	25.5	28.7	30.9
Min monthly temperature (°C)	22.0 (4.4)	12.1	18.6	23.1	26.1	27.8
Max monthly temperature (°C)	28.4 (5.1)	16.3	24.1	29.5	32.9	35.5
Rain fall (mm)	112.2 (132.3)	0.4	8.1	40.1	191.7	550.5
Relative humidity (%)	78.1 (44.8)	66.5	75.4	78.5	81	87.6
Wind speed	1.5 (0.2)	0.9	1.3	1.4	1.7	1.9
Monthly all-cause deaths						
Male	564.6 (104.5)	15	514	553	614	903
Female	388.3 (84.2)	9	342	368	438	674
Total	953.0 (185.3)	24	842	927	1,024	1,577
Monthly deaths among older people						
Male	346.9 (58.5)	240	311.5	331	384.5	606
Female	316.7 (61.9)	217	278.5	299	347.5	585
Total	666.1 (121.9)	497	580.5	649	734.5	1,191

Three temperature measures were strongly correlated with each other with a correlation coefficient of between 0.98 and 0.99, but they were not strongly correlated with humidity or rainfall. Among all weather variables, the correlations showed that the three temperature indicators were negatively correlated with mortality with almost similar correlation coefficient estimates. The coefficients ranged from −0.33 to −0.46 with *p*-values less than 0.01 (Table 2).

**Table 2:** Spearman correlation coefficients between mortality and weather variables

	All-cause death: ages 60+	All-cause death: males ages 60+	All-cause death: females ages 60+
T_mean_	−0.42	−0.35	−0.45
T_min_	−0.39	−0.33	−0.43
T_max_	−0.42	−0.36	−0.46
Humidity	0.11	0.09	0.11
Rainfall	−0.06	−0.04	−0.08

Monthly seasonal variation in the death counts among ages 60+ and in temperature is illustrated in Fig. 1. The plot shows that the high peak of mortality coincided with low temperatures in the month of February 2008, during which the mean temperature was the lowest in the whole study period.

**Fig. 1:** Distribution of monthly reported death cases and mean temperature for the period 2005–2010.

The results from the GAM model (Fig. 2) show that there is a significant relationship between mean monthly temperature and mortality among older people (*p*<0.01). The model also shows that there were significant seasonal patterns in the older population's mortality, but no trend in mortality was discernible in this age group. Figure 2 illustrates the nonlinear relationship between temperature and risk of death among older people at the monthly time scale. The curve shows cubic splines of temperature with 2.36 degrees of freedom from the GAM model after controlling for time trend (edf=1.60) and seasonality (month of the year; edf=1.94). The observed relationship implies that a decrease in temperature is associated with an increase in mortality risk.

**Fig. 2:** The relationship between monthly mean temperature and all-causes mortality among older people in Hanoi, Vietnam, for the period 2005–2010, adjusted for time trend and seasonality.

The results for quantifying the risk related to the cold season are presented in Table 3. We considered the interaction between the period and season, but it was not significant. Therefore, the result for a model without the interaction was presented. Compared to the year 2005, the death cases in 2009 decreased significantly (IRR [incidence relative risk]=0.84, 95% CI=0.77–0.92, *p*<0.05), whereas other years were not significantly different. The mortality risk was 21% higher among the older population during the cold season compared to the warm season. This estimate corresponds to the winter excess mortality. The risk was higher among female older people compared to male ones (male: IRR=1.18; female: IRR=1.23).

**Table 3:** The incidence relative risks (IRRs) between period, season, and death cases in Hanoi, Vietnam, 2005–2010

	Total death cases			Male death cases			Female death cases		
	
	IRR	*p*	95% CI	IRR	*p*	95% CI	IRR	*p*	95% CI
Period (reference: 2005)									
2006	0.95	0.205	0.87–1.03	0.97	0.503	0.89–1.05	0.96	0.414	0.87–1.06
2007	0.96	0.400	0.88–1.05	0.99	0.783	0.91–1.07	0.98	0.714	0.89–1.08
2008	1.03	0.525	0.94–1.12	1.04	0.351	0.96–1.12	1.07	0.165	0.97–1.18
2009	**0.84**	**0.000**	**0.77–0.92**	**0.86**	**0.000**	**0.79–0.93**	**0.87**	**0.003**	**0.79–0.95**
2010	1.01	0.769	0.93–1.10	1.05	0.252	0.97–1.13	1.02	0.627	0.93–1.13
Cold season (reference: warm season)	**1.21**	**0.000**	**1.15–1.28**	**1.18**	**0.000**	**1.12–1.23**	**1.23**	**0.000**	**1.16–1.29**

Bold values denotes for significant difference with *p*<0.001.

## Discussion

This study contributes to an understanding of the temperature–mortality relationship in developing countries. We observed the seasonal effect on mortality among the older population in the capital of Vietnam. The study also found that older people are more susceptible to the effects of cold weather and that females are at greater risk. The observed excess mortality during cold weather in the study area of Vietnam was higher than that observed in Scandinavian countries but lower than that in some Southern European countries (36). However, we acknowledge the difference in time scale between our study and the previous studies, which may contribute to the size of the effect estimate.

In 2009, there was a significant drop in the number of deaths of older people as compared to other years during the study period. This could be due to excess deaths that were observed during the cold period in 2008. In fact, there were 8,935 death cases among the older population reported in 2008, and the cause of high mortality was circulatory diseases at highest level (accounted 26.72%). Some studies have investigated a similar mortality displacement effect due to weather extremes (15, 49–51). However, there were varied findings from these studies about the presence of mortality displacement. Mortality displacement has not been found due to the immediate impact of weather extremes in the United States (15, 51). Our finding of death displacement implies that temperature might serve as an indicator for predicting mortality displacement in Vietnam but may need further investigation (23, 52).

The study found that there was a statistically significant relationship between seasonality and mortality among tzhe older people of Hanoi, Vietnam. We also found significant cold-related mortality among these older persons. This finding is consistent with previous studies where the cold season was associated with a high number of deaths among older people (23, 36, 53, 54). The result is similar to a study in the United States that found that overall death rates are higher in winter than in summer (13). A similar relationship of cold-related mortality among older people was observed in Nairobi, though it was not statistically significant (2). Cold temperature has been reported in previous studies to contribute to cardiovascular-related mortality (18, 20, 23, 24, 55). It has been shown that younger people and people with hyperglycemia could enhance susceptibility to cold temperature, whereas old age limits this ability (1, 8). This is true in our study when circulatory disease is the leading cause of death under the study period (23.5% of total death) among older people. Our findings suggest that temperature-related mortality in the cold seasons is a significant public health issue in countries with tropical climates like Vietnam. The finding may have implications for developing intervention strategies to reduce temperature-related impacts. Such strategies could include improving housing conditions and providing insulation or heating facilities during the cold season. A study of cause-specific mortality and temperature relationships among the older people in Vietnam is necessary for understanding the relationship.

However, some studies have also found heat-related mortality (3, 4, 26, 49, 50, 56), and this difference might be explained by differences in time scale for the temperature–mortality data analyzed. Previous studies used the daily data to measure the association between temperature and mortality, but in our study, we used monthly aggregated data that might obscure the relationship between temperature and mortality in heat waves. This suggests that future studies should seek to use daily data in studying refined temperature–mortality relationships.

Our study also found that females were at higher risk of death in cold weather than males. Studies in Europe (3) and China (29), including Hong Kong (7, 8), found that females were at greater risk in high temperature, but so far, none of the studies reported the higher risk of females during cold seasons. Nevertheless, the existing evidence seems to indicate that weather-related impacts are more pronounced among females compared to males. The lower capability of producing maximum heat vasoconstriction puts females at greater risk during cold spells (57–59). The result suggests that the cold–mortality relationship among females needs further investigation to establish possible factors explaining the association.

## Conclusions

This study establishes the temperature–mortality relationship and quantifies seasonality in mortality among older people in Hanoi, Vietnam. The winter excess mortality in the study region can be compared to estimates from Central and Southern European populations. Older people are more susceptible to cold weather, and females appear to be at greater risk. Attention should be provided to protect vulnerable subpopulations from daily weather variations. A comfortable temperature in living conditions and increased attention to vulnerable groups during the cold weather extremes are necessary preventive measures.

However, we acknowledge some limitations of our study. Firstly, the study used monthly data for mortality, which might not be the ideal, and therefore we were not able to establish lag effects of temperature. Despite this limitation, the study still provides some evidence toward understanding weather variability health impacts. In addition, other factors are known to be associated with mortality, such as living conditions, family and social support, access to medical care (14), and indicators of socioeconomic status of the city population (53); these were not adjusted for the current study due to lack of information. Finally, our analysis did not include other factors that may influence the temperature–mortality relationship. We acknowledged this limitation because the data on mortality were available in aggregate form. However, we do not expect the results to differ much since we controlled for season and trend in the time-series model. This is because for the time-series model, the results are not affected by not including variables that vary at the time scale used for modeling. For example, including rainfall is not likely to change the results. Overall, the study provides a relevant contribution to the research topic.

## References

[1] Atsumi A, Ueda K, Irie F, Sairenchi T, Iimura K, Watanabe H (2013). Relationship between cold temperature and cardiovascular mortality, with assessment of effect modification by individual characteristics. Circ J.

[2] Egondi T, Kyobutungi C, Kovats S, Muindi K, Ettarh R, Rocklov J (2012). Time-series analysis of weather and mortality patterns in Nairobi's informal settlements. Glob Health Action.

[3] Ishigami A, Hajat S, Kovats RS, Bisanti L, Rognoni M, Russo A (2008). An ecological time-series study of heat-related mortality in three European cities. Environ Health.

[4] Pan WH, Li LA, Tsai MJ (1995). Temperature extremes and mortality from coronary heart disease and cerebral infarction in elderly Chinese. Lancet.

[5] Rofi A, Doocy S, Robinson C (2006). Tsunami mortality and displacement in Aceh province, Indonesia. Disasters.

[6] Tomari T, Yanagihashi T, Wakisaka I, Uda H, Torimaru H (1991). [Seasonal variation of mortality from cerebro-cardiovascular diseases – effect of ambient temperature on death]. Nihon Koshu Eisei Zasshi.

[7] Xu W, Thach TQ, Chau YK, Lai HK, Lam TH, Chan WM (2013). Thermal stress associated mortality risk and effect modification by sex and obesity in an elderly cohort of Chinese in Hong Kong. Environ Pollut.

[8] Yan YY (2011). Association between daily mortality and weather in Hong Kong. Internet J Public Health.

[9] Yu W, Mengersen K, Wang X, Ye X, Guo Y, Pan X (2012). Daily average temperature and mortality among the elderly: a meta-analysis and systematic review of epidemiological evidence. Int J Biometeorol.

[10] Guo Y, Barnett AG, Pan X, Yu W, Tong S (2011). The impact of temperature on mortality in Tianjin, China: a case-crossover design with a distributed lag nonlinear model. Environ Health Perspect.

[11] Basu R (2009). High ambient temperature and mortality: a review of epidemiologic studies from 2001 to 2008. Environ Health.

[12] Bouchama A, Knochel JP (2002). Heat stroke. N Engl J Med.

[13] McGeehin MA, Mirabelli M (2001). The potential impacts of climate variability and change on temperature-related morbidity and mortality in the United States. Environ Health Perspect.

[14] Stafoggia M, Schwartz J, Forastiere F, Perucci CA, Group S (2008). Does temperature modify the association between air pollution and mortality?. A multicity case-crossover analysis in Italy. Am J Epidemiol.

[15] Basu R, Malig B (2011). High ambient temperature and mortality in California: exploring the roles of age, disease, and mortality displacement. Environ Res.

[16] El-Zein A, Tewtel-Salem M, Nehme G (2004). A time-series analysis of mortality and air temperature in Greater Beirut. Sci Total Environ.

[17] Revich B, Shaposhnikov D (2008). Excess mortality during heat waves and cold spells in Moscow, Russia. Occup Environ Med.

[18] Revich B, Shaposhnikov D (2008). Temperature-induced excess mortality in Moscow, Russia. Int J Biometeorol.

[19] Revich BA, Shaposhnikov DA, Semutnikova EG (2008). [Climate conditions and ambient air quality as risk factors for mortality in Moscow]. Med Tr Prom Ekol.

[20] Rocklov J, Ebi K, Forsberg B (2011). Mortality related to temperature and persistent extreme temperatures: a study of cause-specific and age-stratified mortality. Occup Environ Med.

[21] Rocklov J, Forsberg B (2008). The effect of temperature on mortality in Stockholm 1998–2003: a study of lag structures and heatwave effects. Scand J Public Health.

[22] Rocklov J, Forsberg B (2010). The effect of high ambient temperature on the elderly population in three regions of Sweden. Int J Environ Res Public Health.

[23] Rocklov J, Forsberg B, Meister K (2009). Winter mortality modifies the heat-mortality association the following summer. Euro Resp J.

[24] Gouveia N, Hajat S, Armstrong B (2003). Socioeconomic differentials in the temperature–mortality relationship in Sao Paulo, Brazil. Intl J Epidemiol.

[25] Kim H, Ha JS, Park J (2006). High temperature, heat index, and mortality in 6 major cities in South Korea. Arch Environ Occup Health.

[26] Kim Y, Joh S (2006). A vulnerability study of the low-income elderly in the context of high temperature and mortality in Seoul, Korea. Sci Total Environ.

[27] Carder M, McNamee R, Beverland I, Elton R, Cohen GR, Boyd J (2005). The lagged effect of cold temperature and wind chill on cardiorespiratory mortality in Scotland. Occup Environ Med.

[28] Guo Y, Barnett AG, Tong S (2012). High temperatures-related elderly mortality varied greatly from year to year: important information for heat-warning systems. Sci Rep.

[29] Yang J, Liu HZ, Ou CQ, Lin GZ, Zhou Q, Shen GC (2013). Global climate change: impact of diurnal temperature range on mortality in Guangzhou, China. Environ Pollut.

[30] Vutcovici M, Goldberg MS, Valois MF (2013). Effects of diurnal variations in temperature on non-accidental mortality among the elderly population of Montreal, Quebec, 1984–2007. Int J Biometeorol.

[31] Chung JY, Honda Y, Hong YC, Pan XC, Guo YL, Kim H (2009). Ambient temperature and mortality: an international study in four capital cities of East Asia. Sci Total Environ.

[32] Armstrong B (2006). Models for the relationship between ambient temperature and daily mortality. Epidemiology.

[33] Krstic G (2011). Apparent temperature and air pollution vs. elderly population mortality in Metro Vancouver. PLoS One.

[34] Ballester F, Corella D, Perez-Hoyos S, Saez M, Hervas A (1997). Mortality as a function of temperature. A study in Valencia, Spain, 1991–1993. Int J Epidemiol.

[35] Basu R, Dominici F, Samet JM (2005). Temperature and mortality among the elderly in the United States: a comparison of epidemiologic methods. Epidemiology.

[36] Healy JD (2003). Excess winter mortality in Europe: a cross country analysis identifying key risk factors. J Epidemiol Community Health.

[37] Mercer JB (2003). Cold – an underrated risk factor for health. Environ Res.

[38] Boardmand B (1991). Fuel poverty: from cold homes to affordable warmth.

[39] Group TE (1997). Cold exposure and winter mortality from ischaemic heart disease, cerebrovascular disease, respiratory disease, and all causes in warm and cold regions of Europe. The Eurowinter Group. Lancet.

[40] Lipsitch M, Viboud C (2009). Influenza seasonality: lifting the fog. Proc Natl Acad Sci U S A.

[41] Vietnam Ministry of Natural Resources and Environment (MONRE) (2011). National climate change strategy.

[42] United Nations (2011). Climate change fact sheet: the effects of climate change in Vietnam and the UN's responses.

[43] Ministry of natural resources and environment (2009). Climate change, Sea level Rise Scenarios for Vietnam.

[44] ACCRN (2009). HCVA in Can Tho: hazard, capacity & vulnerability assessment in relation to climate change.

[45] GSO (2009). Population census report.

[46] Toan Do TT, Hu W, Quang Thai P, Hoat LN, Wright P, Martens P (2013). Hot spot detection and spatio-temporal dispersion of dengue fever in Hanoi, Vietnam. Glob Health Action.

[47] Stata statistical software (2007). Release 10.

[48] McCullagh P, Nelder JA (1989). Generalized linear models.

[49] Hajat S, Armstrong BG, Gouveia N, Wilkinson P (2005). Mortality displacement of heat-related deaths: a comparison of Delhi, Sao Paulo, and London. Epidemiology.

[50] Heaton MJ, Peng RD (2012). Flexible distributed lag models using random functions with application to estimating mortality displacement from heat-related deaths. J Agric Biol Environ Stat.

[51] Zanobetti A, Schwartz J (2008). Mortality displacement in the association of ozone with mortality: an analysis of 48 cities in the United States. Am J Respir Crit Care Med.

[52] Fouillet A, Rey G, Wagner V, Laaidi K, Empereur-Bissonnet P, Le Tertre A (2008). Has the impact of heat waves on mortality changed in France since the European heat wave of summer 2003?. A study of the 2006 heat wave. Int J Epidemiol.

[53] Curriero FC, Heiner KS, Samet JM, Zeger SL, Strug L, Patz JA (2002). Temperature and mortality in 11 cities of the eastern United States. Am J Epidemiol.

[54] Lin YK, Ho TJ, Wang YC (2011). Mortality risk associated with temperature and prolonged temperature extremes in elderly populations in Taiwan. Environ Res.

[55] Yu W, Hu W, Mengersen K, Guo Y, Pan X, Connell D (2011). Time course of temperature effects on cardiovascular mortality in Brisbane, Australia. Heart.

[56] Almeida SP, Casimiro E, Calheiros J (2010). Effects of apparent temperature on daily mortality in Lisbon and Oporto, Portugal. Environ Health.

[57] Astrand I (1960). Aerobic work capacity in men and women with special reference to age. Acta Physiol Scand Suppl.

[58] Burse RL (1979). Sex differences in human thermoregulatory response to heat and cold stress. Hum Factors.

[59] Buskirk ER, Thompson RH, Whedon G (1963). Metabolic responses to cold air in men and women in relation to total body fat content. J Appl Phys.
